# Supplementary material for: Perspectives on factors influencing transmission of COVID-19 in Zambia: a qualitative study of health workers and community members
Source: BMJ Open. 2022 Apr 4;12(4):e057589. doi: 10.1136/bmjopen-2021-057589 (PMC8983411; doi:10.1136/bmjopen-2021-057589)
Supplement: Supplementary data [file bmjopen-2021-057589supp001.pdf]

INFORMED CONSENT FORM/INFORMATION SHEET

Background

Hello, my name is ___________________________ I am working with the Levy Mwanawasa Medical University and Eden University. This study is funded by the National Science Technology Council.

Purpose

We would like to speak with you because you are ……….. We would like to understand your opinion and perspectives on the ***sociocultural dynamics influencing transmission of coronavirus in Zambia.*** This research is taking place in Lusaka and Chirundu districts. What you tell us may help the Ministry of Health and government as they try to reduce transmission of ***coronavirus*** among the people who live in this community.

Process

You will be one of approximately 65 respondents asked to participate in this discussion. We would like to ask you some questions in order to understand your opinions and perspectives on the ***sociocultural dynamics influencing transmission of coronavirus.*** The discussion should take no longer than 1.5 hours.

To ensure that I don't miss or forget anything during the discussion, your responses to the various questions will be recorded using a digital recorder. We will not write your name, instead we ask you to sign on this form, but we will keep your signature private. Responses from the various questions will be summarised, analysed and a report written. When we write reports from this discussion, we will not show what you said nor are we going to use your name or signature at any time.

Potential Benefits

You will receive no direct benefit from your participation in this discussion. However, your participation may help the Ministry of Health and the government as they try to reduce transmission of ***coronavirus*** in the country

Risks and Discomforts

The risks of taking part in this study are that other people will hear your responses. It is important that you do not share anything that you are not comfortable with. If you or someone in your family had a bad experience, it may be difficult or uncomfortable to remember or share it. You do not have to respond to any question unless you feel comfortable doing so. You are free to stop the discussion at any time if you need to.
.

Alternatives

You can choose not to take part in this discussion. If you decide not to take part or withdraw from this discussion, you will not suffer any penalty or lose any benefits to which you are entitled.

Participant Costs and Payments

You will not be paid to participate in this discussion. There are no costs to you for participating.

Confidentiality

Sialubanje C, *et al. BMJ Open* 2022; 12:e057589. doi: 10.1136/bmjopen-2021-057589

INFORMED CONSENT FORM/INFORMATION SHEET

Your information will be saved and locked in a computer file and destroyed once it is copied from it. Only study team members will be able to listen to your response. When we write reports from this data, we will not link your signature or identity to your responses.

Participant's Rights

By agreeing to participate in this discussion, you do not waive any of your legal rights. Giving consent means that you have heard or read the information about this discussion and that you agree to participate. You will be given a copy of this form to keep. If you have questions or concerns at any time, you can contact the Principal Investigator, Dr. Sialubanje Cephas on +260-977-441273, the Co-Investigator, Prof Fastone Goma on +260 977 772 301, or any of the staff from the University of Zambia Biomedical Research Ethics Committee on +260-21-1-256067

Right to Refuse or Withdraw

Taking part in this discussion is voluntary. You have the right to refuse to take part. If you decide to be in this discussion, and then change your mind, you can withdraw from it at any time and to skip questions you may deem personal or otherwise without any repercussions. Your participation is voluntary. If you choose to take part, you have the right to stop the discussion at any time.

By signing below you are agreeing to participate in the discussion which indicates that you have read this consent form (or have had it read to you), that your questions have been answered to your satisfaction, and that you voluntarily agree to participate in this research study. You may keep a copy of this for your records.

Signature or thumb print: ____

Date: ____

Signature of Impartial Witness: ____

Date: ____
