# Supplementary material for: Perspectives on factors influencing transmission of COVID-19 in Zambia: a qualitative study of health workers and community members
Source: BMJ Open. 2022 Apr 4;12(4):e057589. doi: 10.1136/bmjopen-2021-057589 (PMC8983411; doi:10.1136/bmjopen-2021-057589)
Supplement: Supplementary data [file bmjopen-2021-057589supp002.pdf]

INTERVIEW GUIDE

……………………………………………………………………………………………………..

Theme 1: Perspectives on COVID-19 preventive measures (social distancing, wearing masks and handwashing)

Q1: Please tell me what you think about the measures government has put in place to prevent transmission of COVID-19

Probe:

- Awareness about COVID-19
- Knowledge about the measures (social distancing, wearing face masks, hand washing)
- Participant's attitude towards these measures (advantages and disadvantages)
- Perspectives on these measures (are the community members adhering to them)

Theme 2: Livelihood and way of life

Q2: Please tell me about your way of life

Probe:

- Family and living conditions (place of residence, number of people at home, number of rooms in the house, condition of the house)
- Source of drinking water (borehole, river, presence of running water)
- Hand sanitising facilities at home
- Occupation/main source of family income
- Working conditions (office space; office shared or not; number of people in the office; use of lifts; toilet facilities, hand sanitising facilities at work)
- Mode of transport to place of work

Theme 3: Travel & movement patterns

Q3: Would you walk me through your recent travel history (past one month)

Probe:

- Movement from home on a typical week day
- Movement from home on a typical weekend day
- Places the respondent visited in the past one month within and outside Zambia
- Duration of stay away from home
- Mode of transport commonly uses/used
- How often the respondent travels/traveled outside home
- The purpose (s) of travel
- People who travel with respondent
- Mode, if any, of protecting oneself against COVID-19 during travel

Theme 4: Social networks

Q4: Walk me through the most important people (family members, friends, relatives, workmates, church mates,) that you have interacted with in the past one month

Probe:

- Most important people frequently in contacts with outside home
- How do you maintain contact with these people (family, friends, workmates, church mates)
- Most recent social activities, meetings, church meetings, weddings, funerals attended in the past one month
- Physical contact with friends & family during social gatherings (i.e. shaking hands, sharing cigarettes/drinks, sharing eating utensils, sharing accommodation/ hotel room)
- Adjustments in physical interaction with family members, friends, workmates (physical/social distancing, working from home, worshipping from home, visits)
- Perspectives on social distancing

THE END
