# Supplementary material for: Perspectives on factors influencing transmission of COVID-19 in Zambia: a qualitative study of health workers and community members
Source: BMJ Open. 2022 Apr 4;12(4):e057589. doi: 10.1136/bmjopen-2021-057589 (PMC8983411; doi:10.1136/bmjopen-2021-057589)
Supplement: Supplementary data [file bmjopen-2021-057589supp003.pdf]

Demographic questionnaire

Questionnaire ID number [ ]

Name of District……………………………………….

---

1. Place of residence (indicate name)………………………………..
2. Number at home (indicate actual number)………………………..
3. Age (Indicate the actual age in years)…………………………
4. Number of children (indicate the actual number)…………………..
5. Marital status (Circle one appropriate answer)…………
   1) Single
   2) Separated
   3) Married
   4) Widow
   5) Cohabiting
6. Religious denomination (indicate appropriate answer)……………..
   1) Catholic
   2) SDA
   3) Pentecostal
   4) Jehovah's witness
   5) Other (specify)…………..
7. Level of education (highest grade reached) (Circle appropriate answer)
   1) Never attended school
   2) 1-4
   3) 5-7
   4) 8-9
   5) 10-12
   6) College
   7) University
8. Occupation (indicate)…………………….
9. Level of income per month (in Zambian Kwacha) (Circle appropriate box)………..
   1) <K500
   2) K500-999
   3) 1000-1499
   4) K1,500-1999
   5) >2000
10. Travel out of town in the past one month……..
    1) Yes
    2) No

11. If yes to Q19, mention the place you travelled to (indicate name)…………………..

12. Have you travelled out of the country in the past one month? ……………………
    1) Yes
    2) No

13. If yes to Q12, mention the place you travelled to (indicate name of place)……………….

14. Mention the date you travelled (indicate the actual date ………………..

15. Mention the date you returned (indicate the actual date)………………..

16. How long did you stay there (indicate actual duration in days)…………
