# Supplementary material for: Perspectives on factors influencing transmission of COVID-19 in Zambia: a qualitative study of health workers and community members
Source: BMJ Open. 2022 Apr 4;12(4):e057589. doi: 10.1136/bmjopen-2021-057589 (PMC8983411; doi:10.1136/bmjopen-2021-057589)
Supplement: Author's manuscript [file bmjopen-2021-057589.draft_revisions.pdf]

BMJ Open is committed to open peer review. As part of this commitment we make the peer review history of every article we publish publicly available.

When an article is published we post the peer reviewers' comments and the authors' responses online. We also post the versions of the paper that were used during peer review. These are the versions that the peer review comments apply to.

The versions of the paper that follow are the versions that were submitted during the peer review process. They are not the versions of record or the final published versions. They should not be cited or distributed as the published version of this manuscript.

BMJ Open is an open access journal and the full, final, typeset and author-corrected version of record of the manuscript is available on our site with no access controls, subscription charges or pay-per-view fees (http://bmjopen.bmj.com).

If you have any questions on BMJ Open's open peer review process please email info.bmjopen@bmj.com

BMJ Open

Perspectives on Factors Influencing Transmission of Coronavirus 19 (COVID-19) in Zambia: An Exploratory Study of Health Workers and Community Members

Journal:	*BMJ Open*
Manuscript ID	bmjopen-2021-057589
Article Type:	Original research
Date Submitted by the Author:	25-Sep-2021

Complete List of Authors:	Sialubanje, Cephas; Levy Mwanawasa Medical University, School of Public Health<br>Sitali, Doreen; University of Zambia, School of Public Health<br>Mukumbuta, Nawa; Levy Mwanawasa Medical University, School of Public Health<br>Liyali, Libonda; Eden umiversity, School of medicine<br>Goma, Fastone; Eden university, School of Medicine

Keywords:	COVID-19, INFECTIOUS DISEASES, Public health < INFECTIOUS DISEASES

SCHOLARONE™
Manuscripts

BMJ

I, the Submitting Author has the right to grant and does grant on behalf of all authors of the Work (as defined in the below author licence), an exclusive licence and/or a non-exclusive licence for contributions from authors who are: i) UK Crown employees; ii) where BMJ has agreed a CC-BY licence shall apply, and/or iii) in accordance with the terms applicable for US Federal Government officers or employees acting as part of their official duties; on a worldwide, perpetual, irrevocable, royalty-free basis to BMJ Publishing Group Ltd ("BMJ") its licensees and where the relevant Journal is co-owned by BMJ to the co-owners of the Journal, to publish the Work in this journal and any other BMJ products and to exploit all rights, as set out in our licence.

The Submitting Author accepts and understands that any supply made under these terms is made by BMJ to the Submitting Author unless you are acting as an employee on behalf of your employer or a postgraduate student of an affiliated institution which is paying any applicable article publishing charge ("APC") for Open Access articles. Where the Submitting Author wishes to make the Work available on an Open Access basis (and intends to pay the relevant APC), the terms of reuse of such Open Access shall be governed by a Creative Commons licence – details of these licences and which Creative Commons licence will apply to this Work are set out in our licence referred to above.

Other than as permitted in any relevant BMJ Author's Self Archiving Policies, I confirm this Work has not been accepted for publication elsewhere, is not being considered for publication elsewhere and does not duplicate material already published. I confirm all authors consent to publication of this Work and authorise the granting of this licence.

For peer review only

Perspectives on Factors Influencing Transmission of Coronavirus 19 (COVID-19) in Zambia: An Exploratory Study of Health Workers and Community Members

Cephas Sialubanje[1*], Doreen C Sitali[2], Nawa Mukumbuta[3], Libonda Liyali[4], Fastone M. Goma[4]

[1]Department of Disease Prevention, Levy Mwanawasa Medical University, School of Public Health, P.O. Box 33991, Lusaka, Zambia

[2]Department of Health Promotion, University of Zambia, School of Public Health, P.O. Box 32379, Lusaka, Zambia

[3]Department of Biostatistics and Epidemiology. School of Public Health and Environmental Studies. Levy Mwanawasa Medical University. P. O. Box 33991, Lusaka. Zambia 10101.

[4]Department of Disease Control and Prevention, School of Medicine and Health Sciences, Eden University, P.O. Box 37727, Lusaka, Zambia

*Corresponding Author: Cephas Sialubanje; csialubanje@yahoo.com

Mobile: +260 977 441 273

For peer review only

For peer review only - http://bmjopen.bmj.com/site/about/guidelines.xhtml

Abstract

Objective: To explore the health professionals' and community members' perspectives on the factors influencing transmission of the novel coronavirus disease 2019 (COVID-19) in Zambia.

Design: An exploratory qualitative study using in-depth interviews as data collection technique.

Setting: Four primary health care facilities and local communities of Lusaka city and Chirundu international border town under Lusaka province, Zambia

Participants: Purposive sampling of sixty study participants comprising health professionals (n=15) and community members (n=45). Health staff were health inspectors and surveillance officers. Community members included public market traders, civic and religious leaders, immigration officers, bus and international truck drivers.

Results: Both health professionals and community members were aware of the COVID-19 pandemic and the preventive and control measures. Nevertheless, there were stark differences on the two groups' perspectives on COVID-19 and the factors influencing its transmission. Most health staff perceived high personal risk and susceptibility to the disease, and expressed a positive attitude towards the prevention and control measures. In contrast, myths and misconceptions about the disease influenced most community members' perspectives on the existence, severity of and personal susceptibility to the disease. Further, these participants expressed a negative attitude towards the preventive and control measures. Both groups of participants unanimous on the low levels of adherence to the COVID-19 preventive and control measures in the community. Reasons for non-adherence included social movement and travel patterns, living and working conditions, social gatherings and mixing patterns, networks and interactions, and observing politicians' and other important community leaders' conduct. These factors were perceived to increase the risk of COVID-19 transmission.

Conclusion: These findings highlight important factors influencing transmission of COVID-19 in Zambia. Future interventions should focus on providing information to mitigate myths and misconceptions, increasing people's risk perception and personal susceptibility to the disease, and improving attitude towards the prevention and control interventions.

Word count: 299

Key words: COVID-19, transmission, factors, qualitative research, Zambia

For peer review only

Strengths and Limitations of the study

- The study provides the first in-depth assessment and analysis of the health professionals' and community members' perspectives on the factors influencing transmission of the virus in the two districts that were hotspots for COVID-19 hotspots during the first wave in Zambia
- Our purposive selection of health workers and community members from both urban and rural sites, with varied socioeconomic backgrounds and experiences with regard to COVID-19, as well as data collection and analysis conducted by a trained and experienced team allowed for triangulation, minimised bias and increased the internal validity of the study findings
- The study findings provide important targets for policy and intervention design for prevention of COVID-19 transmission in Zambia and other countries with similar demographic and epidemiological contexts
- The study was conducted during the first COVID-19 wave when the disease was still new and the number of cases and deaths was still low in the country; it is not clear whether community members' perspectives have changed over the three successive waves of COVID-19 experienced in the country.

For peer review only

For peer review only - http://bmjopen.bmj.com/site/about/guidelines.xhtml

Background

Severe Acute Respiratory Syndrome Coronavirus 2 (SARS-CoV-2), a novel coronavirus disease which emerged in Wuhan, Hubei, China in early December 2019 has now spread around the world over [1-3]. The outbreak was reported to the World Health Organisation (WHO) country office on 31st December, 2019 [4-6] which named the new coronavirus disease as Coronavirus Disease of 2019 (COVID-19) [7-9]. Following reports of increased spread and number of confirmed cases and deaths, on 30th January, WHO declared the outbreak a Public Health Emergency of International Concern [7]. With more countries affected, on 11th March, the WHO Director General described COVID-19 as a pandemic [7]. With the disease being reported among health workers and other patients with no history of contact with sea food, the mode of transmission of the virus was established as person-to-person through surfaces, droplets and aerosols produced during coughing [10, 11]. As of 25th August, 2021, more than 213 million cases and 4.45 million deaths have been reported globally [3].

The first confirmed cases of COVID-19 in Zambia were reported on 18th March, 2020 and involved a Zambian couple that had travelled and stayed in France for 10 days [12]. Since then, clusters of confirmed cases were reported from Lusaka city and Kafue town. Before long, cases were reported from the Copperbelt province, Nakonde and Chirundu borders and eventually from all the ten provinces in the country. To date, more than 200 000 cases and 3,500 deaths have been reported in the country [12]. Following reports of the initial cases, the Zambian government enacted statutory instrument number 22 [13] and instituted prevention control measures including social distancing, restriction of social gatherings by closing schools, bars and casinos, restaurants and other businesses; wearing of face masks, washing hands and sanitising. Messages on COVID-19 transmission, prevention and control were disseminated through mass media. Other measures included restriction of international travel as well as closing of international airports and restricting flights to one (Kenneth Kaunda) international airport; screening and testing; contact tracing, quarantine and isolation, and case management. The restrictive measures were intended to reduce the spread of the virus through social mixing and person-to-person contact which promote spread of infectious diseases, including COVID-19 [2].

Social distancing and restriction of social gathering measures have often been used in epidemics [2] because they encourage the general public to avoid crowded places and drastically shift social mixing patterns [2,15]. For example, a study conducted in China by Qui and colleagues [14] showed that social mixing patterns affect the trajectory of the outbreak within the local community. The study also showed that social mixing and networks, frequency of contacts in different age groups and locations−such as schools, churches, workplaces, households, bars, and other social gatherings−have a direct effect on the

For peer review only - http://bmjopen.bmj.com/site/about/guidelines.xhtml

time-dependent and basic reproduction numbers facilitating SARS-CoV-2 transmission in the community [15,16].

Currently, there is limited evidence on the extent to which these interventions are adhered to in the community. Moreover, community members' perspectives and attitude towards these measures are not known. The aim of this study was to explore the health workers' and community members' perspectives on the factors influencing transmission of COVID-19 in Zambia. Information is needed to inform design and implementation of interventions focusing on reducing transmission of the virus in the country.

Methods

Study design

An exploratory qualitative study was conducted using in-depth interviews (IDIs) as a data collection technique over a period of twelve weeks, from 3rd August to 30th October, 2020. Use of IDIs allowed for a detailed exploration of the subject under investigation [17,18].

Study setting

The study was conducted in 4 primary health care facilities and their local communities: 3 from Lusaka city and 1 from Chirundu district under Lusaka province. Lusaka is the capital city of Zambia and has an estimated population of 2.9 million [19]. Chirundu is an international border town between Zambia and Zimbabwe. It is located 115km south-east of Lusaka city and has an estimated population of 127, 600 [19]. Selection of the two sites was done purposively; Lusaka was included in the study because it was an epicenter of the COVID-19 during the first wave. Chirundu town was selected because it was one of the COVID-19 hot spots in the country and it is a town of entry from the bordering countries to the southern African countries of Zimbabwe, Mozambique, Swaziland, Lesotho and South Africa−at the time, South Africa had the highest prevalence of COVID-19 in the region [20]. With regard to health services, Lusaka has 2 tertiary, 5 second level and several primary level health care facilities. In addition, there are private and faith-based health care providers. Chirundu has one faith-based second level hospital and several primary health care level facilities. The Ministry of Health headquarters and the Zambia National Public Health Institute (ZNPHI)−responsible for the COVID-19 policies, guidelines and disease intelligence are also located in Lusaka. Services provided include specialized and primary health care, including health promotion on COVID-19, screening, testing and contact racing, case management and vaccination.

Participants and sampling technique

For peer review only - http://bmjopen.bmj.com/site/about/guidelines.xhtml

For peer review only

A purposive sampling technique was used to select 60 study participants. Purposive sampling allows for selection of participants with similar experiences regarding the health problem under investigation (that is, COVID-19), while, at the same time, allowing for recruitment of participants with different demographic and socioeconomic characteristics−such as place of residence, occupation and income levels. This, in turn, helps provide insight into the similarities and differences in the participants' experiences with regard to the health problem under investigation [17,18].

Study participants comprised 15 health workers (7 nurses and 8 public health officers) and 45 community members (18 public market traders, 6 bus drivers and 6 international truck drivers, 3 civic leaders, 3 religious leaders, 3 immigration officers, and 6 community members who had recovered from COVID-19). Health workers were sampled from 4 primary health facilities (3 from Lusaka and 1 from Chirundu border town). Eight health professionals worked in the primary health facilities, four worked as port health staff at the Lusaka International Airport; and the remainder worked as surveillance officers at the Zambia National Public Health Institute (ZNPHI). Community members were recruited from the local communities under the four primary health facilities. International truck drivers were selected from Chirundu border; bus drivers were selected from Lusaka intercity bus station. In order to provide insight into the similarities and differences in the IDI participants' perspectives and lived experiences with COVID-19, we sought to balance the number of participants by place of residence and occupation [17, 18].

To be included into the study, participants needed to be:

- Aged above 18 years
- Residing in the selected area for more than 3 months (except for the international truck drivers sampled from Chirundu border)
- Bus drivers from Lusaka

Those who were aged less than 18 years and new in the area were not eligible to participate in the study.

Data collection procedures

Four research assistants were trained in interviewing techniques over a period of 5 days. The training comprised two phases: 3 days of theory and 2 days of practical fieldwork. Efforts were made to select research assistants who were skilled and experienced in qualitative research and spoke both English and the local languages, Nyanja, Bemba or Tonga. Interviews were conducted at the participant's preferred place including the place of residence or office. On average, each interview lasted between 1 hour and 1.5hrours. To ensure quality in data collection, a digital voice recorder was used. Each interview was conducted by a pair of research assistants: one facilitated the interview and took notes, the other one was in charge of the digital voice recorder. Before each interview, written informed consent

For peer review only - http://bmjopen.bmj.com/site/about/guidelines.xhtml

was obtained from each participant; those who could not read or write were asked to mark with an 'X'. To make it easy for the study participants to understand, the consent form (annex 1) was translated into the local language (Tonga and Nyanja). Before the actual interview, each respondent was asked to complete a short demographic questionnaire. Research assistants read the consent form and the questionnaire and filled it in for those who could not read.

Data collection instruments

Data was collected using a paper- based, unstructured interviewed guide (annex 2) that was developed by the research team. The interview guide had two pre-determined themes including 1) perspectives on COVID-19, 2) factors affecting transmission of COVID-19. In addition, a short questionnaire (annex 3) was prepared to collect participant demographic and socioeconomic data. To ensure internal validity, both the interview guide and the short questionnaire were pre-tested during research assistant training.

Biosafety

To ensure their safety, research team members and study participants were provided with disposable facemasks and hand sanitisers, which they were encouraged to use consistently during training, travel and data collection process. Moreover, research team members were encouraged to observe physical distancing during training and data collection.

Ethical approval

Ethical approval was obtained from the University of Zambia Biomedical Research Ethics Committee (UNZA BREC, ref 1132-2020); permission to conduct the study was obtained from the National Health Research Authority.

Data processing and analysis

Voice recordings from the IDIs were transcribed and translated into English by two research assistants who were proficient both in English and the local language. To check for accuracy, members of the research team back−translated 10% of the transcripts into the local language and back into English. These versions were then be compared for differences and similarities while listening to the original voice recording. After verification of accuracy in transcription and translation, each transcript was then thoroughly read by one research assistant while the other one listened to the corresponding voice recording. Each translated transcript was then compared with the hand-written field notes that the research assistants prepared during the IDIs. After proof-reading and making corrections, the transcripts were saved on a password-protected file computer kept by the PI for safety. The Word documents was

For peer review only - http://bmjopen.bmj.com/site/about/guidelines.xhtml

For peer review only

then imported into Nvivo 11 MAC for coding and analysis, and the categories and key sub-themes were identified. In order to make it easy to compare differences and similarities in the respondent perspectives by group (health worker versus community members) and residential area (urban versus rural), separate analyse were done. An inductive approach was used to analyse the qualitative data in N-Vivo QSR 12, and sub-themes were derived by content-analysis. The inductive approach ensured that sub-themes were derived from the pre-determined themes by content-analysis and grouping all similar statements made with respect to particular themes. Quantitative sociodemographic data was analysed using descriptive statistics.

Patient and public involvement

The study design was determined by the call for research proposals on COVID-19 research under the strategic research fund. Thus, the participants and the public were not directly involved in the conceptualisation and design of the study. However, selection of the primary health facilities and study participants was done in collaboration with the provincial and district health mangers. First, an inception meeting was held with the ZNPHI and Ministry of Health staff from the Lusaka Provincial Health Office and the two districts to present and discuss the design and objectives of the study. Next, pre-field meetings were held with the provincial and district managers to select primary health care facilities and local communities to be included in the study. Recruitment of both the participants (health workers and community members) was done by the by the primary health facility managers and local community leaders. Finally, a report was written and shared with key stakeholders, including the funding organisation and Ministry of Health. In addition, a dissemination meeting was held in Lusaka to share the results with the Ministry of Health and community leaders in the districts where the study was conducted.

Results

In this section, we present our study findings in three parts. In part one: summary of the participants' demographic characteristics; part two: health workers' and community members' perspectives on COVID-19; and in part three we present the factors which influence transmission of COVID-19.

Demographic characteristics

The majority (60%) of the participants were female, the mean age was just above 38.12 years, and they had on average between two and three children. Over half (65%) of the participants were married. One third of the participants (33.3%) had secondary school education, one-fifth (21.7%) had tertiary level education, and 1.67% had never attended school at all. Concerning level of monthly income, 20% had

For peer review only - http://bmjopen.bmj.com/site/about/guidelines.xhtml

an average income of less than K500 per month. Majority of the sample (51.7%) mentioned having had travelled out of town and 5% out of the country. The countries travelled to were South Africa, Zimbabwe and Demographic Republic of Congo. The detailed demographic and socioeconomic profile of the study participants is shown in table 1 below.

Table 1: Sociodemographic characteristics (n=60)

**Variable**	**Mean (SD) /n (%)**
Age	38.12 (12.08)
Children	2.9 (2.0)
Number at home	5.9 (3.35)
Sex	
Female	36 (60%)
Male	24 (40%)
Marital status	
Single	12 (20%)
Separated	4 (6.67%)
Married	39 (65%)
Widow	5 (8.33%)
District	
Lusaka	36 (60%)
Chirundu	24 (40%)
Group	
Health Worker	15 (25%)
Community member	45 (75%)
Level of education	
Never attended	1 (1.67%)
Lower primary (1-4)	4 (6.67%
Upper primary (5-7)	12 (20%)
Junior Secondary (8-9)	10 (16.67%)
Senior Secondary (10-12)	20 (33.33%)
College	8 (13.33%)
University	5 (8.33%)
Occupation	
Health worker	15 (25%)
Community member	45 (75.0%)
Level of income	
<K500	12 (20.0%)
K500-999	15 (25.0%)
K1000-1499	5 (8.33%)
K1, 500-1, 999	3 (5.0%)
>K2000	25 (41.67%)
Travelled out of town	
Yes	31 (51.67%)
No	29 (48.33%)
Travelled out of the Country	
Yes	3 (5.0%)
No	57 (95.%)

For peer review only

Countries travelled to	
South Africa, Zimbabwe, DRC	

Theme 1: Perspectives on COVID-19

In this theme, we present the findings on the health workers' and community members' perspectives on COVID-19 including awareness about the COVID-19, sources of information, myths and misconceptions about the disease and knowledge regarding preventive measures.

Awareness about COVID-19

All the 15 health workers and most (60%) community members (except some bus drivers and public market traders) from both study sites were aware of COVID-19 and explained that they had seen on TV how it had spread to many countries and how people from other countries had died from the disease.

"When you watch on TV you can see the massive deaths which have happened in other countries, it shows that there is COVID" (Community member, Immigration Officer, Chirundu district)

In contrast, some community members, especially public market traders and bus drivers from Lusaka mentioned that, although they had heard about people dying, they did not know whether the deaths were due to COVID-19 or not. They also explained that some people did not believe the disease existed because they had not seen it as they did with other diseases like Ebola, and that they would only believe if they saw someone dying from COVID-19. Asked whether they had heard the reports from the Ministry of Health on the number of people who had died in the country, these participants explained that, in Africa COVID-19 did not kill as many people as it did in other continents, and that if COVID-19 really existed in Zambia, most people would have died already.

There is nothing conclusive. I need to see the corpses. Not until I see a dead body of someone and they say this one has died from Covid-19" [Bus driver, Lusaka district].

Sources of information about COVID-19

Both health workers and community members who had heard about COVID-19 cited the Ministry of Health and the media (radio and TV) as the main sources of information on COVID-19. Especially the health workers, civic leaders and people who had recovered from COVID-19 explained that the information included the mode of transmission of the virus, the preventive and control measures and the need to adhere to the guidelines. On the contrary, some participants, especially community members interviewed in Chirundu, explained that it was difficult to access information on COVID-19. They also expressed ignorance with regard to the source of the COVID-19 and the symptoms it causes.

For peer review only - http://bmjopen.bmj.com/site/about/guidelines.xhtml

The doctor [Minister of Health] said it on TV. He said that the disease is real and that the number of people with COVID-19 in the country has increased [Community member, Lusaka district]

Myths and misconceptions about COVID-19

Study participants expressed different views with regard to beliefs about the existence and source of the disease. Most health workers believed that the disease was real and that it was caused by the virus spread through the air and contact with other people and objects. In contrast, community members, including public market traders and bus drivers expressed myths and misconceptions about the COVID-19. They believed that someone had created the disease in order to eliminate the African population, and that foreigners [Chinese] who were interested in opening mines in African countries, like Congo, brought the disease so that local people could die, and that, eventually, the foreigners would take over the mines. In addition, some community members including religious leaders believed that the disease was a mark of the beast recorded in the Bible (Revelation). They explained that most people did not believe COVID-19 was real, and that government was playing politics and that politicians were working with foreigners to gain their support and make money for themselves. Some attributed COVID-19 to radiation from cell phones and industries, and that most people died because of radiation from 5G Networks. Consequently, they wondered why they were being asked to test for the disease and to wear facemasks.

"I am completely confused, is it really a natural disease? I think it is a fabricated disease or something like that. It all came from the Chinse people, and that other people are just inheriting it" (Public market trader, Chirundu district).

Knowledge about COVID-19 Preventive Measures

Health workers and community members expressed different perspectives on their knowledge about preventive and control measures. Most health workers and community members from Lusaka expressed knowledge about the COVID-19 prevention and control guidelines. Health workers explained that they helped disseminate the knowledge in the communities where they worked from and encouraged people to adhere to the guidelines including wearing facemasks, social distancing and overcrowded places, hand washing and sanitising. On the contrary, most community members including those from Chirundu border expressed ignorance about the preventive and control measures. They explained it was difficult to get information about COVID-19 because of the limited access to TV, radio, internet and mobile phone network. Especially bus drivers and public market traders mentioned that they only hear from people in the community and that most information was incorrect.

"They tell us to wear face masks and wash our hands with soap, but most of us do not have anyone to explain to us" (Public Market Trader, Lusaka city)

For peer review only - http://bmjopen.bmj.com/site/about/guidelines.xhtml

Theme 2: Factors influencing transmission of COVID-19

In this section, we present the findings on the factors that influence transmission of COVID-19 in the community. They include living and working conditions, travel and social movement patterns, and social networks.

Living and working conditions

Both health workers and community members (i.e., those who believed in the existence of the disease) were unanimous on living and working conditions being important factors driving the COVID-19 transmission. Overcrowding in some homes and work places made it difficult to maintain social distance. Public health officers explained that staff shared the office space and majority did not have enough hand-washing or sanitising facilities; they only used one shared sanitizer and the hand wash. They added that some offices and homes did not have running water; where it was present, the supply was erratic water supply and made it difficult for people to follow the guidelines on hand-washing and other hygienic purposes.

*"**You know our tradition, parents use their own bedroom, then the kids, maybe there are 5 girls, and they use one bedroom. If there are 6 boys they will use one bedroom" [Immigration officer, Chirundu district***].

Attitude towards preventive measures

Participants expressed different attitudes towards the preventive measures. Health workers and some community members (international truck drivers and those who had recovered from or seen someone with COVID-19 expressed a positive attitude towards preventive measures. They explained that the measures were beneficial to the population. The main benefits mentioned were protection the virus, helping people maintain hygienic standards in the community by avoiding dirty and handshakes. They explained that, in the beginning, they never believed in the reality of the disease, but later noticed that even in Zambia, people started dying; some were being quarantined. Health workers explained that there was a need for people to follow the prevention and control guidelines from the Ministry of Health, especially during the cold season, when the risk of transmission of the virus was high.

"What I think is that COVID is there, so the most important thing is to just follow what they say we should do" ***[Public Health Officer, Lusaka district]***

In contrast, most community members expressed a negative attitude towards the COVID-19 preventive measures. They wondered why they were being asked to wear facemasks or avoid mixing with their friends or attending important social functions like church services, weddings or drinking parties. Some

For peer review only - http://bmjopen.bmj.com/site/about/guidelines.xhtml

participants also explained that it was not clear why even children were told to wash their hands or wear facemasks.

"To be honest people do not like these things, they do not follow because they say, it is a lie, Corona is not there. They are saying because there is no Corona so they do not wear the mask" [Community member, Lusaka district].

Adherence to preventive measures

Participants were unanimous on the people's non-adherence to preventive measures in the community−which was seen as one of the important factors contributing to the spread of COVID-19 in the population. Health workers observed that, although some people followed the guidelines−wore facemasks and used hand-sanitisers− majority did not. They had continued mixing and interacting as before, especially in the markets and buses. Asked why they did not adhere to the guidelines, most community members explained that people in their communities believed that preventive measures came from other countries and local people, especially Africans, were just being told to follow. They also explained that most people were discouraged by what they saw among political leaders who held meetings and political rallies without following the guidelines. They explained that politician played politics as they did not maintain social distance or wear facemasks when holding campaigns rallies. Similarly, most bus drivers confirmed that their passengers did not observe the guidelines when travelling on public transport.

Participants had consensus on the importance of social gatherings such as weddings, kitchen parties, church services, taverns and funerals as virus super spreader events. Nevertheless, they observed that nothing had changed with regard to social mixing patterns; people were used to their old way of life of greeting and hugging their friends when they met. They argued that people did not feel good to stand a meter or an arm apart because of the new rules. Similarly, participants, especially health workers, civic and religious leaders, confirmed that most families had continued visiting and inviting friends or extended family members to their homes for family and social functions such as parties and beer drinking. Although some people did not attend funerals and only provided monetary and other material support, many community members did. However, they argued that people wore facemasks in shops and during church services.

"The only time we can say social distance, is if we come to the market, in the shops, or church. There is no social distance when you go to the political rallies" [Religious leader, Lusaka district]

Travel & movement patterns

For peer review only - http://bmjopen.bmj.com/site/about/guidelines.xhtml

Both health workers and most community members perceive (except some bus drivers) confirmed that travel within and outside the country was a major factor influencing transmission of the virus. They observed that, despite the COVID-19 situation, local and international travel remained the same. Public transport on buses and minibuses was the commonest mode of transport. Those who owned cars explained that they travelled in their personal cars. Reasons cited for travel were work, business, shopping, family matters (such as funerals), and other purposes.

With regard to adherence to prevention and control measures on buses, bus drivers explained that although conductors allowed fewer passengers than before, most minibuses still loaded to full capacity−this made it difficult to maintain social distance. Moreover, most passengers did not use facemasks.

"I have seen that there is no social distance on those buses. But when driving yourself yeah, there is protection because you know you are alone or maybe just the two of you, you mask up" [Immigration Officer, Chirundu border].

Moreover, participants (especially international truck drivers) explained that traveling across the international borders exposed and put them at an increased risk of COVID-19. One driver explained that, within a space of two weeks, he had been to many countries including South Africa, Zimba, Zambia and DR Congo. Participants explained that they spend many days in these countries and on transit−away from home. They explained that, although they sanitise during travel, interaction with a lot of people and handling papers at the in foreign countries, during transit and at the border, put them at an increased risk of the infection.

"I am always moving to Zambia, Congo, South Africa, just like that. I was in Congo and then I connected to South Africa, that same month. I spent somewhere like 2 weeks in South Africa, then I came back" [International truck driver, Chirundu border]

On the contrary, some participants, especially community members and bus drivers, argued that travel did not put them at risk of infection because, before boarding on buses, passengers were made to wear facemasks and that those who did not have were not allowed to board.

Social network

Social networks were also cited as an important factor contributing to the spread of COVID-19 infection in the community. Although the frequency had reduced, people still visited their friends, workmates and relatives, and that their friends. With regard to the timing and frequency of the visits, participants explained that, especially, workmates visited in the evening when they knocked off and that in some instances they visited twice or thrice in a week. In addition, participants confirmed that people still went to the market for their businesses.

For peer review only - http://bmjopen.bmj.com/site/about/guidelines.xhtml

"*If I leave my place I go to her place and if you are to find me, mainly I am there. If I don't go to her place, she will come here, we chat and chat*" ***[Public market trader, Lusaka district].***

On the contrary, some participants explained that they no longer went out to interact with their friends as much as they used to before the COVID pandemic. They mentioned that they had stopped attending church services and stayed at home to avoid contracting and spreading the virus. In addition, some participants explained that, although they visited family members and went to specific places for their businesses, they avoided physical meetings and gatherings; they only communicated on the phone, and that if people went home, they would only greet and not spend together.

"From the time the issues of corona started I have never attended a wedding ceremony or funeral issues, I have never attended" **[Public market trader, *Lusaka district*].**

Discussion

The aim of this study was to explore the psychosocial and cultural factors influencing transmission of COVID-19 in Zambia in order to inform design and implementation of interventions focusing on reducing transmission of the virus in the country. Overall, our findings show that most people were aware of the COVID-19 pandemic and the preventive measures. Nevertheless, several factors including myths and misconceptions and a low risk perception about COVID-19, a negative attitude towards the preventive and control measures, movement and travel patterns, working and living conditions, social gatherings, networks and interactions affect adherence to the preventive and control measures and increase the risk of COVID-19 transmission in the community.

Our findings concerning participant's perspectives about COVID-19 are interesting. They suggest that, people were generally aware of the COVID-19 disease and the associated preventive measures such as social distancing wearing facemasks, washing hands and avoiding overcrowded areas. The Ministry of Health, TV, social media and personal experience seems to be the important sources of information about COVID-19 and preventive measures. Nevertheless, limited access to information on COVID-19 seems to be a major challenge, especially in rural communities where TV reception and internet signal are poor. This finding corroborate a previous study conducted in Egypt and Nigeria [19] which showed that, despite not adhering to preventive measures, most people have satisfactory knowledge about COVID-19 and the preventive and control measures.

Interestingly, our findings suggest that people's attitudes towards the COVID-19 preventive measures affects their adherence to adherence to preventive measures− such as consistent and correct wearing

For peer review only - http://bmjopen.bmj.com/site/about/guidelines.xhtml

of facemasks, social distancing and avoiding overcrowded areas. Further, people's attitude towards the preventive measures seemed to have been influenced by the myths and misconceptions about the existence, source and severity of the disease. The myths and misconceptions appear to have arisen from a lack of information and experience with the new disease. Consequently, participants expressing such beliefs doubted the existence of the virus and the COVID-19 disease and expressed a negative attitude towards the preventive measures. They also expressed a low risk perception towards the severity of and their personal susceptibility to the disease. These findings highlight the importance of attitude and risk perception in influencing people's decision to adopt healthy behaviours− such as COVID-19 preventive measures [22,23]. Our findings suggest that, non-adherence to preventive measures was a major contributing to the spread of the virus in the community. Thus, interventions focusing on improving personalised risk perception, behavioural beliefs and attitude would increase adherence to adoption of a target health behaviour [22].

Life style, living and work conditions were identified to be important factors driving the COVID-19 transmission. For example, the family size and living conditions in most homes−especially in urban high density and rural areas where a number of family members shared sleeping spaces −made it difficult to practice social distancing in the home. Similarly, our findings suggest that sharing offices, overcrowded offices, poor water supply and inadequate sanitisers in places of work posed an important risk factor for the transmission of the virus. Poor water supply affected adherence to hand washing and hygienic practices at home and work; sharing of office space, and overcrowding, especially in markets –made it difficult to observe social distance. These findings corroborate previous studies [25-27]. For example, a systematic review of 68 studies conducted by Zar and colleagues [23] found that poor living conditions in low and middle income countries [LMICs], including lack of adequate sanitatary facilities, running water and overcrowding facilitate transmission of SARS-CoV-2. A study by Ahmad and others [26] reported a higher risk of infection and death from COVID-in counties with poor housing compared to those with modern housing facilities. This evidence is supported by a study in India [26] which reported that overcrowding poses challenges to adherence to social distancing guidelines among urban slum residents.

Moreover, movement and travel patterns within and out of the country were found to be a major risk factor for community transmission and importation of cases into the country, respectively. Although a number of people used private transport for local travel when going for business, work, family matters and other purposes, majority travelled on public transport such as buses and minibuses where adherence to social distance is difficult. Furthermore, although international travel by the general public had reduced at the

For peer review only - http://bmjopen.bmj.com/site/about/guidelines.xhtml

time of the study, international truck drivers still travelled as before, and during travel, most did not adhere to the health guidelines. Interaction with their fellow drivers and other travellers as well as customs and immigration officers all put the drivers at an increased risk of contracting and transmitting the disease. Thus, local and international travel are an important risk factor for the transmission of the virus. These findings corroborate previous studies [15] and local reports [12], which highlighted the importance of international and local travel in influencing transmission and importation of the virus [12]. For example a study [15] conducted in China showed that although travel to foreign countries was responsible for the importation of initial infections into the country, social mixing patterns affected the trajectory of the outbreak within the local community.

Finally, our findings suggest that social networks, frequency and duration of contacts are an important risk factor for the spread of the virus. Despite the government guidelines on restricting social gatherings, most people maintained their social networks through social functions such as wedding ceremonies, church services, kitchen parties, funerals, bars, and home visitations. This finding corroborate previous studies [14, 15, 21] which reported the importance of social gatherings in facilitating spread of the virus and increasing the basic reproductive number. For example, studies conducted in China [14,15] during the early phase of the pandemic which highlighted the effect of social interaction and networks in different age groups and locations on the time-dependent and basic reproduction numbers governing SARS-CoV-2 transmission in the community [15,16]. Thus, policy on regulating local and international travel−especially to high burden countries− and limiting social interactions could help curb transmission of the virus.

Potential limitations of our study should be noted. First, like all qualitative studies, our findings are based on the few IDI participants' perspectives during the first wave of the COVID-19 pandemic when the disease was still new and the number of cases and deaths was still low in the country. It is not clear whether community members' perspectives have changed over the three successive waves of COVID-19 experienced in the country. We could not conduct focus group discussions to compare and confirm the findings due to safety considerations and logistical challenges. Moreover, although Chrindu and Lusaka districts had one of the highest number of COVID-19 cases [15], most study participants had not experienced the disease. The reason for this selection was to have participants who could provide insightful information on the disease, and not necessarily having experienced it. However, the selected participants' perspectives may not have been representative of the views of the other members of the community, especially those who had tested positive to the virus and experienced it.

Despite these limitations, our study provides the first in-depth assessment and analysis of the health workers' and community members' perspectives on the factors influencing transmission of the virus in

For peer review only - http://bmjopen.bmj.com/site/about/guidelines.xhtml

the two districts that were hotspots for COVID-19 hotspots during the first wave in Zambia. Our purposive selection of health workers and community members from both urban and rural sites, with varied socioeconomic backgrounds and experiences with regard to COVID-19, as well as the data collection and analysis conducted by a trained and experienced team minimised bias and increased the internal validity of the study findings. Thus, findings provide important insights into the factors influencing transmission of COVID-19 in Zambia. These factors are important targets for policy and intervention design for prevention of COVID-19 transmission in Zambia and other countries with similar demographic and epidemiological contexts. As far as we know, this is the first qualitative study conducted on this subject in Zambia.

Conclusion

These findings highlight important factors influencing transmission of the coronavirus in Zambia including myths and misconceptions about the disease, politicians' and other important community leaders' conduct, living and working conditions, travel and movement patterns, social gatherings, networks and interactions. Future prevention interventions should focus on: a) increasing people's risk perception and personal susceptibility to the disease, b) improving attitude towards the preventive and control interventions, and d) mitigating structural and socioeconomic barriers in order to increase adoption of and adherence to preventive measures. Finally, further research is needed to measure the levels of adherence to the life-saving preventing and control interventions. Research is also required to test whether adherence to the preventive and control measures actually leads to reduced transmission of the Corona virus and improved health outcomes. Findings from such studies can be the basis for advocacy for a public health policy on these preventive and control measures. This evidence is currently non-existent.

Competing Interest

We declare that as authors we have no conflicting interest in this work.

Authors' Contributions

All authors designed the study. Under the oversight of CS, NM and LL supervised the data collection process. CS and DCS conducted data analysis. CS wrote the first draft of the manuscript. NM and DCS revised the manuscript. FMG advised on the final manuscript. All authors read, commented on and approved the final manuscript.

Funding

For peer review only - http://bmjopen.bmj.com/site/about/guidelines.xhtml

For peer review only

This work was supported by the National Science and Technology Council (grant number NSTC 101/6/8), as part of the Emergency COVID-19 research grant under the strategic research fund.

References

1. Huang C, Wang Y, Li X, Ren L, Zhao J, Hu y et al. 2020. Clinical features of patients infected with 2019 novel coronavirus in Wuhan, China. The Lancet 395 (10223): 497-506

2. Prem K, Liu Y, Russell TW, Kucharski A, Eggo RM, Davis et 2020. The effect of control strategies to reduce social mixing on outcomes of the COVID-19 epidemic in Wuhan, China: a modelling study.
3. World Health Organisation.2020. Coronavirus Disease (COVID-19 Pandemic. Available at: https://www.who.int/emergencies/diseases/novel-coronavirus-2019, accessed on 5th September, 2021.
4. Zhang J, Litvinova M, Wang W, Wang Y, Deng X, Chen X et al. 2020. Evolving epidemiology and transmission dynamics of coronavirus disease 2019 outside Hubei province, China: a descriptive and modelling study. The Lancet Infectious Diseases. Available at: :https://doi.org/10.1016/S1473-3099(20)30230-9, accessed on 6th September, 2021

5. Ksiazek TG, Erdman D, Goldsmith CS, et al. A novel coronavirus associated with severe acute respiratory syndrome. N Engl J Med2003; 348: 1953–66.3

6. Kuiken T, Fouchier RAM, Schutten M, et al. Newly discovered coronavirus as the primary cause of severe acute respiratory syndrome. Lancet 2003; 362: 263–70.4

7. Drosten C, Günther S, Preiser W, et al. Identification of a novel coronavirus in patients with severe acute respiratory syndrome. N Engl J Med 2003; 348: 1967–76

8. World Health Organisation.2020. WHO timeline-COVID-19. Available at https://www.who.int/news-room/detail/27-04-2020-who-timeline---covid-19, accessed on 6th September, 2021
9. Li Q, Guan X, Wu, P, Wang, X., Zhou L, Tong Y et al. 2020. Early transmission dynamics in Wuhan, China, of novel coronavirus-infected pneumonia. *New England Journal of Medicine*. 382:1199-1207
10. Wiley online library.2020. COVID-19 Novel Coronavirus Outbreak. Available at: https://novel-coronavirus.onlinelibrary.wiley.com/, accessed on 6th September, 2021

For peer review only - http://bmjopen.bmj.com/site/about/guidelines.xhtml

11. National Institute for Communicable Diseases. 2020. Available at: https://www.nicd.ac.za/first-case-of-covid-19-coronavirus-reported-in-sa/, accessed on 6th September, 2021

12. Ministry of Health. 2020. Available at: https://web.facebook.com/pg/mohzambia/posts/

13. Ministry of Health. Public Health (Infected Areas)(Coronavirus Disease 2019) Regulation, 2020. Statutory Instrument no.22 of 2020. Available at: https://www.enotices.co.zm/download/statutory-instrument-no-22-of-2020-the-public-health-infected-areas-coronavirus-disease-2019-regulations-pdf/

14. Kucharsk AJ, Russell TW, Diamond C, Liu Y, Edmunds J, Funk S et al. 2020. Early dynamics of transmission and control of COVID-19: a mathematical modelling study. The Lancet Infectious Diseases. 20 (5):553-558

15. Qui Y, Chen X and Shi W. 2020. Impacts of Social and Economic Factors on the Transmission of Coronavirus Disease 2019 (COVID-19) in China. British Medical Journal. doi: https://doi.org/10.1101/2020.03.13.20035238

16. Day M.2020. Covid-19: four fifths of cases are asymptomatic, China figures indicate. British Medical Journal. 369:m1375 doi: 10.1136/bmj.m1375

17. King N and Horrocks C. 2010. Interviews in Qualitative Research. 1st Edition. SAGE Publications ltd. 25-41.

18. Ary D, Jacobs L, Sorensen C and Walker D. Introduction to research in education. 9th edition. Wadsworth, CA: Cengage Learning, 2013

19. Central Statistical Office Zambia. 2010 census of population and housing - Southern Province analytical report. Lusaka: Central Statistical Office Nationalist; 2014. Available at: http://web0.psa.gov.ph/statistics/census/population-and-housing.

20. World Health Organisation, Africa, 2020. Coronavirus (COVID-19). Available, https://www.afro.who.int/health-topics/coronavirus-covid-19, accessed on 20th September, 2021

21. Hager E, Odetokun IA, Bolarinwa O, Zainab A, Okechukwu O, Al-Mustapha AI. Knowledge, attitude, and perceptions towards the 2019 Coronavirus Pandemic: A bi-national survey in Africa. PLoS ONE 15(7), 2020; e0236918. https://doi. org/10.1371/journal.pone.0236918

For peer review only - http://bmjopen.bmj.com/site/about/guidelines.xhtml

22. Cooke R and French DP. How well do the theory of reasoned action and the theory of planned behaviour predict intentions and attendance at screening programmes? A meta-analysis. Psychology & Health, 2008; 23: 745-765.

23. Glanz K, Rimer BK, Viswanath K. Health Behaviour and Health Education: Theory, Research and Practice (4th ed.). San Francisco: Jossey-Bass, 2008; 69-95.

24. Al-Raeei M. The basic reproduction number of the new coronavirus pandemic with mortality for India, the Syrian Arab Republic, the United States, Yemen, China, France, Nigeria and Russia with different rate of cases. Clinical Epidemiology and Global Health, 2020; 9: 147-149

25. Zar HJ, Dawa J, Fischer GB & Castro-Rodriguez JA (2020). Challenges of COVID-19 in children in low- and middle-income countries. Paediatric Respiratory Reviews 35: 70-74 https://doi.org/10.1016/j.prrv.2020.06.016.

26. Ahmad K, Erqou S, Shah N, Nazir U, Morrison AR, et al. (2020) Association of poor housing conditions with COVID-19 incidence and mortality across US counties. PLOS ONE 15(11): e0241327. https://doi.org/10.1371/journal.pone.0241327

27. Wasdani KP & Prasad A (2020) The impossibility of social distancing among the urban poor: the case of an Indian slum in the times of COVID-19, Local Environment, 25:5, 414-418, DOI: 10.1080/13549839.2020.1754375

For peer review only

For peer review only

For peer review only - http://bmjopen.bmj.com/site/about/guidelines.xhtml

BMJ Open

Perspectives on Factors Influencing Transmission of Coronavirus 19 (COVID-19) in Zambia: A qualitative study of Health Workers and Community Members

Journal:	*BMJ Open*
Manuscript ID	bmjopen-2021-057589.R1
Article Type:	Original research
Date Submitted by the Author:	04-Feb-2022

Complete List of Authors:	Sialubanje, Cephas; Levy Mwanawasa Medical University, School of Public Health<br>Sitali, Doreen; University of Zambia, School of Public Health<br>Mukumbuta, Nawa; Levy Mwanawasa Medical University, School of Public Health<br>Liyali, Libonda; Eden umiversity, School of medicine<br>Sumbwa, Phyllis; University of Zambia<br>Kamboyi, Harvey; Eden University, Disease Control and Prevention<br>Ng'andu, Mary; Levy Mwanawasa Medical University, Health Sciences<br>Goma, Fastone; Eden university, School of Medicine

**Primary Subject Heading**:	Infectious diseases
Secondary Subject Heading:	Public health
Keywords:	COVID-19, INFECTIOUS DISEASES, Public health < INFECTIOUS DISEASES, QUALITATIVE RESEARCH

SCHOLARONE™
Manuscripts

I, the Submitting Author has the right to grant and does grant on behalf of all authors of the Work (as defined in the below author licence), an exclusive licence and/or a non-exclusive licence for contributions from authors who are: i) UK Crown employees; ii) where BMJ has agreed a CC-BY licence shall apply, and/or iii) in accordance with the terms applicable for US Federal Government officers or employees acting as part of their official duties; on a worldwide, perpetual, irrevocable, royalty-free basis to BMJ Publishing Group Ltd ("BMJ") its licensees and where the relevant Journal is co-owned by BMJ to the co-owners of the Journal, to publish the Work in this journal and any other BMJ products and to exploit all rights, as set out in our licence.

The Submitting Author accepts and understands that any supply made under these terms is made by BMJ to the Submitting Author unless you are acting as an employee on behalf of your employer or a postgraduate student of an affiliated institution which is paying any applicable article publishing charge ("APC") for Open Access articles. Where the Submitting Author wishes to make the Work available on an Open Access basis (and intends to pay the relevant APC), the terms of reuse of such Open Access shall be governed by a Creative Commons licence – details of these licences and which Creative Commons licence will apply to this Work are set out in our licence referred to above.

Other than as permitted in any relevant BMJ Author's Self Archiving Policies, I confirm this Work has not been accepted for publication elsewhere, is not being considered for publication elsewhere and does not duplicate material already published. I confirm all authors consent to publication of this Work and authorise the granting of this licence.

For peer review only

Perspectives on Factors Influencing Transmission of Coronavirus 19 (COVID-19) in Zambia: A qualitative study of Health Workers and Community Members

Cephas Sialubanje[1*], Doreen C Sitali[2], Nawa Mukumbuta[3], Libonda Liyali[4],Phyllis Ing'utu Sumbwa[5], Harvey Kamboyi[4], Mary Ngandu[1], Fastone M. Goma[4]

[1]Department of Disease Prevention, Levy Mwanawasa Medical University, School of Public Health, P.O. Box 33991, Lusaka, Zambia

[2]Department of Health Promotion, University of Zambia, School of Public Health, P.O. Box 32379, Lusaka, Zambia

[3]Department of Biostatistics and Epidemiology. School of Public Health and Environmental Studies. Levy Mwanawasa Medical University. P. O. Box 33991, Lusaka. Zambia 10101.

[4]Department of Disease Control and Prevention, School of Medicine and Health Sciences, Eden University, P.O. Box 37727, Lusaka, Zambia

[5]Department of Disease Control and Prevention, School of Education, University of Zambia, P.O. Box 32379, Lusaka, Zambia

*Corresponding Author: Cephas Sialubanje; csialubanje@yahoo.com

Mobile: +260 977 441 273

For peer review only - http://bmjopen.bmj.com/site/about/guidelines.xhtml

Abstract

Objective: To explore the health professionals' and community members' perspectives on the factors influencing transmission of the novel coronavirus disease 2019 (COVID-19) in Zambia.

Design: An exploratory qualitative study using in-depth interviews as data collection technique.

Setting: Four primary health care facilities and local communities of Lusaka city and Chirundu international border town under Lusaka province, Zambia.

Participants: Purposive sampling of sixty study participants comprising health professionals (n=15) and community members (n=45). Health staff were health inspectors and surveillance officers. Community members included public market traders, civic and religious leaders, immigration officers, bus and international truck drivers.

Results: Both health professionals and community members were aware of the COVID-19 pandemic, the preventive and control measures. Nevertheless, stark differences were observed on the two groups' perspectives on COVID-19 and the factors influencing its transmission. Most health staff expressed high personal risk and susceptibility to the disease and a positive attitude towards the prevention and control measures. Conversely, myths and misconceptions influenced most community members' perspectives on the disease and their attitude towards the COVID-19 guidelines. Participants were unanimous on the low levels of adherence to the COVID-19 preventive and control measures in the community. Reasons for non-adherence included limited information on COVID-19, negative attitude towards COVID-19 guidelines, social movement and travel patterns, networks and interactions, living and work conditions, water and sanitation facilities, and observation of behaviours of important role models such as politicians and other community leaders. These factors were perceived to increase the risk of COVID-19 transmission.

Conclusion: These findings highlight important factors influencing transmission of COVID-19 in Zambia. Future interventions should focus on providing information to mitigate myths and misconceptions, increasing people's risk perception to the disease, and improving attitude towards the prevention and control interventions and mitigating structural and socioeconomic barriers.

Word count: 294

Key words: COVID-19, transmission, factors, qualitative research, Zambia

For peer review only - http://bmjopen.bmj.com/site/about/guidelines.xhtml

Strengths and Limitations of the study

- The study provides the first in-depth assessment and analysis of the health professionals' and community members' perspectives on the factors influencing transmission of the virus in the two districts that were hotspots for COVID-19 hotspots during the first wave in Zambia
- Our purposive selection of health workers and community members from both urban and rural sites, with varied socioeconomic backgrounds and experiences with regard to COVID-19, as well as data collection and analysis conducted by a trained and experienced team allowed for triangulation, minimised bias and increased the internal validity of the study findings
- The study findings provide important targets for policy and intervention design for prevention of COVID-19 transmission in Zambia and other countries with similar demographic and epidemiological contexts
- The study was conducted during the first COVID-19 wave when the disease was still new and the number of cases and deaths was still low in the country; it is not clear whether community members' perspectives have changed over the three successive waves of COVID-19 experienced in the country.

For peer review only

For peer review only - http://bmjopen.bmj.com/site/about/guidelines.xhtml

Background

Severe Acute Respiratory Syndrome Coronavirus 2 (SARS-CoV-2), a novel coronavirus disease which emerged in Wuhan, Hubei, China in early December 2019 has now spread around the world [1]. The outbreak was reported to the World Health Organisation (WHO) country office on 31st December, 2019 [2-4] which named the new coronavirus disease as Coronavirus Disease of 2019 (COVID-19) [5-7]. Following reports of increased spread and number of confirmed cases and deaths, on 30th January, WHO declared the outbreak a Public Health Emergency of International Concern [7]. With more countries affected, on 11th March, the WHO Director General described COVID-19 as a pandemic [7]. With the disease being reported among health workers and patients with no history of contact with sea food, the mode of transmission of the virus was established as person-to-person through surfaces, droplets and aerosols produced during coughing [8,9]. As of 23rd January, 2022, more than 349 million cases and 5.59 million deaths have been reported globally [10].

The first confirmed cases of COVID-19 in Zambia were reported on 18th March, 2020 and involved a Zambian couple that had travelled and stayed in France for 10 days [11]. Since then, clusters of confirmed cases were reported from Lusaka city and Kafue town. Before long, cases were reported from the Copperbelt province, Nakonde and Chirundu borders with Tanzania and Zimbabwe, respectively, and eventually from all the ten provinces in the country. To date, more than 300 000 cases and 3,900 deaths have been reported in the country [11]. Following reports of the initial cases, the Zambian government enacted statutory instrument number 22 [12] and instituted prevention control measures including social distancing, restriction of social gatherings by closing schools, bars and casinos, restaurants and other businesses; wearing of face masks, washing hands and sanitising. Messages on COVID-19 transmission, prevention and control were disseminated through mass media. Other measures included restriction of international travel as well as closing of international airports and restricting flights to one (Kenneth Kaunda) international airport; screening and testing; contact tracing, quarantine and isolation, and case management. The restrictive measures were intended to reduce the spread of the virus through social mixing and person-to-person contact which promote spread of infectious diseases, including COVID-19 [12].

Social distancing and restriction of social gathering measures have often been used in epidemics [13] because they encourage the general public to avoid crowded places and drastically shift social mixing patterns [13-16]. For example, in their study conducted in China, Qui and colleagues [16] showed that social mixing patterns affect the trajectory of the outbreak within the local community. Social mixing and networks, frequency of contacts in different age groups and locations−such as schools, churches,

For peer review only - http://bmjopen.bmj.com/site/about/guidelines.xhtml

workplaces, households, bars, and other social gatherings−have a direct effect on the time-dependent and basic reproduction numbers facilitating SARS-CoV-2 transmission in the community [17].

Currently, there is limited evidence on the extent to which these interventions are adhered to in the community. Moreover, community members' perspectives and attitude towards these measures are not known. The aim of this study was to explore the health workers' and community members' perspectives on the factors influencing transmission of COVID-19 in Zambia. Information is needed to inform design and implementation of interventions focusing on reducing transmission of the virus in the country.

Methods

Study design

An exploratory phenomenological qualitative study was conducted using in-depth interviews (IDIs) as a data collection technique over a period of twelve weeks, from 3rd August to 30th October, 2020. Use of IDIs allowed for a detailed exploration of the subject under investigation [18, 19].

Study setting

The study was conducted in 4 primary health care facilities and their local communities: 3 from Lusaka city and 1 from Chirundu district under Lusaka province. Lusaka is the capital city of Zambia and has an estimated population of 2.9 million [19]. Chirundu is an international border town between Zambia and Zimbabwe. It is located 115km south-east of Lusaka city and has an estimated population of 127, 600 [20]. Selection of the two sites was done purposively; Lusaka was included in the study because it was an epicenter of the COVID-19 during the first wave. Chirundu town was selected because it was one of the COVID-19 hot spots in the country and it is a town of entry from the bordering countries to the southern African countries of Zimbabwe, Mozambique, Swaziland, Lesotho and South Africa−at the time, South Africa had the highest prevalence of COVID-19 in the region [21]. With regard to health services, Lusaka has 2 tertiary, 5 second level and several primary level health care facilities. In addition, there are private and faith-based health care providers. Chirundu has one faith-based second level hospital and several primary health care level facilities. The Ministry of Health headquarters and the Zambia National Public Health Institute (ZNPHI)−responsible for the COVID-19 policies, guidelines and disease intelligence are also located in Lusaka. Services provided include specialized and primary health care, including health promotion on COVID-19, screening, testing and contact racing, case management and vaccination.

Participants and sampling technique

For peer review only - http://bmjopen.bmj.com/site/about/guidelines.xhtml

A purposive sampling technique was used to select 60 study participants. Purposive sampling allows for selection of participants with similar experiences regarding the health problem under investigation (that is, COVID-19), while, at the same time, allowing for recruitment of participants with different demographic and socioeconomic characteristics−such as place of residence, occupation and income levels. This, in turn, helps provide insight into the similarities and differences in the participants' experiences with regard to the health problem under investigation [18, 19].

Study participants comprised 15 health workers (7 nurses and 8 public health officers) and 45 community members (18 public market traders, 6 bus drivers and 6 international truck drivers, 3 civic leaders, 3 religious leaders, 3 immigration officers, and 6 community members who had recovered from COVID-19). Health workers were sampled from 4 primary health facilities (3 from Lusaka and 1 from Chirundu border town). Eight health professionals worked in the primary health facilities, four worked as port health staff at the Lusaka International Airport; and the remainder worked as surveillance officers at the Zambia National Public Health Institute (ZNPHI). Community members were recruited from the local communities under the four primary health facilities. International truck drivers were selected from Chirundu border; bus drivers were selected from Lusaka intercity bus station. In order to provide insight into the similarities and differences in the IDI participants' perspectives and lived experiences with COVID-19, we sought to balance the number of participants by place of residence and occupation [18, 19].

To be included into the study, participants needed to be:

- Aged above 18 years
- Residing in the selected area for more than 3 months (except for the international truck drivers sampled from Chirundu border)
- Health workers involved in the COVID-19 program from the selected primary health facilities, international airport and ZNPHI
- Bus drivers from Lusaka

Those who were aged less than 18 years and new in the area were not eligible to participate in the study.

Data collection procedures

Four research assistants were trained in interviewing techniques over a period of 5 days. The training comprised two phases: 3 days of theory and 2 days of practical fieldwork. Efforts were made to select research assistants who were skilled and experienced in qualitative research and spoke both English and the local languages, Nyanja, Bemba or Tonga.

For peer review only - http://bmjopen.bmj.com/site/about/guidelines.xhtml

Interviews were conducted at the participant's preferred place, including the place of residence or office. On average, each interview lasted between 1 hour and 1.5hrours. To ensure quality in data collection, a digital voice recorder was used. Each interview was conducted by a pair of research assistants: one facilitated the interview and took notes, the other one was in charge of the digital voice recorder. Before each interview, written informed consent was obtained from each participant; those who could not read or write were asked to mark with an 'X'. To make it easy for the study participants to understand, the consent form (**Supplementary material *1***) was translated into the local language (Tonga and Nyanja). Before the actual interview, each respondent was asked to complete a short demographic questionnaire. Research assistants read the consent form and the questionnaire and filled it in for those who could not read.

Data collection instruments

Data was collected using a paper- based, unstructured interviewed guide (***supplementary material 2***) that was developed by the research team. The interview guide had two pre-determined themes including 1) perspectives on COVID-19, 2) factors affecting transmission of COVID-19. In addition, a short questionnaire (**Supplementary material 3**) was prepared to collect participant demographic and socioeconomic data. To ensure internal validity, both the interview guide and the short questionnaire went through a rigorous development process. First, the principal investigator with vast experience in qualitative research and familiar with the subject, drafted the initial versions. The two themes in the interview guide were adapted from various sources, including the funding opportunity announcement (FOA) from the National Science Technology Council (NSTC) website, the organisation that funded the research, review of the available literature on COVID-19 and researchers' experience in qualitative research methods. Next, the documents were shared with the research team members for their comments and feedback. The documents were revised based on the research team's comments. Two independent bilingual experts translated the documents into the local languages, Tonga and Nyanga. The translated documents were pre-tested during research assistant training and revised accordingly.

Biosafety

To ensure their safety, research team members and study participants were provided with disposable facemasks and hand sanitisers, which they were encouraged to use consistently during training, travel and data collection process. Moreover, research team members were encouraged to observe physical distancing during training and data collection.

Ethical approval

For peer review only - http://bmjopen.bmj.com/site/about/guidelines.xhtml

Ethical approval was obtained from the University of Zambia Biomedical Research Ethics Committee (UNZA BREC, ref 1132-2020); permission to conduct the study was obtained from the National Health Research Authority.

Data processing and analysis

Voice recordings from the IDIs were transcribed and translated into English by two research assistants who were proficient both in English and the local language. To check for accuracy, members of the research team back−translated 10% of the transcripts into the local language and back into English. These versions were then compared for differences and similarities while listening to the original voice recording. After verification of accuracy in transcription and translation, each transcript was then thoroughly read by one research assistant while the other one listened to the corresponding voice recording. Each translated transcript was then compared with the hand-written field notes that the research assistants prepared during the IDIs. After proof-reading and making corrections, the transcripts were saved on a password-protected computer file kept by the PI for safety. The Word documents were then imported into Nvivo 11 MAC for coding and analysis, and the categories and key sub-themes were identified (**supplementary material 4**). Coding was done independently by two research team members experienced in qualitative research analysis. The derived codes and categories were later compiled and compared for differences and similarities. In order to make it easy to compare differences and similarities in the respondent perspectives by group (health worker versus community members) and residential area (urban versus rural), separate analyse were done. An inductive approach was used to analyse the qualitative data in N-Vivo QSR 12, and sub-themes were derived by content-analysis. The inductive approach ensured that sub-themes were derived from the pre-determined themes by content-analysis and grouping all similar statements made with respect to particular themes. Quantitative sociodemographic data was analysed using descriptive statistics.

Patient and public involvement

The study design was determined by the call for research proposals on COVID-19 research under the strategic research fund. Thus, the participants and the public were not directly involved in the conceptualisation and design of the study. However, selection of the primary health facilities and study participants was done in collaboration with the provincial and district health managers. First, an inception meeting was held with the ZNPHI and Ministry of Health staff from the Lusaka Provincial Health Office and the two districts to present and discuss the design and objectives of the study. Next, pre-field meetings were held with the provincial and district managers to select primary health care facilities and local communities to be included in the study. Recruitment of participants (health workers and community

For peer review only - http://bmjopen.bmj.com/site/about/guidelines.xhtml

members) was done by the by the primary health facility managers and local community leaders. Finally, a report was written and shared with key stakeholders, including the funding organisation and Ministry of Health. In addition, a dissemination meeting was held in Lusaka to share the results with the Ministry of Health and community leaders in the districts where the study was conducted.

Results

In this section, we present our study findings in three parts: summary of the participants' demographic characteristics (part one); health workers' and community members' perspectives on COVID-19 (part two); and the factors which influence transmission of COVID-19 (part three).

Demographic characteristics

The majority (60%) of the participants were female, the mean age was just above 38.12 years, and they had, on average, between two and three children. Over half (65%) of the participants were married. One third of the participants (33.3%) had secondary school education, one-fifth (21.7%) had tertiary level education, and 1.67% had never attended school at all. Concerning level of monthly income, 20% had an average income of less than K500 per month. Majority of the sample (51.7%) mentioned having had travelled out of town and 5% out of the country. The countries travelled to were South Africa, Zimbabwe and Demographic Republic of Congo. The detailed demographic and socioeconomic profile of the study participants is shown in table 1 below.

Table 1: Sociodemographic characteristics (n=60)

**Variable**	**Mean (SD) /n (%)**
Age	38.12 (12.08)
Children	2.9 (2.0)
Number at home	5.9 (3.35)
Sex	
Female	36 (60%)
Male	24 (40%)
Marital status	
Single	12 (20%)
Separated	4 (6.67%)
Married	39 (65%)
Widow	5 (8.33%)
District	
Lusaka	36 (60%)
Chirundu	24 (40%)
Group	
Health Worker	15 (25%)
Community member	45 (75%)

For peer review only - http://bmjopen.bmj.com/site/about/guidelines.xhtml

Level of education	
Never attended	1 (1.67%)
Lower primary (1-4)	4 (6.67%
Upper primary (5-7)	12 (20%)
Junior Secondary (8-9)	10 (16.67%)
Senior Secondary (10-12)	20 (33.33%)
College	8 (13.33%)
University	5 (8.33%)
Occupation	
Health worker	15 (25%)
Community member	45 (75.0%)
Level of income	
<K500	12 (20.0%)
K500-999	15 (25.0%)
K1000-1499	5 (8.33%)
K1, 500-1, 999	3 (5.0%)
>K2000	25 (41.67%)
Travelled out of town	
Yes	31 (51.67%)
No	29 (48.33%)
Travelled out of the Country	
Yes	3 (5.0%)
No	57 (95.0%)
Countries travelled to	
South Africa, Zimbabwe, DRC	

Theme 1: Perspectives on COVID-19

In this theme, we present the findings on the health workers' and community members' perspectives on COVID-19 including awareness about the COVID-19, sources of information, myths and misconceptions about the disease and knowledge regarding preventive measures.

Awareness about COVID-19

All the 15 health workers and most (60%) community members (except some bus drivers and public market traders) from both study sites were aware of COVID-19 and explained that they had seen on TV how it had spread to many countries and that people in other countries had died from the disease.

"When you watch on TV you can see the massive deaths which have happened in other countries, it shows that there is COVID" (Community member, Immigration Officer, Chirundu district)

In contrast, some community members, especially public market traders and bus drivers from Lusaka mentioned that, although they had heard about people dying, they did not know whether the deaths were due to COVID-19 or not. They also explained that some people did not believe the disease existed

For peer review only - http://bmjopen.bmj.com/site/about/guidelines.xhtml

because they had not seen it as they did with other diseases like Ebola, and that they would only believe if they saw someone dying from COVID-19. Asked whether they had heard the reports from the Ministry of Health on the number of people who had died in the country, these participants explained that, in Africa COVID-19 did not kill as many people as it did in other continents, and that if COVID-19 really existed in Zambia, most people would have died already.

There is nothing conclusive. I need to see the corpses. Not until I see a dead body of someone and they say this one has died from Covid-19" [Bus driver, Lusaka district].

Sources of information about COVID-19

Both the health workers and community members who had heard about COVID-19 cited the Ministry of Health and the media (radio and TV) as the main sources of information on COVID-19. Especially the health workers, civic leaders and people who had recovered from COVID-19 explained that the information included the mode of transmission of the virus, the preventive and control measures and the need to adhere to the guidelines. By contrast, some participants, especially community members interviewed in Chirundu, explained that it was difficult to access information on COVID-19. They also expressed ignorance with regard to the source of the COVID-19 and the symptoms it causes.

The doctor [Minister of Health] said it on TV. He said that the disease is real and that the number of people with COVID-19 in the country has increased [Community member, Lusaka district]

Myths and misconceptions about COVID-19

Study participants expressed different views on beliefs about the existence, origin and mode of transmission of the disease. Most health workers believed that the disease was real and that it was caused by the virus spread through the air and contact with other people and objects. In contrast, community members, including public market traders and bus drivers expressed myths and misconceptions about the COVID-19. They believed that someone had created the disease in order to eliminate the African population, and that foreigners [Chinese] who were interested in opening mines in African countries, like Congo, brought the disease so that local people could die, and that, eventually, the foreigners would take over the mines. Further, some community members including religious leaders believed that the disease was a mark of the beast recorded in the Bible (Revelation 13). They explained that most people did not believe COVID-19 was real, and that government was playing politics and that politicians were working with foreigners to gain their support and make money for themselves. Some attributed COVID-19 to radiation from cell phones and industries, and that most people died because of radiation from 5G Networks. Consequently, they wondered why they were being asked to test for the disease and to wear facemasks.

For peer review only - http://bmjopen.bmj.com/site/about/guidelines.xhtml

"I am completely confused, is it really a natural disease? I think it is a fabricated disease or something like that. It all came from the Chinse people, and that other people are just inheriting it" (Public market trader, Chirundu district).

Knowledge about COVID-19 Preventive Measures

Health workers and community members expressed contrasting perspectives on their knowledge about preventive and control measures. Most health workers and community members from Lusaka expressed knowledge about the COVID-19 prevention and control guidelines. They explained that they helped disseminate the information in the communities where they worked from and encouraged people to adhere to the guidelines including wearing facemasks, social distancing and avoiding overcrowded places, ensuring hand washing and sanitising. Conversely, most community members including those from Chirundu border, expressed ignorance about the preventive and control measures. They explained that it was difficult to get information about COVID-19 because of the limited access to TV, radio, internet and mobile phone network. Especially bus drivers and public market traders mentioned that they only hear from people in the community and that most information was incorrect.

"They tell us to wear face masks and wash our hands with soap, but most of us do not have anyone to explain to us" (Public Market Trader, Lusaka city)

Theme 2: Factors influencing transmission of COVID-19

In this section, we present the findings on the factors that influence transmission of COVID-19 in the community. They include living and working conditions, travel and social movement patterns, and social networks.

Living and working conditions

Both health workers and community members (i.e., those who believed in the existence of the disease) were unanimous on living and working conditions being important factors driving the COVID-19 transmission. Overcrowding in some homes and work places made it difficult to maintain social distance. Public health officers explained that staff shared the office space and majority did not have enough hand-washing or sanitising facilities; they only used one shared sanitizer and the hand washing facility. They added that some offices and homes did not have running water; where it was present, the supply was erratic and made it difficult for people to follow the guidelines on hand-washing and other hygienic purposes.

"You know our tradition, parents use their own bedroom, then the kids, maybe there are 5 girls, and they use one bedroom. If there are 6 boys they will use one bedroom" [Immigration officer, Chirundu district].

For peer review only - http://bmjopen.bmj.com/site/about/guidelines.xhtml

Attitude towards preventive measures

Participants expressed different attitudes towards the preventive measures. Health workers and some community members (international truck drivers and those who had recovered from or seen someone with COVID-19 expressed) a positive attitude towards preventive measures. They explained that the measures were beneficial to the population. The main benefits were protection against the virus, helping people maintain hygienic standards in the community. They explained that, in the beginning, community members never believed in the reality of the disease until they noticed that even in Zambia, people started dying; some were being quarantined. Health workers explained that there was a need for people to follow the prevention and control guidelines from the Ministry of Health, especially during the cold season, when the risk of transmission of the virus was high.

"What I think is that COVID is there, so the most important thing is to just follow what they say we should do" ***[Public Health Officer, Lusaka district]***

In contrast, most community members expressed a negative attitude towards the COVID-19 preventive measures. They wondered why they were being asked to wear facemasks or avoid mixing with their friends or attending important social functions like church services, weddings or drinking parties. Some participants also explained that it was not clear why even children were told to wash their hands or wear facemasks.

"To be honest people do not like these things, they do not follow because they say, it is a lie, Corona is not there. They are saying because there is no Corona so they do not wear the mask" [Community member, Lusaka district].

Adherence to preventive measures

There was consensus among the participants concerning non-adherence to preventive measures in the community−which was seen as one of the important factors contributing to the spread of COVID-19 in the population. Health workers observed that, although some people followed the guidelines−wore facemasks and used hand-sanitisers− majority did not. They had continued mixing and interacting as before, especially in the markets and buses. Asked why they did not adhere to the guidelines, most community members explained that people in their communities believed that preventive measures came from other countries and local people, especially Africans, were just being told to follow. They also explained that most people were discouraged by what they saw among political leaders who held meetings and political rallies without following the guidelines. They explained that politicians "played politics" as they did not maintain social distance or wear facemasks when holding pre-election political

For peer review only - http://bmjopen.bmj.com/site/about/guidelines.xhtml

rallies. Similarly, most bus drivers confirmed that most passengers did not observe the guidelines when travelling on public transport.

Participants had consensus on the importance of social gatherings such as weddings, kitchen parties, church services, taverns and funerals as virus super spreader events. Nevertheless, they observed that nothing had changed with regard to social mixing patterns; people were used to their old way of life of greeting and hugging their friends when they met. They argued that people did not feel good to stand a meter or an arm apart because of the new rules. Similarly, participants, especially health workers, civic and religious leaders, confirmed that most families had continued visiting and inviting friends or extended family members to their homes for family and social functions such as parties and beer drinking. Although some people did not attend funerals and only provided monetary and other material support, many community members did. Further, shops and churches remained open and people continued going to the market, shops for their businesses, shopping and attending church services. .

"The only time we can say social distance, is if we come to the market, in the shops, or church. There is no social distance when you go to the political rallies" [Religious leader, Lusaka district]

Travel & movement patterns

Both health workers and most community members (except some bus drivers) confirmed that travel within and outside the country was a major factor influencing transmission of the virus. They observed that, despite the COVID-19 situation, local and international travel remained the same. Public transport on buses and minibuses was the commonest mode of transport. Those who owned cars explained that they travelled in their personal cars. Reasons cited for travel were work, business, shopping, family matters (such as funerals), and other purposes.

Bus drivers explained that although they allowed fewer passengers than before, most minibuses still loaded to full capacity−this made it difficult to maintain social distance on buses. Moreover, most passengers did not use facemasks. By contrast, some participants, especially community members and bus drivers, argued that travel did not put them at risk of infection because, before boarding on buses, passengers were made to wear facemasks and that those who did not have were not allowed to board.

"I have seen that there is no social distance on those buses. But when driving yourself yeah, there is protection because you know you are alone or maybe just the two of you, you mask up" [Immigration Officer, Chirundu border].

For peer review only - http://bmjopen.bmj.com/site/about/guidelines.xhtml

International travel was perceived as an important driver of the COVID-19 transmission. Although some countries had closed their borders, participants observed that travel between countries, especially by road, continued. They explained that, international truck drivers spent many days away from home, in foreign countries and on transit. . . One driver explained that, within a space of two weeks, he had been to many countries including South Africa, Zimba, Zambia and DR Congo. Although they sanitise during travel, interaction with a lot of people and handling papers during transit and at the borders, put drivers and other travellers at an increased risk of getting infected or spreading the infection to others

"I am always moving to Zambia, Congo, South Africa, just like that. I was in Congo and then I connected to South Africa, that same month. I spent somewhere like 2 weeks in South Africa, then I came back" [International truck driver, Chirundu border]

Social network

Social networks were also cited as an important factor contributing to the spread of COVID-19 infection in the community. Although the frequency had reduced, people still visited their friends, workmates and relatives, and their friends. They explained that, especially, workmates visited in the evening when they knocked off and that in some instances they visited twice or thrice in a week.

"*If I leave my place I go to her place and if you are to find me, mainly I am there. If I don't go to her place, she will come here, we chat and chat*" ***[Public market trader, Lusaka district].***

Conversely, some participants explained that they no longer went out to interact with their friends as much as they used to before the COVID pandemic. They mentioned that they had stopped attending church services and stayed at home to avoid contracting and spreading the virus. In addition, some participants explained that, although they visited family members and went to specific places for their businesses, they avoided physical meetings and gatherings; they only communicated on the phone, and that if people went home, they would only greet and not spend time together.

"From the time the issues of corona started I have never attended a wedding ceremony or funeral issues, I have never attended" **[Public market trader*, Lusaka district*].**

Discussion

The aim of this study was to explore the health workers' and community members' perspectives on the factors influencing transmission of COVID-19 in Zambia. Overall, our findings suggest that most people were aware of the COVID-19 pandemic and the preventive measures. Nevertheless, several factors including myths and misconceptions and a low risk perception about COVID-19, a negative attitude

For peer review only - http://bmjopen.bmj.com/site/about/guidelines.xhtml

towards the preventive and control measures, movement and travel patterns, working and living conditions, social gatherings, networks and interactions affected adherence to the preventive and control measures and increased the risk of COVID-19 transmission in the community.

Our findings suggest that, people were generally aware of the COVID-19 disease and the associated preventive and control measures. The Ministry of Health, media (TV, radio, internet and social media) and personal experience seem to have been the important sources of information about COVID-19 and preventive measures. This finding corroborate a previous study conducted in Egypt and Nigeria [22] which showed that, despite not adhering to preventive measures, most people have satisfactory knowledge about COVID-19 and the preventive and control measures. Nevertheless, our findings show that limited access to information on COVID-19 appear to be a major challenge, especially in rural communities where TV, radio and mobile phone reception and internet signal are poor. Consequently, a lack of information on COVID-19 seems to have led to widespread myths and misconceptions about the new disease−its existence, origin and severity−and ultimately influenced people's attitude towards prevention and control measures. Further, people's attitude towards the COVID-19 preventive measures seem to affect adherence to preventive and control measures. Participants expressing such strongly held beliefs doubted the existence of the virus and the COVID-19 disease; they expressed a negative attitude towards the preventive measures. They also expressed a low risk perception towards the severity of and their personal susceptibility to the disease. These findings highlight the importance of attitude and risk perception in influencing people's decision to adopt healthy behaviours− such as COVID-19 preventive measures [23, 24]. Our findings suggest that, non-adherence to preventive measures was a major contributing factor to the spread of the virus in the community. Interventions should focusing on preventing and mitigating myths and misconceptions about COVID-19 by providing accessible correct, consistent and timely information on COVID-19 in rural areas− through improved TV, radio reception, and internet and mobile phone connectivity. Interventions should also focus on provision of information about the benefits of adherence to prevention and control measures in order to increase community members' attitude and personalised risk perception. Interventions focusing on improving personalised risk perception, behavioural beliefs and attitude have been shown to increase adoption of and adherence to a target health behaviour [24].

Life style, living and work conditions were identified as important drivers of the COVID-19 transmission. For example, the family size and living conditions in most homes−especially in high density urban neighbourhoods and rural areas where a number of family members shared sleeping spaces −made it difficult to practice social distancing in the home. Similarly, our findings suggest that sharing and

For peer review only - http://bmjopen.bmj.com/site/about/guidelines.xhtml

overcrowded offices, poor water supply and inadequate sanitisers and washing facilities in places of work posed an important risk for the transmission of the virus in the work setup. Moreover, poor water supply, and overcrowding, made it difficult to observe social distance and adherence to hand washing and hygienic practices, especially in public markets. These findings corroborate previous studies [25-27]. For example, a systematic review of 68 studies conducted by Zar and colleagues [26] found that poor living conditions in low and middle income countries [LMICs], including lack of adequate sanitary facilities, running water and overcrowding pose challenges to adherence to prevention and control guidelines among urban slum residents and facilitate transmission of SARS-CoV-2. Further, Ahmad and others [27] reported a higher risk of infection and death from COVID-19 in counties with poor housing compared to those with modern housing facilities. Policies and intervention on COVID-19 prevention would benefit from improved housing, work condition and sanitation and water supply.

Moreover, local and international travel patterns were found to be a major risk factor for community transmission and importation of cases into the country, respectively. Although a number of people used private transport for local travel when going for business, work, family matters and other purposes, majority travelled on public transport such as buses and minibuses where adherence to social distance is difficult. Although international travel by the general public had reduced at the time of the study, travel by road was continued as before. Interaction with their fellow drivers and other travellers as well as customs and immigration officers all put international truck drivers at an increased risk of contracting and transmitting the disease. These findings corroborate previous studies [17, 28] which highlighted the importance of international and local travel in influencing spread of the virus. For example, in their study [28] conducted in the USA, Davis and colleagues reported international travel as the key driver of the introduction of SARS-CoV-2 in the West and East Coast metropolitan areas that could have been seeded as early as late-December, 2019. The study also showed that for most of the continental states the largest contribution of imported infections arrived through domestic travel flows. Similarly, a study conducted in China [17] showed that although travel to foreign countries was responsible for the importation of initial infections into the country, social mixing patterns affected the trajectory of the outbreak within the local community. Our findings highlight the benefits to be gained from interventions focusing on limiting social movement through restriction of local and international travel.

Finally, our findings suggest that social networks, frequency and duration of contacts are an important risk factor for the spread of the virus. Despite the government guidelines on restricting social gatherings, most people maintained their social networks through social functions such as wedding ceremonies, church services, kitchen parties, funerals, casinos and bars, and home visitations. This finding

For peer review only - http://bmjopen.bmj.com/site/about/guidelines.xhtml

corroborate previous studies [17, 29] which reported the importance of social gatherings in facilitating spread of the virus and increasing the basic reproductive number. For example, a study by Alimohamdi and colleagues conducted during the early phase of the pandemic highlighted the effect of social interaction and networks in different age groups and locations on the time-dependent and basic reproduction numbers governing SARS-CoV-2 transmission in the community [29]. The study concluded that reducing the number of contacts within the population is a necessary step to control the epidemic. Thus, policies regulating social interactions could help reduce transmission of the virus.

Limitations and strengths

Limitations of our study should be noted. First, like all qualitative studies, our findings are based on the few IDI participants' views. We could not conduct focus group discussions to compare and confirm the findings due to safety considerations and logistical challenges. Further, the study was conducted during the first wave of the COVID-19 pandemic when the disease was still new and the number of cases and deaths was still low in the country. Zambia has experienced four successive waves. It is not clear whether community members' and health workers' perspectives have changed over these successive waves of COVID-19 in the country. However, the selected participants' perspectives may not have been representative of the views of the other members of the community, especially those who had tested positive to the virus and experienced it. Although Chirundu and Lusaka districts had one of the highest number of COVID-19 cases, most study participants had not experienced the disease. The reason for this selection was to have participants who could provide insightful information on the disease, and not necessarily having experienced it.

Despite these limitations, our study has several strengths. First, the study provides the first in-depth assessment and analysis of the health workers' and community members' perspectives on the factors influencing transmission of the virus in the two districts that were hotspots for COVID-19 during the first wave in Zambia. Second, triangulation through our purposive selection of the study participants comprising health workers and community members from both urban and rural sites, with varied socioeconomic backgrounds and experiences with regard to COVID-19, as well as the use of trained research assistants and rigours data analysis conducted by a trained and experienced team minimised bias and increased the internal validity of the study findings. Finally, our findings provide important insights into the factors influencing transmission of COVID-19 in Zambia can serve as important targets for policy and intervention design for prevention of COVID-19 transmission in Zambia and other countries with similar demographic and epidemiological contexts. As far as we know, this is the first qualitative study conducted on this subject in Zambia.

For peer review only - http://bmjopen.bmj.com/site/about/guidelines.xhtml

Conclusion

These findings highlight important factors influencing transmission of the coronavirus in Zambia including limited information, about COVID-19, myths and misconceptions about the disease, negative attitude towards and non-adherence to prevention and control measures, negative behaviour of the role models such as politicians and other important community leaders, living and working conditions, travel and movement patterns, social gatherings, networks and interactions. Future prevention interventions should focus on: a) increasing access to information about COVID-19; b) increasing people's risk perception and personal susceptibility to the disease, c) improving attitude towards the benefits of preventive and control interventions, d) limiting local and international travel; e) restricting social gatherings, and f) mitigating structural and socioeconomic barriers−by improving housing and work conditions, provision of safe water and sanitary facilities− in order to increase adoption of and adherence to preventive measures.

Finally, further research, in form of survey with a quantitative design, is needed to measure people's attitude towards prevention measures and determine the levels of adherence to the life-saving interventions. Research with a longitudinal design is also required to test whether adherence to the preventive and control measures actually leads to reduced transmission of the Corona virus and improved health outcomes. Findings from such studies can serve as basis for design of public health policy on preventive and control interventions as well as advocacy for adherence to these measures. Currently, this evidence is non-existent.

Competing Interest

We declare that as authors we have no conflicting interest in this work.

Availability of data and materials

Data are available upon reasonable request to the corresponding author and with permission of the UNZABREC Institutional review board.

Acknowledgements

We thank Eden University administration, Dr Flavien N. Bumbangi and Mr Kennedy Chishimba for the logistical support they provided during the training of data collectors and data collection process. Our gratitude also goes to the study participants for their valuable time and input into the study.

Authors' Contributions

For peer review only - http://bmjopen.bmj.com/site/about/guidelines.xhtml

All authors contributed substantially to the development of this manuscript. CS and DCS designed the study. Under the oversight of CS, NM, LL and MN supervised the data collection process. CS and DCS conducted the coding and data analysis. CS wrote the first draft of the manuscript. PIS, NM and DCS reviewed the manuscript. FMG advised on the final manuscript. All authors read, commented on and approved the final manuscript.

Funding

This work was supported by the National Science and Technology Council (grant number NSTC 101/6/8), as part of the Emergency COVID-19 research grant under the strategic research fund.

References

1. Huang C, Wang Y, Li X, Ren L, Zhao J, Hu y et al. 2020. Clinical features of patients infected with 2019 novel coronavirus in Wuhan, China. The Lancet 395 (10223): 497-506

2. Zhang J, Litvinova M, Wang W, Wang Y, Deng X, Chen X et al. 2020. Evolving epidemiology and transmission dynamics of coronavirus disease 2019 outside Hubei province, China: a descriptive and modelling study. The Lancet Infectious Diseases. Available at: :https://doi.org/10.1016/S1473-3099(20)30230-9, accessed on 6th September, 2021

3. Ksiazek TG, Erdman D, Goldsmith CS, et al. A novel coronavirus associated with severe acute respiratory syndrome. N Engl J Med2003; 348: 1953–66.3

4. Kuiken T, Fouchier RAM, Schutten M, et al. Newly discovered coronavirus as the primary cause of severe acute respiratory syndrome. Lancet 2003; 362: 263–70.4

5. World Health Organisation.2020. WHO timeline-COVID-19. Available at https://www.who.int/news-room/detail/27-04-2020-who-timeline---covid-19, accessed on 6th September, 2021

6. Li Q, Guan X, Wu, P, Wang, X., Zhou L, Tong Y et al. 2020. Early transmission dynamics in Wuhan, China, of novel coronavirus-infected pneumonia. *New England Journal of Medicine*. 382:1199-1207

7. Drosten C, Günther S, Preiser W, et al. Identification of a novel coronavirus in patients with severe acute respiratory syndrome. N Engl J Med 2003; 348: 1967–76

For peer review only - http://bmjopen.bmj.com/site/about/guidelines.xhtml

8. Wiley online library.2020. COVID-19 Novel Coronavirus Outbreak. Available at: https://novel-coronavirus.onlinelibrary.wiley.com/, accessed on 6th September, 2021
9. National Institute for Communicable Diseases. 2020. Available at: https://www.nicd.ac.za/first-case-of-covid-19-coronavirus-reported-in-sa/, accessed on 6th September, 2021
10. World Health Organisation.2020. Coronavirus Disease (COVID-19 Pandemic. Available at: https://www.who.int/emergencies/diseases/novel-coronavirus-2019, accessed on 5th September, 2021.
11. Ministry of Health. 2020. Available at: https://web.facebook.com/pg/mohzambia/posts/
12. Ministry of Health. Public Health (Infected Areas)(Coronavirus Disease 2019) Regulation, 2020. Statutory Instrument no.22 of 2020. Available at: https://www.enotices.co.zm/download/statutory-instrument-no-22-of-2020-the-public-health-infected-areas-coronavirus-disease-2019-regulations-pdf/
13. Prem K, Liu Y, Russell TW, Kucharski A, Eggo RM, Davis et 2020. The effect of control strategies to reduce social mixing on outcomes of the COVID-19 epidemic in Wuhan, China: a modelling study.
14. Kucharsk AJ, Russell TW, Diamond C, Liu Y, Edmunds J, Funk S et al. 2020. Early dynamics of transmission and control of COVID-19: a mathematical modelling study. The Lancet Infectious Diseases. 20 (5):553-558
15. Day M.2020. Covid-19: four fifths of cases are asymptomatic, China figures indicate. British Medical Journal. 369:m1375 doi: 10.1136/bmj.m1375
16. Qui Y, Chen X and Shi W. 2020. Impacts of Social and Economic Factors on the Transmission of Coronavirus Disease 2019 (COVID-19) in China. British Medical Journal. doi: https://doi.org/10.1101/2020.03.13.20035238
17. Al-Raeei M. The basic reproduction number of the new coronavirus pandemic with mortality for India, the Syrian Arab Republic, the United States, Yemen, China, France, Nigeria and Russia with different rate of cases. Clinical Epidemiology and Global Health, 2020; 9: 147-149
18. King N and Horrocks C. 2010. Interviews in Qualitative Research. 1st Edition. SAGE Publications ltd. 25-41.
19. Ary D, Jacobs L, Sorensen C and Walker D. Introduction to research in education. 9th edition. Wadsworth, CA: Cengage Learning, 2013
20. Central Statistical Office Zambia. 2010 census of population and housing - Southern Province analytical report. Lusaka: Central Statistical Office Nationalist; 2014. Available at:

For peer review only - http://bmjopen.bmj.com/site/about/guidelines.xhtml

http://web0.psa.gov.ph/statistics/census/population-and-housing.

21. World Health Organisation, Africa, 2020. Coronavirus (COVID-19). Available, https://www.afro.who.int/health-topics/coronavirus-covid-19, accessed on 20th September, 2021

22. Hager E, Odetokun IA, Bolarinwa O, Zainab A, Okechukwu O, Al-Mustapha AI. Knowledge, attitude, and perceptions towards the 2019 Coronavirus Pandemic: A bi-national survey in Africa. PLoS ONE 15(7), 2020; e0236918. https://doi. org/10.1371/journal.pone.0236918

23. Glanz K, Rimer BK, Viswanath K. Health Behaviour and Health Education: Theory, Research and Practice (4th ed.). San Francisco: Jossey-Bass, 2008; 69-95.

24. Cooke R and French DP. How well do the theory of reasoned action and the theory of planned behaviour predict intentions and attendance at screening programmes? A meta-analysis. Psychology & Health, 2008; 23: 745-765.

25. Wasdani KP & Prasad A (2020) The impossibility of social distancing among the urban poor: the case of an Indian slum in the times of COVID-19, Local Environment, 25:5, 414-418, DOI: 10.1080/13549839.2020.1754375

26. Zar HJ, Dawa J, Fischer GB & Castro-Rodriguez JA (2020). Challenges of COVID-19 in children in low- and middle-income countries. Paediatric Respiratory Reviews 35: 70-74 https://doi.org/10.1016/j.prrv.2020.06.016.

27. Ahmad K, Erqou S, Shah N, Nazir U, Morrison AR, et al. (2020) Association of poor housing conditions with COVID-19 incidence and mortality across US counties. PLOS ONE 15(11): e0241327. https://doi.org/10.1371/journal.pone.0241327

28. Davis JT, Chinazzi M, Perra N, Mu K, Piontti APY, Ajelli M et al (2020). Estimating the establishment of local transmission and the cryptic phase of the COVID-19 pandemic in the USA. MedRxiv. 28:2020.07.06.20140285. doi: 10.1101/2020.07.06.20140285. PMID: 32676609; PMCID: PMC7359534.

For peer review only - http://bmjopen.bmj.com/site/about/guidelines.xhtml

29. Alimohamadi Y, Taghdir M, Sepandi M (2020). Estimate of the Basic Reproduction Number for COVID-19: A Systematic Review and Meta-analysis. J Prev Med Public Health; 53 (3):151-157. doi: 10.3961/jpmph.20.076. PMID: 32498136; PMCID: PMC7280807.

For peer review only

INFORMED CONSENT FORM/INFORMATION SHEET

Background

Hello, my name is ___________________________ I am working with the Levy Mwanawasa Medical University and Eden University. This study is funded by the National Science Technology Council.

Purpose

We would like to speak with you because you are ………... We would like to understand your opinion and perspectives on the ***sociocultural dynamics influencing transmission of coronavirus in Zambia***. This research is taking place in Lusaka and Chirundu districts. What you tell us may help the Ministry of Health and government as they try to reduce transmission of ***coronavirus*** among the people who live in this community.

Process

You will be one of approximately 65 respondents asked to participate in this discussion. We would like to ask you some questions in order to understand your opinions and perspectives on the ***sociocultural dynamics influencing transmission of coronavirus.*** The discussion should take no longer than 1.5 hours.

To ensure that I don't miss or forget anything during the discussion, your responses to the various questions will be recorded using a digital recorder. We will not write your name, instead we ask you to sign on this form, but we will keep your signature private. Responses from the various questions will be summarised, analysed and a report written. When we write reports from this discussion, we will not show what you said nor are we going to use your name or signature at any time.

Potential Benefits

You will receive no direct benefit from your participation in this discussion. However, your participation may help the Ministry of Health and the government as they try to reduce transmission of ***coronavirus*** in the country

Risks and Discomforts

The risks of taking part in this study are that other people will hear your responses. It is important that you do not share anything that you are not comfortable with. If you or someone in your family had a bad experience, it may be difficult or uncomfortable to remember or share it. You do not have to respond to any question unless you feel comfortable doing so. You are free to stop the discussion at any time if you need to.
.

Alternatives

You can choose not to take part in this discussion. If you decide not to take part or withdraw from this discussion, you will not suffer any penalty or lose any benefits to which you are entitled.

Participant Costs and Payments

You will not be paid to participate in this discussion. There are no costs to you for participating.

Confidentiality

For peer review only

INFORMED CONSENT FORM/INFORMATION SHEET

Your information will be saved and locked in a computer file and destroyed once it is copied from it. Only study team members will be able to listen to your response. When we write reports from this data, we will not link your signature or identity to your responses.

Participant's Rights

By agreeing to participate in this discussion, you do not waive any of your legal rights. Giving consent means that you have heard or read the information about this discussion and that you agree to participate. You will be given a copy of this form to keep. If you have questions or concerns at any time, you can contact the Principal Investigator, Dr. Sialubanje Cephas on +260-977-441273, the Co-Investigator, Prof Fastone Goma on +260 977 772 301, or any of the staff from the University of Zambia Biomedical Research Ethics Committee on +260-21-1-256067

Right to Refuse or Withdraw

Taking part in this discussion is voluntary. You have the right to refuse to take part. If you decide to be in this discussion, and then change your mind, you can withdraw from it at any time and to skip questions you may deem personal or otherwise without any repercussions. Your participation is voluntary. If you choose to take part, you have the right to stop the discussion at any time.

By signing below you are agreeing to participate in the discussion which indicates that you have read this consent form (or have had it read to you), that your questions have been answered to your satisfaction, and that you voluntarily agree to participate in this research study. You may keep a copy of this for your records.

Signature or thumb print: ____

Date: ____

Signature of Impartial Witness: ____

Date: ____

For peer review only - http://bmjopen.bmj.com/site/about/guidelines.xhtml

INTERVIEW GUIDE

………………………………………………………………………………………………..

<u>Theme 1: Perspectives on COVID-19 preventive measures (social distancing, wearing masks and handwashing)</u>

<u>Q1:</u> Please tell me what you think about the measures government has put in place to prevent transmission of COVID-19

Probe:

- Awareness about COVID-19
- Knowledge about the measures (social distancing, wearing face masks, hand washing)
- Participant's attitude towards these measures (advantages and disadvantages)
- Perspectives on these measures (are the community members adhering to them)

<u>Theme 2: Livelihood and way of life</u>

Q2: Please tell me about your way of life

Probe:

- Family and living conditions (place of residence, number of people at home, number of rooms in the house, condition of the house)
- Source of drinking water (borehole, river, presence of running water)
- Hand sanitising facilities at home
- Occupation/main source of family income
- Working conditions (office space; office shared or not; number of people in the office; use of lifts; toilet facilities, hand sanitising facilities at work)
- Mode of transport to place of work

<u>Theme 3: Travel & movement patterns</u>

Q3: Would you walk me through your recent travel history (past one month)

For peer review only

Probe:

- Movement from home on a typical week day
- Movement from home on a typical weekend day
- Places the respondent visited in the past one month within and outside Zambia
- Duration of stay away from home
- Mode of transport commonly uses/used
- How often the respondent travels/traveled outside home
- The purpose (s) of travel
- People who travel with respondent
- Mode, if any, of protecting oneself against COVID-19 during travel

Theme 4: Social networks

Q4: Walk me through the most important people (family members, friends, relatives, workmates, church mates,) that you have interacted with in the past one month

Probe:

- Most important people frequently in contacts with outside home
- How do you maintain contact with these people (family, friends, workmates, church mates)
- Most recent social activities, meetings, church meetings, weddings, funerals attended in the past one month
- Physical contact with friends & family during social gatherings (i.e. shaking hands, sharing cigarettes/drinks, sharing eating utensils, sharing accommodation/ hotel room)
- Adjustments in physical interaction with family members, friends, workmates (physical/social distancing, working from home, worshipping from home, visits)
- Perspectives on social distancing

THE END

For peer review only

Demographic questionnaire

Questionnaire ID number [ ]

Name of District…………………………………………

---

1. Place of residence (indicate name)………………………………..
2. Number at home (indicate actual number)………………………..
3. Age (Indicate the actual age in years)…………………………
4. Number of children (indicate the actual number)…………………..
5. Marital status (Circle one appropriate answer)…………
   1) Single
   2) Separated
   3) Married
   4) Widow
   5) Cohabiting
6. Religious denomination (indicate appropriate answer)……………..
   1) Catholic
   2) SDA
   3) Pentecostal
   4) Jehovah's witness
   5) Other (specify)…………..
7. Level of education (highest grade reached) (Circle appropriate answer)
   1) Never attended school
   2) 1-4
   3) 5-7
   4) 8-9
   5) 10-12
   6) College
   7) University
8. Occupation (indicate)……………………
9. Level of income per month (in Zambian Kwacha) (Circle appropriate box)………..
   1) <K500
   2) K500-999
   3) 1000-1499
   4) K1,500-1999
   5) >2000
10. Travel out of town in the past one month……..
    1) Yes
    2) No

For peer review only

11. If yes to Q19, mention the place you travelled to (indicate name)…………………..

12. Have you travelled out of the country in the past one month? ……………………

1) Yes
2) No

13. If yes to Q12, mention the place you travelled to (indicate name of place)……………….

14. Mention the date you travelled (indicate the actual date ………………..

15. Mention the date you returned (indicate the actual date)………………..

16. How long did you stay there (indicate actual duration in days)………..

For peer review only

Codebook/Framework matrix

1. Perspectives on COVID-19 preventive and control measures

Participant/Source	: Okay, so you as an individual, as an individual you believe it is there?
IDI 1<br>IMMIGRATION OFFICER	R:<br>Yeah it is there, I believe that it is there.<br>Ahmmmm, when you look at, from the time it was announced that there is Covid and people should start masking up and when you watch on the media. You can see that the massive death which has happened in other countries, it shows that there is uhmmm there is Covid. So we might not know us as layman whether it is Covid or not because in Africa it didn't kill the way in other continents.<br>The measures we are still just following these uhmm social distance, masking, washing out hands and uhmm there are no other measures. We are still getting information from health whatever they will say that the pandemic has extended like maybe in terms of people are dying then maybe they will bring other measures apart from these we are using.
IDI 2<br>TRUCK DRIVER	Completely I am confused, with this type of disease, I am really confused that ahh, is it a really natural disease, or something maybe, I am confused with this disease. It is like it is a manmade disease or something.<br>Yah, someone created the disease for us, so that we can die, because what I can tell you is, in Congo there, there are Chinese people, there, they are opening mines, a lot of mines and the like.<br>So these people they come from there, China to Africa, they are getting interested with, in Africa, there it is congested, their area, there in China, they are congested, they are coming here to open business. So they are, they are, they are trying to, they are sort of appreciating Africa, the way we live in Africa, so they want us to die. So that they can come and take over, that is why they are opening mines and we are always (Unclear speech) Copper, all those things are going to die, those Chinese are the ones, mining here, so those people I suspect that they, they want, they don't like us. When they come then they start to do their business, so such a thing. (Cross Talk)<br>They say it is a Radiation, that radiation is the one killing people, and most of people who died, they died because they have those 5G Networks. That is why mostly, now here, I don't know what is happening, it's like, they can (Unclear Speech), say we have seen that Corona is killing people, to test your people and the like. Then instead of you, of our guardians and the like, to investigate on

For peer review only

	those that they being given by the Chinese, they come and test us and maybe they don't have knowledge about that thing. They are infecting us with corona now and yet we didn't have Corona.<br>Uhhm, we are, we are as we hearing from this World Health Organize that we must have face mask, must have, must keep social distance, avoid going to public places, that's what we are just also doing.<br>**I: Owoo.**<br>R: I don't know how true it is going to work for us anyway, because it is not us who, started those things and they are the ones telling us to do this and, I don't even trust this, so on the same measures.<br>It all come from, all those China people, people are just inheriting, uhmm what term can I use? (Unclear Speech), so I don't think it is fair to us, because we grew up without this disease, and this disease started there in China and those same Chinese, they are the ones coming here again, to say no you must do this, how you truck these people?<br>According to how I am moving they are not taking these measures seriously, you can find that some of the times, they don't do social distance, life as usual they are not taking it serious. See they are not used to these things, more especially Africans, we are not used, these things are coming from there telling us do this, do this so we are trying to (Unclear Speech).<br>Zambians? Zambian I don't have a problem with them, they are trying their best, they are trying their best so that we can ehhhh, put these ehhh, the health measures, they are trying their best. What I don't, what I only uhmm, not happy about Zambia is, them testing us with that thing, go through the nose, I don't trust those things, because, they are given from China, where those things started. They tell you do this, test these people, so that you tell these people and how do you trust these people, maybe they want to kill us, they are going to infect us with Corona, now, yet in old, I didn't know this and I start to get Corona this year. Where did I get it from, where am I going to get it from? So I won't trust again, test me with that stick, I think that stick it is putting me Corona, I don't trust, those things are coming from those same people.<br>Yes, I am not comfortable with it because, if it is a way of, if they can check temperature from me, no problem, inserting something inside me, that is what I am saying, no it is wrong. It is not us who started this and where are they getting these testing kits from?

For peer review only

	(Unclear Speech), they don't insert you something, it is only in Zambia here, wherever you go even Congo, they check your temperature, then measures for preventing Corona, they are there, there is social distance and everything. But that inserting, inside your nose there is no, it is only you here.<br>No, I don't have any problem, the only problem, is inserting something inside me for Corona of which I don't trust, that is something that is going inside me, I don't trust where it came from. If it came from Zambia it is okay, but you still want to go deeper, deeper, you find that it came from China in the process.
IDI 3 TRUCK DRIVER	The disease itself we are just hearing that, it is infecting people, but we don't know how it gets hold of someone, they say what this, the mask if not wearing and found near a person who has the particular disease. Then it is infect them that is what we are hearing it hasn't gotten hold of us before. They don't see it to be serious, they don't, it seems, they don't believe that the disease is there, that is how it seems.<br>Because they are saying those who are drinking beer, them you find that they are not following those rules. You can find that the bottle they are using to drink alcohol is just one, then they are a lot, so if there, there is a disease, it can get hold of anyone that is there.<br>(Long pause). What's this, they are saying it needs people to follow social distance, but still, because of these queues that are found and other things, that disease if there is someone who has it, it can spread. Because of the congestion that's there, from the time Covid-19 came there is congestion, congestion bad.<br>But me I am not found among a lot of people, yes, just the washing hands we are following and wearing these same face masks, also the shield, I have just left it in the vehicle<br>Then we also drink a lot of ginger, I was hearing others on the media in Botswana were saying chew a lot of ginger, put it in hot water, it helps. It behaves as if it is preventing.<br>**I: What about like at home, water?**<br>R:Ehe [/Yes/] even at home they use it.

For peer review only

	At work as well when entering, you wash your hands because the buckets were put, so when going to the offices here you wash your hands, then they don't allow, to enter the yard, starting from the gate, they want face mask.<br>If you don't wear a mask, they won't allow, there.<br>In the bus they don't allow the normal number that it was long ago, the number gets less, so that there should be spaces not sitting near one another.<br>**I: What else?** They put buckets for washing hands and they don't allow to board, without a face mask.<br>**I: In the bus, there is a bucket?**<br>In the same bus yes, because they apprehend the driver at the check point, why have you loaded people without wearing a face mask?<br>**I: In the bus the bucket or what?**<br>In the bus there is a bucket if there is nothing then there is sanitizer. Then also we are found with sanitizer.<br>**I: So from the way you see in the bus, do you think people follow these rules?**<br>No, they don't wear, they don't pay attention, to wearing masks, they are moving anyhow.
IDI 4<br>TRUCK DRIVER	**What do you think about Covid-19**<br>R:<br>It doesn't pass through my place so I don't see it, I don't believe it exists because I don't see it like the way Ebola was. Not until I see a dead body of someone and they say this one has died from Covid-19. Uuuuummmmmhhhhh for now no. Ebola was visible but since I didn't see anyone having it again well it was hard as well.<br>I need the corpse, how can someone just not know anyone not even one who has died from Covid, where I even start from believing. There's nothing that is the conclusion.<br>I know they say wash your hands, uuuummmmmhhh, wear masks every time, even children to wash their hands, sure, sure. Oh yes even social distance but look how we all are in this room, is there even social distance? Aaaaaahhhh this can't just work. Yes I do, can't you seen I am even wearing a mask but I don't like it, I cant breath well you know. They need to put holes for my nose in here. I am covering myself from Covid but I will find myself with another disease due to covering my nose,that is my major challenge with this mask. I even have hand sanitizer here it is.

For peer review only

	I am just waiting for it to finish, I don't need it, wonder when it will finish. But you also need to be asking about food mweh. Otherwise from the time it it started coming, uuuummmmhhhh, uuuuhhhhhmmmm. The measures are okay, we can't blame the government, unless you are not in this world.You know they are the ones ruling us, if they say they are going to kill us they can easily kill and we cant do much. If they say we go to jail we just go to jail. If they decide to put chemicals in our water or in the community we will just live with it. We just have to follow.
IDI 5<br>TRUCK DRIVER	There is nothing I know not even a bit.I just keep hearing that Corona, Corona, but I know nothing. The good part about them is that they are protective, as they say when you do this, you are going to protect yourself, so it is good.<br>(Long Pause) The distancing that they speak of, where is it, Where exactly are you? Is what you need to look at isn't it?<br>So which people am I distancing myself from? The people that I don't know that I am not found with, when I leave this place and I go home, I need to consider that this yard I am entering is my yard. When I bath and wash my clothes, following the old lessons, I look nice. Then you can come and sit and chat with people. So me I can't tell a child that go and sit there no.
IDI 6<br>COMMUNITY MEMBER	Me I agreed, that it are there, on the TV [/Television], the Doctor say it Chilufya, we hear that this is what they are saying. The number has risen, we have known that it is real the disease is there, why have people died?<br>Others what we are using we didn't buy, they distributed, they also gave us masks, like these ones, they said use them for one day don't use them continuous. They just gave us others that are mad of material and said you can use them for 3 days and the 3rd day you wash it if you have one and iron. Then you get another one. They are just okay. Ehhh they are okay, because 1st we don't have to be found dirty and touch each other so they are okay.<br>Even the masks they brought are okay because when you cough, with the things [/Droplets/] cannot get transferred to another person. So like me, I found a little difficulty, for the same mask, when I wear a mask I get a headache. (Long pause) so the way I have won this mask if I wear it too much I get a headache.<br>They follow, (Long Pause) others follow, others are difficult.<br>**I: What about the majority of the people?**<br>R: Majority of the people are difficult, on this very disease, many don't follow.<br>In the community people don't follow the social distance, unless in the shops, and the church they follow.
IDI 7 COMMUNITY MEMBER	And from my own observation being a parent, and a leader at church being a pastors wife, I believe that Covid-19 it is there [/exists/].

IDI 8 MARKETEER	Because if you can see, outside here, the social distance is not maintained and people they are taking it for granted. Corona Virus is serious if you look at people in Italy, if it was in Zambia we would have been finished. If maybe if, if it was in Zambia, could have just remained a few, few people (Unclear Speech)<br>According to me in Zambia Corona virus I doubt it because, because why I say so, people in Zambia they are not taking Corona Virus serious. Ahh on Monday, on 3rd this month I went to Lusaka, I witnessed what the president did, that one it really shocked me, it really shocked me. When I saw the president wasn't maintaining the distance, and the same president, was telling us to say, can you maintain the distance and he himself he failed to do that. Now if Corona it was really there in Zambia, and those minister who they are saying they had Corona Virus, the president himself would have blown, would have been the 1st person to have Corona Virus in the country. Because the president himself failed to maintain social distance. On 3 I have got an evidence, on 3 this week, when he was opening the fly over bridge is Chawama. There were crowd of people, ehh, there are a lot of people in town, no social distance, okay! Then if you go in Lusaka, the same town Lusaka, people social distance, okay! Then if you go in Lusaka, the same town Lusaka, people are not taking it serious, if you look at the transportation services uhmm. You give, the bu-, the bus is not disinfected, and the buss itself it does not have disinfectant they cannot even tell you to wash your hands or disinfect you with sanitizer. Just get into the bus you touch whatever you, what others have touched, and how do you expect to end Corona virus and how do you ahmm how do you convince to say in Zambia we have Corona Virus with such? That's why sometimes I doubt if you have, if we have Corona Virus in Zambia. If you really had Corona Virus in Zambia, we would have finished already<br>Have you really tested to say, people are really dying with Corona Virus? Or they are just dying of other diseases or people are just accusing to say this is Corona because has died in a period of Corona.<br>Okay apart from the government telling us to maintain social distance and putting on face masks, what is it that the government of Zambia has done, toward the Corona Virus to its own people. What measure did they do, or what measures are there government themselves, okay, what measure did the government implemented? to the people of the nation. Apart from telling us to put on a face mask to maintain social distance, to avoid touching, what measure did they do to us?

For peer review only

	But we need to know madam we need to know, okay. You and I, we have got the right to ask, such kinds of issues eh, In Zambia there is a Pandemic, Corona Virus, now what is the government doing, did they give the free face masks? To you as an individual They are not supposed to take In the hospitals, there are people in the market here. They don't do that, did they give free hand sanitizer to people.
IDI 9 marketeer	(Long Pause) I know even know how to really say it. Us we are just following what the government is saying that do this, do that, but is it serious this same one, like the true. I don't know anyway.<br>They are saying it is there.<br>**I: What of you?**<br>(Laughs) it's there, I don't know, I am not seeing it. In other countries.<br>I really don't know, they keep telling us, wash your hands, the disease is in the hands. Don't know if it greeting each other that brings the disease or its touching s Ehem unless you are going elsewhere, okay this disease is not known, it is not seen. It is not visible that here it is there, here it isn't, we are just living like I don't know. You should just follow the laws, to protect yourself.<br>tuff, we don't know, you just waking up and washing hands, maybe the disease is in the medicine, we don't know. Hummm, (Sigh) its okay, just like that it is to follow, because you can't know.<br>Hmmm (Laughs), they are just okay because we are just washing hands, okay here in Chirundu we have not yet just seen.<br>Uhmmmm, like here, like here they are not taking it seriously, the way I am seeing it. They are taking it but, masks, it is often for them to wear. Just as you have seen us, just like this<br>People are not following, (Unclear Speech) we just do them, maybe you have started off, there is somewhere you are going, maybe it is Lusaka. (Unclear speech) sanitizer in the hands, you are paying attention, now, coming here, here they have taken it as it is not there [/doesn't exist/].<br>Social distance is what is not there, but the masks they are wearing, on the road blocks, once you pass, all this side you be moving like that when you reach Monze you put on.

	No, a lot of people are not following, I tell you, they are just dibili, dibili, moving anyhow at the end that is when they remember that there is Corona, when they bury.<br>People haven't believe that the disease is there, they don't take it serious.
IDI 10<br>TAILOR	Covid-19, ahhmmmm I see it that it there it exist, I used to think they are lying<br>I never used to think it is true, us people just how we used to behave, but, then I noticed that even in Zambia, people started dying, being quarantined. Especially now when it was cold so what we need to do is to, follow the guidelines that the ministry of health had put up, social distance, washing hands, wearing masks. Especially me who is sowing, because most of the time you find that the truck drivers, are in town moving about the market. So it not safe for us.<br>No, they don't follow, unless the DC [/District Commissioner/] passes through that is when they run to buy masks. If the Dc is not there, even if you go round you will find 3 quarters of the people are not in masks (Tailoring Machine Noise)<br>I don't know if at all, people don't believe that Covid is truly there. If the DC didn't pass through, nothing, nothing, but if the DC passes through, they will be all over fast rushing to buy masks. Sometimes I even make k400 here just selling masks.<br>Not even one bit, for example, look at the butchery, there is a bucket outside with water, but none have even passed to even consider washing their hands. So me<br>To be honest they don't follow because they say, it is a lie, Corona isn't there.<br>They are saying because there is no Corona so they don't wear the mask.
IDI 11 MARKETEER	The question I can ask is, this disease of Corona, in what way did it come, we just heard it like Vapour like ey, there is Corona, Corona, Corona?<br>**I: Uhmm.**<br>Uhmm, even the way one gets sick, me I have never seen it nicely, is it a cough, or a cough (Baby Crying), because a cough, in all days, people get sick of a Cough, Malaria, Headache. So now this disease of Corona how is it like?<br>I follow but not very much.<br>**I: What of here where you work?**<br>R:<br>Ahhh, here we don't follow them very much.<br>**I: Okay.**

For peer review only - http://bmjopen.bmj.com/site/about/guidelines.xhtml

	R: Uhmmm don't know when we go in the main market.<br>In Lusaka, I noticed a lot of people wear mask, but here in Chirundu, here in Chirundu many don't wear, but in Lusaka many wear. Even in the bus I got into in Lusaka, everyone, starting with the driver.<br>Uhmm, even the way one gets sick, me I have never seen it nicely, is it a cough, or a cough (Baby Crying), because a cough, in all days, people get sick of a Cough, Malaria, Headache. So now this disease of Corona how is it like?
IDI 12	Uhmmm then washing hands and Social distance. Then the mask, sometime you find people complaining that they are not breathing well in wearing those masks. This makes it difficult for people to wear the masks.<br>**I: Alright you meaning breathing?**<br>Yes, sometimes the way I have sat here right, you find that, I am feeling like the air is going to finish [/suffocating/]. You do this or that, so, a lot of times many people don't wear because they are failing to breath. Just like the way people sleep in mosquito nets.<br>**I: Uhmmm**<br>Others don't sleep in mosquito nets, why, they say that they can't breath properly [/Suffocating/]. Same with the masks other people suffocate. But the biggest thing they just have to continue sensitize people. Teaching them, the importance of a mask. The other thing they are saying social distance yeah. Social distance like in the market, it is not possible, why because in the market the people are a lot. We are way too many in the market, at least here we can say social distance but look at there where the other people are. It is not very much in the mind that people should maintain social distance, so this issue of social distance, it ahhhhh, I can't see it. Cause even these political parties if you see them, where they are, when they are doing campaigns I don't see social distance. Social distance we are only following it when we go to church, that is the only time we can say social distance, otherwise if we come here to the market, there is no social distance, when you go to the political rallies there isn't social distance, that is why this disease is continuing why, Because people are not following the rules. So the biggest issue is that they continue teaching, or maybe there is, another way in which they can do it that they can help so that this diseases doesn't keep on being transmitted. Otherwise social distance it, we are far from it

For peer review only

	It is difficult, just like, okay like us who are married yeah, my husband will go out of town, then the government says social distance. How can I social distance with my husband? Is it possible? That one it can't work, yes at church we are managing to follow social distance but in the houses, there is no social distance, there just has to be other measures than social distance.<br>Because where I sit, I am at the, door, I see other don't have sanitizer, others don't wash hands, just like that. So I can see that us people don't follow these measures in the community, otherwise, these measures are only followed at church. Because, I think at church there is a spirituality, since it is church, when the usher arrives they tell you wash your hands. Sanitize, but here, it is I don't care type each one with their own. You see, if w used to follow the way we follow at church, if that is what we do in our homes and the market, it could have been very okay.<br>Hmmmm, sometimes, some forget, others it's that attitude that there is no Covid. You tell them you should be washing hands, they say its not there.<br>So they say, there is no Covid-19, they are just politics. That is what others say. That is why they don't follow.<br>**I: However so for others, its just negligence?**<br>R:<br>Others do it on purpose, others it's forgetting, others just don't want.<br>Hmmmm like what I was telling you, the one 1 meters are not manageable. Us who are here outside you have seen how we are seated? At least these ones can maintain, but when you look inside the market and look inside, it is not possible, the 1 meter is not manageable. The space is too little because we are too many.<br>Just like I told you in the beginning that at church there is no problem, when it comes to following those rules. Each time when going to church, each person tends to adhere to those rules.
IDI 13 MARKETEER	What I think on my part is Covid is there, so the biggest thing is to just follow what they say we should do
IDI 14 MARKETEER	Covid-19, ahhmmmm I see it that it there it exist, I used to think they are lying<br>**I: Why do you think that way?**<br>I never used to think it is true, us people just how we used to behave, but, then I noticed that even in Zambia, people started dying, being quarantined. No, they don't follow, unless the DC [/District Commissioner/] passes through that is when they run to buy

For peer review only

	masks. If the Dc is not there, even if you go round you will find 3 quarters of the people are not in masks (Tailoring Machine Noise)<br>**I: So why do you think the people don't follow these measures?**<br>Ah, I don't know, maybe I am not knowledgeable but I really don't know. Maybe they are not aware.<br>I don't know if at all, people don't believe that Covid is truly there. If the DC didn't pass through, nothing, nothing, but if the DC passes through, they will be all over fast rushing to buy masks. Sometimes I even make k400 here just selling masks.<br>To be honest they don't follow because they say, it is a lie, Corona isn't there.<br>**I: So if there is no Corona, they don't want to wash?**<br>They don't want to wash, or even just wearing a mask they don't want.<br>**I: Why do you think they don't like to wear the mask?**<br>They are saying because there is no Corona so they don't wear the mask.<br>I still think it is real.<br>**I: You still think it is airborne?**<br>R:<br>No, (Tailoring Machine Noise) Corona is there definitely.
IDI 15 marketeer	Yes, I have heard that it is a Virus. It comes with the wind you cant see it, so I think that it is the air.<br>What I think on my part is Covid is there, so the biggest thing is to just follow what they say we should do.<br>Ahhhh, where I go, people, you know the disease is new so a lot of people have not yet gotten used to it. They don't know that Covid is like this, like that. But a lot are not used. Sometimes many just stay that way, sometimes, others know that it is bad and they follow the guidelines. They wash their hands, they don't sneeze near people, they don't sit near other people, just sit alone, just like that.<br>**I: So do they manage? The whole community.**<br>R:<br>Uhmmm it is not everyone who manages, others manage others don't. they just know Covid is there, you need to wear a mask, wash your hands, but others who know, are taking care just like that [/Customer interruption, 4 minutes of the interview/].

For peer review only

	Ah, now those things is there even so much money, but anyway others don't manage but on my part there is nothing I can't afford not to buy.<br>**I: So why do you think others don't manage?**<br>R:<br>Well others its that situation where they don't believe that Covid is there and others believe that Covid is there that is why they follow. Because if you know that the disease is there then, you just have to uhmm, sanitizer isn't expensive, mask isn't expensive, water isn't expensive. So if you have all these things and you are using them everyday, anytime you are using them it isn't something that can bring a loss to you. When you buy sanitizer it won't finish right now, it will stay even for 2 weeks. Mask if you want, you can make a Chitenge kind. Ehe, anytime, often you wash it, there isn't anything expensive that the person cant fail, people can manage. Just that people are not taking it seriously.<br>**I: Okay.**<br>R:<br>If they were taking it seriously, everyone would have followed.
**SUMMARIES**	There has been general unbelief that COVID-19 is real .Some believe that COVID-19 is real, because they saw how people in other countries died, and others because government has said so. There is still unbelief in many people for the following reasons; the disease was brought in by Chinese who are now trying to infect Africans through testing kits. On the other hand, some participants feel that COVID-19 is not real because they have not seen anybody die from the disease, because politicians are not observing preventive measures themselves, that government is playing politics, while others think it is caused by technology . Most people were not practicing preventive measures especially social distancing.prevention only attempted mostly during church meetings
**2. Livelihood and way of life**	
**Resource**	
IDI 1 IMMIGRATION OFFICER	It is supposed to come out like immigration officer or?<br>Within the Range of k5000 Where we get out water, we get tap water, we are using tap water, so unless there are other issues you want to know about.

For peer review only

	Ummm yes we live in a 3 bedroomed house.<br>Uhmmm Yaaaaah, we have our own rooms but as a family you know you cannot have your own single room. It means in each room maybe if there are babies, it means you know our tradition, the parents they are using their own bedroom then the kids, they are 5 girls, they will be using 1 bedroom. If there are 6 boys they will be using 1 bedroom, but for me I think I am okay, the bed space is okay, because we are only 4.<br>**I: You are 4 and 3 bedrooms, so each one has?**<br>R:<br>Yes, except one bedroom is being used by 2<br>Uhmmm normally I don't have those you are using in Public and in the offices, I only have the sanitizer and the hand wash. So normally we are using most of the time the soap.<br>Yes, because where there is hand sanitizer and the, the hand soap, there is no basin for washing. So everyone they option to go to the bathing room and wash their hands.<br>I ammm, I ammm employed as a public servant.<br>At times I walk, at times I drive<br>When travelling it's just masking, but if it is public transport, they are talking of social distance, but I have seen there is no social distance on those buses. But when driving yourself yah, there protection because you know you are alone or maybe just the two of you, you mask up.<br>Uhmmmm, Uhmmm we are maintaining social distance and masking up.<br>Yah its, uhmmm it's a bit uhmmm, you don't feel good when you meet a friend maybe after a long time we are used to hugging and this time even if you your friend who you saw some time back you just stand a meter apart or hour. You maintain social distance, so anyway I, we are getting used, because of late, I have been seeing people have stopped shaking hands. So we are getting used.
IDI 2 TRUCK DRIVER	I stay in Harare, Zimbabwe.Harare in Zimbabwe.

For peer review only

	**I: (Long Pause). So at home, how many people do you stay with?**<br>I stay, with, my wife and 2 kids.<br><br>**I: So you are 4 you?**<br>Right now, yes we are 4, 4 of us.<br>Average? Average per month myself I am earning something like 700 U.S [/U.S dollars/], I earn in U.S dollars. Our company they thought it is better to pay us in U.S dollar because we are working in U.S dollars, so it was fair for it to us the company.<br>There is nothing we can do because, found me working already as a traveler as a truck driver (Unclear Speech) so there is no way I can't, because Covid came, I can't quit my job, how am I going to feed my family?<br>We try by all means, and we sanitize, right now I come here, I touch all these paper eh? When I reach here I find my sanitizer in my truck, when I reach my truck, I sanitizer (Unclear Speech) I sanitizer, because I am coming from a public place. So that is how we are doing it, we are just following those things, whatever you are doing, we must do this so that you don't, you don't uhm infect other people.<br>Me I have got a 7 roomed house.<br><br>**I: And you are just the 4 of you in the house?**<br>No one lodging in my house, its only 4 of us, the other one, my son, he is working, staying, where he is staying with his wife, he came he went out of my house. The other daughter of mine, is also at the university and is always at school. So we are just 4 of us, each one sleeps in his room.<br><br>Ahhh (Unclear speech) obvious when we go out everyone is wearing a mask, but we avoid going to Public places, like going into a supermarket with my wife and my kids, most of the time, I bring the groceries from South Africa. What they go to buy is maybe bread (Unclear Speech) They don't go to public places, like the supermarket, its only me who goes to public places, that is the nature of my job, I cannot stay<br>Yes, they are out, they are married, 2 of them are married are already married girls and the other son is married also he is working and staying (Unclear Speech).<br>From the bore hole.<br>Within ahh, within my place (Unclear speech).

	**I: So ahhmm you Sanitizing and taking the measures, do you have running water?**<br>R:<br>Yes, the toilet it has got running water, I have water I have got a borehole (Unclear speech).<br>Visitors, they do come but in my house, rarely, very rarely.<br>(Unclear Speech), obviously you will be walking with a mask, we don't shake hands, like any of those days that we used to, all those things, like social distance. That's what we are doing we are trying our best anyway.<br>Yah me I am found home, total maybe 2 days in a month.<br>Home, home myself I don't go anywhere, I am always away at home, so that is the time for me to be at home. I am always away, like 1 month I am away from home, when I reach home, I have to be at home. So that I get familiar of my children and whatever is happening at my home, even beer, like I go buy my beer I drink at home.<br>That's what I do, I don't go to drink beer like to public places, I drink from home, I am an old man.<br>**I: So if it comes to spending your weekend, how do you spend your weekends if they find you home?**<br>Home, if my weekend find me home I am drinking beer with my wife.<br>It won't be a party if we invite other people, so it's only for my family, so that they know that daddy is around, I buy them meats, we braai and even beer we are chatting there, that's all I spend 2 days and off I go.<br>There won't be anyone, just buy things off you go, you go on the window, so that there won't be anyone (Unclear Speech) but we don't go inside the window<br>No, you stay in the truck because most of the time I will be offloading, you need to offload, I finish, I break and you need to load.<br>So for me to go to town, I will be so busy for me to go to town I don't think I can't even manage.<br>I stay in my truck most of the time, but now in my countries like this, I get acquitted like this, I eeeee I am just 1, I drive off I go to Congo, then I go to Kasumba lesa, I don't meet anyone because I am closed in my truck. Problem when you get to boarders like this you have to go out, and (Unclear Speech) otherwise if it wasn't for this formality, I would be in my truck cooking.<br>R Uhmm most of the time I chat with my friends, the drivers, and ehh uhmm, most of the time, (Unclear Speech) we will be together with my friend, because those are the ones that we chat with. The drivers mostly, and query agents that's all

	I go to my truck, but people, but people sometimes I talk to my fellow drivers, those are the ones that I (Unclear speech) don't do that.<br>I am always in the truck the only funeral that happened, I assist with money since I wont be there, or maybe, my wife sometimes also goes there, that's all. Me I won't be there because sometimes a funeral happens maybe when I am in Congo, for me to go fast to attend that funeral (Unclear Speech) I will find that person buried. Then I will only maybe go to say my condolences, what what, you call it, that's all.
IDI 3 TRUCK DRIVER	I stay in Ndola, in Masala.<br>**I: Masala in Ndola?**<br>Hmmm.<br>**I: At home how many do you stay? All of you in total in your house?**<br>We stay 5, 2 children, then 1 In-law and my wife together with me.<br>**I: Okay 5. How old are you?**<br>29.<br>**I: (Long pause). Which church do you go to?**<br>Uhmmm, at the United Church of Zambia.<br>I am a driver.<br>Uhmmm it gets to fluctuate because we work for the Chinese so sometimes you get maybe 3,5 [/k3,500/], 3, 7 [/k3700] or 4000 [/k4000] but most times it is 3, 5, [/k3500] 3,9 [/3900/].<br>Mmmmm because most times, the trips that we make are for transit so in Ndola, if you stay too much its 1 week, then you have exceeded, because when you leave Congo you have to proceed up to South Africa.<br>But me I am not found among a lot of people, yes, just the washing hands we are following and wearing these same face masks, also the shield, I have just left it in the vehicle.<br>Then we also drink a lot of ginger, I was hearing others on the media in Botswana were saying chew a lot of ginger, put it in hot water, it helps. It behaves as if it is preventing.

For peer review only

	**I: What about like at home, water?**<br>R:<br>Ehe [/Yes/] even at home they use it.<br>**I: Where do you fetch water?**<br>R:<br>Water there is water for the council, because those houses, they put what's this, the taps, only that the way in which we fetch people are lot. You find that 1 Tap houses relying on it are 4, again another Tap 4.<br>At home we don't allow Children, they are found at the yard and the house, and those people who wash their hands, as in washing their hands.<br>That company is in Kabwe then me I stay in Ndola, when I leave the vehicle there, I get to board a bus taking me to Ndola then following day again, or that other day, that is when I start off back to come to Kabwe.<br>**I: Hmmm. You get on the public bus?**<br>R:<br>The same big public buses the big ones.<br>If it is drinking, just drink from home itself.<br>**I: So when you leave home, going for work, how long do you spend away from home?**<br>R:<br>Away from home, uhmmm it takes time, because, if you go to Congo, you stay 2 weeks or 3 weeks, because of the process for papers they take long.<br>2 weeks just, those are the days that we spend when there.<br>**I: So your movements, whom do you move with? Do you move with friends?**<br>R:<br>Uhm Uhm I am found alone, because they don't allow.<br>**I: what if it so happens that you visit your relatives?**

For peer review only

	We visit but not so often.<br>**I: So when visiting, how?**<br>When we are visiting well not everyone wear a mask, but us we wear a mask when chatting since they say through the spits of Saliva. That is what we are hearing.
IDI 4 TRUCK DRIVER	I am 38 years old.<br>**I: How many children do you have?**<br>I have 4 children.<br>**I: What is your marital status? Are you Married?**<br>: No my wife died, so put widow there.<br>**I: Okay, I will indicate widower underneath. What do you do for a living?**<br>I am a truck Driver.<br>**I: How Much money would you estimate to make monthly or if it is fixed then how much?**<br>Roughly I am paid k1300.<br>**I: How many rooms are in the house? How many are you in the house?**<br>We are 7 in the house, 6 rooms 3 bedrooms, I alone in one bedroom my wife died during the birth of our fourth baby during an operation, then one is bedroom empty, the others 6 of them sleep in one room altogether.<br>I am 38 years old.<br>**I: How many children do you have?**<br>R: I have 4 children.<br>**I: What is your marital status? Are you Married?**<br>R: No my wife died, so put widow there.<br>**I: Okay, I will indicate widower underneath. What do you do for a living?**<br>R: I am a truck Driver.<br>**I: How Much money would you estimate to make monthly or if it is fixed then how much?**<br>Roughly I am paid k1300.

I: How many rooms are in the house? How many are you in the house?

We are 7 in the house, 6 rooms 3 bedrooms, I alone in one bedroom my wife died during the birth of our fourth baby during an operation, then one is bedroom empty, the others 6 of them sleep in one room altogether. Yes I do, can't you seen I am even wearing a mask but I don't like it, I cant breath well you know. They need to put holes for my nose in here. We are 3 houses in one fence, 6 rooms, 3 bedrooms, I sleep alone, then my children and other all 6 sleep in one bedroom. I have a baby lives with my mother in law. My wife died maternal death during cesarean section leaving the baby behind. So the baby lives with my mother in law my late wife's mother. Its okay it was early last year, baby still small, my sister takes care of the kids in the house.

I: It is well, all is well, what about the water where do you get your water from?

Get if from the Tap there is a Tap but it goes a lot so it may come around 01 and just store in containers and buckets.

I bought everyone at home hand sanitizer.

I am always alone. I go with my personal vehicle to Kabwe that's where there is alone, pack it get the truck and I begin to travel always alone.

Every month I travel out I take good there mainly like I said Congo Zambia, South Africa. I even wear the mask as you can see because of the police not because I believe it is there but they may keep stopping me due to not wearing one. I can't breath in this thing I really need them to make holes. Besides the Same China that brought Corona is the one bringing the masks they could be coming with Covid.

Well, I don't have friends so there are no visitors at my home ever and never home I am working for 2 weeks am out I just go sleep maybe for one night and then proceed. The only friends I have are these same workmates, the truck drivers am in contact with. Then sometimes I can go with them but maybe their papers are not ready so I leave them and proceed. Even them if my papers are not ready they will leave me behind just like that life goes on. When I am in Zambia on Sunday, I go to church but honestly I haven't been to church in 3 months so far. I still do handshakes am not a Ja Man, honestly am not a Ja Man, I don't ever follow those social distance rules myself, so it is okay for me this way when it comes to Covid-19.

For peer review only

IDI 5 TRUCK DRIVER	Kapiri.<br><br>**I: Kapiri, then altogether at the house you live how many are you?**<br>R:<br><br>Altogether 7.<br><br>**I: 7, then how old are you?**<br>R:<br><br>39.<br><br>I am a Jehovah's Witness.<br><br>**I: Okay, Then at school what grade did you end.**<br>R:<br><br>I have never been to school<br>I can't honestly count.<br><br>**I: You know sometimes, you can know that you make 5 pin [/k5000], 10 pin [/K10,000/] even it is 1 pin [k1000].**<br>**R:**<br><br>Put even a 10, 000 [/K10,000/].<br>No me at home I just sleep, 2 nights.<br><br>We sit like 2 or 3, one sits here, another there. Then you chat.<br><br>On my part when I return I go and wash my hands and sanitize but that sanitizer to be honest I just like water and wash, I get in.<br><br>Water, is for the Council, the who, the who and Sewage, the Luvanga. Bedrooms are 3.<br><br>**I: You said 3 bedrooms, water you said you use water and sewage?**<br>R:<br><br>Hmmm.<br><br>**I: Are they taps?**<br>R:

For peer review only

	They are taps, they don't bring, water doesn't run throughout, comes once a month.<br>Ehh [/Yes/], once you draw twice, then when does it come, what I don't see means it doesn't come.<br>**I: So when you don't have water where do you fetch from?**<br>R:<br>Company provided face masks, sanitizer, everything they give equivalent to the journey that you will take. So I think even them they follow the guidelines isn't it?<br>People, we are a lot, but how we are going to move, how many of us will go, on my part we move the 2 of us.<br>**I: In the Vehicle there are 2 people?**<br>R:<br>Vehicles are 2, each vehicle with one person that is all.<br>We pour water on each other [/Help each other wash/], washing when eating or what? Because how we work, this issue of washing hands so that you protect yourself, each person has bottles of sanitizer, for cleaning hands. Just like this, you leave here, you wash the hands and clean.<br>Because where we are found it is not all the time that you find water every time.<br>**I: So when going to work what transport do you use?**<br>R:<br>I use a vehicle<br>On that part, doing what now? Now I am the owner of the house, the father of the house.<br>**I: You are just at home or you get to leave?**<br>R:<br>Sometimes I am found at home. Other times, I go to the farm because that is where my heart is.<br>The problem I have I don't know if it is dullness, I don't have friends, friends that I have are my children and my wife.<br>**I: So you don't have friends in the neighborhood?**<br>R:

For peer review only

	No, I don't like gossiping.<br>**I: Do you drink alcohol?**<br>R:<br>Alcohol I drink a lot if you buy for me, you will see how I will treat it.<br>**I: So whom do you drink with?**<br>R:<br>At home, from a long time ago if I feel like drinking, its at home.<br>Living is on my personal part that is what I am saying it is the same I am here, my truck is my home, it is my office. So when I come it is 10 days which includes transit. I arrive today I offload there, I reach again I offload. Depending on the time, I go and pack when I arrive I register and wait, if I am given the papers the next day I proceed.<br>So it is not specific that I will stay 2 days, 3 days, to even see that there this is how they live<br>Corona came, but he declared it years ago from back days, there will be dangerous diseases, there will things of this nature. So before the leader even spoke of how to protect yourself, when they see they are quick to say, gathering in numbers stops. So in order to associate together all of us we use Zoom App. So we get into Zoom you looking at each person and speak about all the issues. If I am talking with you I get numbers we get to talk on the Apps or telephone<br>R:<br>I have never gone even my mother in Law passed on when I was in South Africa.<br>Social distance is something that is difficult, because in our Tradition it is not there, so for it to be used, it is difficult to accept because it is new so it is something is difficult.<br>In my place, us, as people of Africa, being a person we are used to being together, and working together, so to break that tradition that we were born with, breaking it slowly. It isn't something easy that can happen in one day.<br>**I: So if it can't happen in one day, since now we have had Corona for a while, from last year, so till today. Obviously you used to observe from then to now do you see any improvement or what?**<br>It is something difficult today I will follow, I remember seconds will pass I won't remember again, even you won't remember. That is why something that someone was born knowing it is difficult, to change. A year isn't long enough.
IDI 6 COMMUNITY MEMBER	I have 5 Children but the ones are just 3. Others are not here.

For peer review only

	By now no, I am a widow now. So I am just found with the babies.<br>**I: The baby, so which church do you pray from?**<br>R:<br>United Church of Zambia.<br>I am a business lady.<br>I would make even up to k6000 when I order items according to what I ordered when I sell them. It follow how they are, sometimes I used to order blankets from Joburg [/Johannesburg/] but it became difficult. Like starting from last, 2018, I didn't go to Joburg, this is when I have started with Fish.<br>So like at my place we follow, sanitizer we have, there is just somewhere we have put it, that liquid and the soap one, we have, so we use it.<br>Others what we are using we didn't buy, they distributed, they also gave us masks, like these ones, they said use them for one day don't use them continuous. They just gave us others that are mad of material and said you can use them for 3 days and the 3rd day you wash it if you have one and iron. Then you get another one.<br>**I: Uhmm Okay, those who taught you, they taught you about mask and sanitizing?**<br>Uhmmm other times washing, other times you just apply.<br>That is my child that is the only boy.<br>**I: The one who passed here?**<br>Leaving 2 girls plus me and this one [/Baby/].<br>**I: How many rooms does this house have?**<br>This house has 7<br>**I: 7 rooms? Okay. What about water to drink for cooking where do you fetch?**<br>Water where we draw from a house after this one, there is a tap.<br>**I: Behind there, okay, it is a tap or?**<br>It's a tap.

For peer review only

	**I: So, you told me that in the house there is hand sanitizer, everyone uses at home?**<br>Yes we have, I can even bring it for you to see.<br>Like the way I have started the fish I came with it yesterday, but long ago I used to bring goods, I used to board and go somewhere and (that's all) other will get from here, then that date when they say they will pay, they pay and give me the money.<br>Like no I know if will finish at home it is not a lot, it will finish just hear at home, if it was a lot would have carried it to there.<br>**I: So you receive different kinds of people in this home, when coming to buy fish.**<br>Uhm uhm, [/ No/] what I can say is it is not like they stay here at my home<br>We go by foot going to the market, unless we get something that is heavy that is when we book a taxi<br>Like? (Baby Crying) How can I give as an example? But many times I don't go, the girls are the ones who go buying relish stuff, uhmmm, they bring here at home. going I can go if I had arrived the day before yesterday today I would have gone for prayers. Wednesday but I have failed.<br>**I: So Wednesday you go for prayers?**<br>R:<br>Sunday then I go to church.<br>**I: What are the arrangements at church?**<br>R:<br>Due to issues of Covid we go in 3 services, following the number, only 50 enter with a mask, starts at 7hrs and 9:30 you come out, then when they come out, the next set of 50 enter and then after the last set enters. That is how we pray.<br>Even them go with masks to church at 17 hours and then come out at 18:30 just prayers.<br>**I: So, things that they follow are just masks.**<br>R:<br>Even sanitizers are there outside and in the church, it is finishing soon.<br>**I: Amen. What about weekends apart from church?**<br>R:

For peer review only

	We clean here and wash.<br>Kondwani (Baby Crying) even them they are strict with the mask and those testing you, will be found plenty in the road. They will test you, tell you wash hands and check your temperature. It is there eve there. In the road, till you get here to Chirundu.<br>I went alone, I didn't carry anyone, from home like let's go do business no I went alone (Baby Crying).<br>**I: So which measures did you follow, when travelling, how did you protect yourself?**<br>R:<br>Mask I have it right now the material, (Unclear Speech) I had said that since I arrived yesterday, I have not yet washed them, they are in my bag.<br>I don't want to lie, that is why we are given nicknames, that child I stay in the house, doesn't go anywhere. That baby doesn't come out of the house even when I went to the market remained in the house. I don't allow the mother, this is a baby it is not like your blood. Even me I can't get the baby and go to chat.<br>(Long Pause), mmm the way I am when people come, me I am a person I don't know how I am, even when someone knocks, to just say Ehhh. I peep on the window on the side to check whose there so that they can leave.<br>The reason I do that is because, that person where they are coming from I don't know, and other people will nick name you, if you tell them wash your hands. The person won't see it like I want me and them to be protected, they just going to see it like there is a way I have rated them. So I avoid, or sometimes people pass, rape tomato, and they don't have mask, when I go check I go look, I keep quiet and they will go.<br>But hearing I get to hear there is a funeral, but the same things that they restrict that don't go there to make a group.<br>No, nowhere, but I hear others leave this area, they say this one's mother passed through hear in the morning, at my place, they pass crossing this side. Other times, they will catch me coming from the toilet, ask them where are you going, to a funeral. Will even say they are boarding a truck, but me I always consider these movements that I am a single parent, my children<br>I can just say it is not like the way we lived long ago, like sitting anyhow, a group, too many as you want, and adhering to washing hands to protecting yourself, here and there. There is nothing we just used to ignore, but now you sit, sit, you remember where is

For peer review only

	that soap? I wash my hands, I wash, back then there wasn't it was only when you come out of the toilet, that is when you think of wash. But now no, you have to after sometime, you remember that where I have touched let me wash my hands, and other times we apply. It has become different from the old lifestyles.
IDI 7 COMMUNITY MEMBER	I am just a Pastors Wife and I am just a fullllll.<br>**I: Full Time Stay at home mum?**<br>R:<br>Anyway, I sell Salaula [/Second Hand Clothing/].<br>**I: Owooo.**<br>R:<br>But for now, I am just at home.<br>For now, we are 6.<br>**I: For now? Meaning?**<br>R:<br>Meaning maybe the other ones have grown they have left, but there are dependents whom I am keeping, I have 2 whom I am keeping then there are 2 of mine and then me and my husband. So for now we are six, because there are dependents but for now if it was me and my kids meaning we are supposed to be 4.<br>**I: Okay.**<br>R:<br>Yeah, because I am saying the other Children have grown and have left.<br>Yes, so we have 3 bedrooms, so these boys are 2 and the girls are also 2 then me am a married woman with my husband sharing same bed (short laugh). Sometimes same pillow.<br>Okay so for now, like me at my home, throughout I have water, because I sank a borehole, so throughout water I have but in the community, water is not available. Like here that is why we got forced because, here water is very difficult, too much so if you ask this one also, water is not available at their place. Its just gone, sometimes it comes back at night maybe 03hrs, but for people to

For peer review only

	manage to draw water its difficult. However, at my place throughout water I have because I sank a borehole, but in the entire compound, if you go to the taps you will find, there is no water.<br>**I: But they come to draw at the borehole?**<br>R:<br>Yes they come.<br>I don't know but at my home me I have, but I am sure in society not everyone has it. However at my home it's available because I am able to afford it, but I can't say everyone manages.<br>They don't have money, and this time there is a lot of challenges regards to money. People don't have money, business went down and people even if they work still more, they can manage for people to survive this life this time. It is very challenging this time where we have reached. That is why, I am saying I was selling but this time, I stopped why? Because the business has gone down, even if I order Salaula but when I opened the belle of clothing, there is nothing I will get out of it. Especially like business here in Chirundu sometimes we give on credit. When someone gets and you go try to get, they say they don't have, what are you going to do? You can't beat them, and for sure you can see that money isn't there and that is the biggest challenge that has come, people don't have money.<br>Nothing, that is what I am saying that they can't manage. No, no, they can't manage it's a big challenge actually, it's a big challenge.<br>**I: So you actually go to sell in town?**<br>R:<br>Nah ah, me I have a shop in the market.<br>Like me I have a car at home, so I go with the car, the big man, leaves me drops me off as he is going to his office.<br>**I: Where you work, do you work many of you in one shops or?**<br>R:<br>No, uhm uhm, me I have my own place, we build some shops, I can't say that we don't get too many because if so happens that the customers come. Even the familiar people, you just find that you are greeting them, you can't just let them go and in the market you

For peer review only

	maybe well known. Maybe the neighbor, has new ones so you go to the neighbor you see those things so that continuous meeting, I can't say we don't mingle with people. We mingle with people.<br>And especially this week, I have really been going to the market, this week I think starting from Sunday, Saturday I was at the wedding, there was a kitchen party wedding, they were children for the church. Sunday I didn't manage, I was feeling tired, Monday I was at Shoprite, Tuesday I was by Shoprite, Its just today when I have stayed home Wednesday. (Short Laugh).<br>Monday, Ahhhh, ah ah, there was just something I was doing, it was my husband's birthday, so I was buying, buying a few things.<br>**I: Then on weekends them.**<br>R:<br>Awe no I don't move, move, except this same Saturday when we had a wedding.<br>Yeah just the market if you want to buy something, you go and come back.<br>**I: You go there daily?**<br>R:<br>No, not daily.<br>**I: You don't know even like for 3 days like?**<br>R:<br>No, we are just for home full time, it's just hours just go buy something and return.<br>It depends just, maybe that day I go with the neighbor, another day, I am alone, another day I am with my husband. It just depends with the day and how it is moving.<br>**I: Okay.**<br>R:<br>Maybe there is something you go as a group, maybe, I go with the girls from church, you go maybe there is something you are buying, then you come back. But it is the market I am just going to the shop, I just go with my husband, he leaves me then he also goes to his office.

For peer review only

	That one is a big challenge. The way I am telling you that sometimes you can forget you laugh, you hit one another. Then you realize that, definitely you have bumped into one another, you chat, you meet familiar people you laugh. You can't really avoid a person, that thing is there for rubbing shoulders.<br>The neighbor it is this same one here, she has come from the door that straight door there. Oh if I leave my place I go to her place and if you are to find me, mainly I am there, but even the way we have sat. Isn't this one meter apart?<br>But moving if I am not at the market then it's just rare.<br>**I: So you meet daily?**<br>R:<br>This one its daily, if I don't go to her place, herself she will come here, you chat you chat.
IDI 8 MARKETEER	Shop Keeper.<br>**I: Then like in the business of the shop, in a month how much money do you make per month?**<br>R:<br>(Long Pause).<br>**I: When I speak of money I mean the money you as a person makes not the owner of the shop. The one that you use at home, how much income do you tend to have?**<br>R:<br>(Long Pause) k450.<br>So like me, I found a little difficulty, for the same mask, when I wear a mask I get a headache. (Long pause) so the way I have won this mask if I wear it too much I get a headache.<br>Me at my house there are only 3 people but with the landlord. So 4<br>**I: With the Landlord.**<br>R:<br>Ummm so we are 4.

For peer review only

	**I: So the landlord and you live in the same house?**<br>R:<br>Yes but the Landlord has their own house.<br>We are the 2 of, 3 of us.<br>**I: 3 of you.**<br>R:<br>Uhmmm.<br>**I: Then plus the room are 2?**<br>R:<br>They are 2 Ehee, [/Agreement].<br>**I: Water, where do you find water?**<br>R:<br>There are Taps where to draw water.<br>There is hand sanitizer, we wash our hands we put a bucket.<br>**I: A bucket and hand sanitizer, what else?**<br>R:<br>Masks are there and we sit, like (Unclear speech) since on Saturday that is when we sit together.<br>**I: Anything else that you can tell me, that you do to protect yourself from home?**<br>R:<br>There are visitors that come, we tell them to wear a mask and wash hands.<br>I don't greet in the hands<br>Ehhhh we greet each other from afar, and standing if someone comes to ask, here and there, we give social distance.<br>**I: Okay being as shop obvious you receive a lot of different people, how do you protect yourself and also help them?**<br>R:

For peer review only

We tell them to wash hands, to wear a mask and social distance.

I: Okay, then here what you have put like here at your workplace or in the market that this kind of thing has been put for protection that you see.
R:

What we put here, we put water outside, then hand sanitizer is there in the corner and the mask we tell them from the door to wear a mask.

Monday up to Friday, every morning I go to the bank.

I: What transport do you use when going to the bank?
R:

My foot it is near.

We observe social distance, we were mask and don't rub each other's hands when we get there.

The conditions, you find so many people, but we get it 5 (Unclear Speech) and you wait for another to enter.

I: So in the meantime as you are awaiting to get in, those people waiting where are they found?
R:

We sit outside.

I: You sit anyhow or there is a way that was put?
R:

You can sit anyhow.

I: What of?
R:

But when I get there I sit in my car (Unclear speech).

Because when you I wear this [/Mask/] it does not come out it is just circulating itself, not out, so it needs at least, to come out.

Me, being found home it is on Saturday morning up to 17:00 after I knock off from church and come here.

For peer review only

	Uhmm [/Yes/] I had stopped in between, because they had closed the churches, so when they opened we started wearing the masks and social distance, we observe.<br>**I: Do you attend functions such as weddings kitchen party, are there some you go for, In the past one month did you go?**<br>R:<br>No I don't go.<br>**I: Do you take alcohol, like you go out drinking?**<br>R:<br>No I don't drink alcohol.<br>Uhmm what we do when we have visitors, the masks and sitting in social distance.<br>**I: So if one comes and they don't have a mask, what do you do?**<br>R:<br>We buy.<br>**I: You buy for them?**<br>R:<br>Uhmmm, because it is my relative.<br>**I: Okay.**<br>R:<br>Like you have come you are my sister if you come home and you don't have a mask, (Unclear speech) I will buy that's all.<br>If doesn't have a mask?<br>**I: Well you find that you have friends and these are issues of the disease and the measures that are there how is it?**<br>R:<br>Uhmmmm there, (Laughs) we just stay that way.

For peer review only

	**I: Just like that?**<br>R:<br>The way we have sat just like this.<br>**I: Is there laughing, rubbing and touching hands?**<br>R:<br>Not holding each other's hands.
IDI 9 MARKETEER	SDA [/Seventh Day Adventist/].<br>**I: Then school where did you end from?**<br>R:<br>Me, uhmmm I am a tertiary University.<br>**I: (Laughs) Tertiary okay, what work do you do?**<br>R:<br>I have many jobs.<br>**I: Okay, you can tell us that one that sustains you.**<br>R:<br>They are a lot. Uhmmm they are so many that are sustaining me one, I work here, I do farming activities, then I do consultative services and so on and so on.<br>Serious? Maybe average uhmmm 6 pin [/K6000] and above.<br>Like ehhh washing hands I do it maybe every after 2hours or 3 hours because I am, for me I receive money, I receive money, from different source, I do not know where the hand is coming, whose hand it was in such. So I wash my hand maybe less than maybe 1 hours, 2 hours. And also uhmm I avoid touching my face, sensitive parts of my body. Such as negative parts like for example maybe I have a ka [/Small/] I try to squeeze it, I avoid that because I know to say my hands are not 100% safe, because I am touching a lot

For peer review only

of things. And the other thing is those measures that I have mentioned, like garlic, it is just that it is finished, I would have given you.

3 Bedrooms. 3 bedrooms house.

I: So you make sure that everyone in the house follows?

R:

Yes, whoever doesn't want to follow those rules, that is her own or his own, but myself and my daughter when I knock off before I touch her I wash my hands, I disinfect myself, then I wash her hands too, okay. Then we maintain a ka social distance.

Water we are using a borehole water, so it comes direct from the underground.

I: Owooo.

R:

It goes direct into the tank, uhm we wash our tank every two months inside, because that water that comes from the bore hole it comes with sand. You find that it is under at the bottom there, you wash it, you clean it inside. You put a bit of tu [/Little/] Chlorine ehh tablet chlorine the one we use, connected direct to the tank. Once water comes from the borehole it hits that first. That it when it goes into the tank.

Like I said here at the workplace, there is no social distance, people they are, not doing. So here mostly as you can see here, I am almost a distance ai?

I: Yes.

R:

I am almost a distance, so once I receive money from you, I give you the change whatever happens from then. After an hour I go wash my hands, on my side, but I do not know others. I always put on a face mask just that yesterday I lost my face mask when I went to someone's farm. I lost it, I don't know how I dropped it.

I walk by foot.

Uhmmm my movements are difficult, I go from home I sit, when I leave this places, I go in places, sometimes I go to the boarder, sometimes I go to the mall just like that.

For peer review only

	My movements are not very restricted.<br>Yes on a daily basis, everyday, everyday, except saturday, so everyday I start off coming here around 7 hours (Interruption) 7 hours to 19 hours.<br>**I: Okay.**<br>R:<br>Yah.<br>**I: You move alone? You come alone?**<br>R:<br>I move alone, sometimes when I find someone to escort me, we move the two of us. But mostly I move alone, sure.<br>Like I said earlier on, me I avoid hand shaking, maintain social distance, but it is not always that I should maintain social distance. And two like I explained earlier on when I got on a bus, I cannot avoid touching things. I will touch, I will have my support whenever the bus hits on a porthole, so after getting on the bus I will make sure that I will wash my hands. I also avoid touching sensitive parts such as my nose and the eyes.<br>No, on that one, all people that I have are my business friends, they come for business, they come here maybe for example he wants an advice. I advise that person do this and this and that, if it is physical inspections we will start off together lets go and see. If you want to marry you okay, I will advise you do this and this and that and that (unclear speech), such kind of stuff. If you come for consultation, okay, if you come for agriculture business we will move together and I will be visiting you inspecting you that is what I will do.<br>Where I have gone?<br>**I: Where you visit.**<br>R:<br>It depends, some they do take measures some they don't<br>Sometimes you visit people, mostly what I do I avoid touching things, like I explained earlier on I avoid touching things. I will go specific for that business, two I will avoid social, I will avoid meeting with different people, social distance also. Because I know

	Corona Virus it is really there and I have never felt how it feels, I have never gotten it, I do not want to have it because I know it will kill me okay. I have nowhere to go for me to be treated, so to avoid that better I maintain ka social distance. Avoid touching you if you are closer to me your hands will become my enemy.<br>Uhmmm no, with that one I have never done, from the time with the issues of corona I have never attended them or any other, wedding, wedding ceremony or funeral issues I have never attended.<br>Church I went once.<br>**I: From the time Corona started?**<br>R:<br>Yes, I went once and on that day, when I went to their church, people were not maintaining social distance and I was one of them. I did it I did what they do.<br>Serious, so to avoid that from that time I stopped going to church, because I knew that if I continue going to church if someone has it the church, I will get it and I will spread it also. So that in order to avoid, not to become, one of them who spreads Corona Virus then stay away just this period.
IDI 10 MARKETEER	High Cost.<br>**I: (Long Pause) (Paper Flipping), Then at home, how many of you live in there?**<br>We are 8.<br>**I: Then how old are you?**<br>I am 44.<br>**I: You look like a young girl. How many children do you have?**<br>4.<br>**I: Are you married?**<br>Yes.

For peer review only

	**I: Church, what church do you go to?**<br>Salvation.<br><br>**: Business woman?**<br>In a month, (Long Pause) I can say maybe 3 ping [/k3000/].<br><br>**I: Where you get water from?**<br>As in it is there or what?<br><br>**I: The water, that you use at home, where do you get it from?**<br>We get from the tap, just in the house it is there.<br><br>**I: So you have running water?**<br>Eeeaaaahhh. [/Yes/].<br><br>**I: What about the house you live in, how many rooms, does it have?**<br>It has (Long Pause), it has 3 bedrooms, together it is 6.<br>When the people come, we put a bucket outside, when they just arrive, even them they know, when they arrive, wash their hands and they are received.<br>They sit there, even you sit there, because there are many places to sit, if they are 2 of them one will sit here another, there even you sit like here, you chat they go, that's all.<br>(Chuckles), we are protecting ourselves in washing, just like that, in washing hands.<br><br>**I: Just washing, washing (Chuckles)?**<br>At home it is difficult, I can't lie, how can I say it (Laughs).<br><br>**I: Speak we hear.**<br>At home, just the same one for washing, social distance it, it's there just like that but, it's not often, like the way we have sat, just like this.<br>Coming with what?<br><br>**I: The transport that you use, to come to work.**<br>I come by foot [/walks/].

For peer review only

	Since when coming, I need to have applied sanitizer in my hands, in case I greet someone, so I just put in my hands, I am coming and it is in the handbag. So even that for greeting each other in the roads, it is over, it is now greeting each other like this or like that. You get here, maybe it is hello, hello, from afar.<br>Here, it is just putting a bucket outside and a soap, or maybe a Liquid Sanitizer, then the mask they are wearing.<br>**I: So now let me say, you come, since here you are selling clothes, you get to open the ballow, people are coming?**<br>R:<br>Coming.<br>**I: And when they become so many.**<br>R:<br>They are so many you can't even see them where they are entering from?<br>**I: Eh, what do you do?**<br>R:<br>Ah, on that part it is quite difficult, because here if I open ballow , where people are entering from, I won't see them, I will just notice them seated selecting. So protecting yourself truthfully, uhmm uhmm [/No/].<br>At home when you leave it is around 8, returning it is around 18. It is just at night.<br>We move by foot.<br>I was going, I was moving, because there are my friends selling in the market, you go to the market and chit chat you come back.<br>**I: How often do you often do you visit your friends?**<br>R:<br>These days, once they come just here, chat that's all, it isn't a lot of times.<br>Weekends Saturday maybe you come and sell, Sunday, morning you at church afternoon you home, Monday come here just like that.<br>We had all stopped church, this is when it just begun again, but still more there are laws for social distance just like that, they check, they come to check those from health.

For peer review only

	Social distance like we have sat here, now those with small church, but others do even 2 services, they get in and go out. Then the others also go in at 10:00 and come out.<br>Our church, our church is big, the one that we used to pray in is a small, but because of this same disease there is a church we are building and it hasn't been roofed, so that is where we went.<br>Handshakes people stopped.<br>**I: Or even those you know?**<br>R:<br>Sometimes you may forget, you like eh, eh but then immediately you realize that the hand is already there (Cross Talk).<br>**I: Oh even just the last one month, a wedding or a kitchen party.**<br>From the time it started I haven't.<br>**I: What about a funeral?**<br>To a funeral I went.<br>**I: How was it at the funeral?**
IDI 11 MARKETEER	It has 5 rooms. Serious, so to avoid that from that time I stopped going to church, because I knew that if I continue going to church if someone has it the church, I will get it and I will spread it also. So that in order to avoid, not to become, one of them who spreads Corona Virus then stay away just this period.<br>Each person has their own each person it is a bed sitter, each person their own, each person their own.<br>**I: So where you live in the house, you live with your kids?**<br>R:<br>No, I live alone

For peer review only

	But I am with my sister although even her she has her own room. But when its time to eat, we cook and eat together.<br><br>**I: Okay she is your sister?**<br>R:<br><br>Yes my elder sister.<br><br>**I: Okay, you are the 6 of you.**<br>R:<br><br>Alright she is also part of that number, the same one.<br><br>**I: How old are you? Years.**<br>I have 2 children.<br><br>**I: 2 alright, are you married, single, husband died, or separated?**<br>R:<br><br>Okay my husband died, I used to live in Livingstone, in 2004, so right now I live alone (customer interruption).<br><br>**I: So which church do you congregate?**<br>R:<br><br>SDA, Chirundu SDA.<br><br>Just this very job tailoring (Tailoring machine noise).<br><br>In a month I manage to make K2.500, if the business is okay.<br><br>So, most times, I prefer to be in a mask and one will come stand here and when you ask then, sit here, they don't want.<br><br>**I: The truck Driver.**<br>R:<br><br>Yes, so it is best to be wearing a mask all the time.<br><br>Eheee, yes again when you sit, and receive money from different people, a short time passes, you sanitize the hands. Just like that.<br><br>The rooms, okay the house is very big, each one has a door and other doors are outside.

For peer review only

	**I: So all the doors, all the rooms, have doors to go to the outside from?**<br>R:<br>No. Each of us use 1 door to get in, once you get inside there, there is a Corridor. There is a pass each person get into their room, each person their room.<br>**I: Alright so where do you get your water.**<br>R:<br>Water, is a problem, it is quite far where we have to fetch from. [Cross talk]<br>**I: It's a borehole and a tap?**<br>R:<br>It's a sometimes a borehole and sometimes a tap. so sometimes you find maybe, water went long ago, so we tend to draw from the borehole.<br>**I: its far? That is where you said it is far?**<br>R:<br>Yes, it is also a little quite further from home.<br>Yes we use buckets.<br>**I: Alright and when you draw?**<br>R:<br>Yeah so when we fetch just like that we store in the buckets and in the containers.<br>**I: So at home do you have hand sanitizing facilities, or even a bucket?**<br>R:<br>Well we don't have a bucket but sanitizer where when someone gets in water from the cup and washes their hands. With sanitizer.<br>**I: Okay, so how do you get to manage when there is no water, the whole day?**<br>R:<br>Anyway but mostly water is available for people to wash really when people arrive. We always make sure that water is available.

For peer review only

	In here I am alone, and the next shop is for my sister, but she works at the boarder so I just happen to sell for her. She works for Vet.<br><br>**I: Okay, So how do you come to work daily?**<br>R:<br><br>Huh?<br><br>**I: How do you come for work every day?**<br>R:<br><br>We just come by foot.<br><br>Mmmmmmm unless there is a problem, I don't usually move out.<br><br>**I: You don't move out of Chirundu?**<br>R:<br><br>Uhmmm.<br><br>**I: But whom do you go with?**<br>R:<br><br>Where I go? I go alone<br>Well I always make sure that I have sanitizer and a face mask, when I just get into the bus then I wear my mask.<br><br>**I: Okay so now I am going to ask you about, those that are important in your life. Maybe you have relative, neighbors, friends, how do you, How do you meet like how many times? Or daily, or relatives, how often do you meet them?**<br>R:<br><br>Uhmmmm, there are those like maybe they are just coming to visit, (Long pause), maybe in a week, they can come for visiting like 2 or 3 times. It sometimes happens like I am (unclear speech).<br><br>I am found I am here at work.<br><br>**I: Okay, so them they come at your place?**<br>R:<br><br>At home, but okay sometimes they come, even in the evening when we knock off. (Customer interruption)

For peer review only

	**I: So these who come are friends and relatives? They are not relatives?**<br>R:<br>No, they are not relatives, they are just friends.<br>**I: So these who come, they come every day?**<br>R:<br>No not every day.<br>Well they come, just like that, they visit and a short time they leave<br>Uhmmm Uhmm, Wedding and kitchen party No, maybe only at church.<br>**I: There was a meeting.**<br>R:<br>Just at church, we just went to go and pray.<br>**I: (long pause) just paying?**<br>R:<br>Yes just praying.<br>**I: So there, were they following the measures?**<br>R:<br>Yes.<br>**I: What were they doing (Tailoring machine noise).**<br>R:<br>When you go to church, you wash your hands, or use sanitizer, and when you get inside, each bench maybe only has 3 people. One at this far end the other in the middle and another on the other end. Then also wearing of the mask.
IDI 12 MARKETEER	**I: (Long Pause). In your house how many of you live there in total?**<br>13.

For peer review only

	**I: 13.**<br>Uhmmm.<br><br>**I: How old are you?**<br>29.<br><br>**I: 29, (Long Pause) how many children do you have?**<br>4.<br><br>**I: 4 Are you married? (Wind Blowing)**<br>Eaahee [/Yes/].<br><br>**I: What church do you go to?**<br>Apostles Church.<br><br>**I: What work do you do?**<br>Selling.<br><br>**I: So in this business you do, per month how much do you make?**<br>Per month, Profit?<br><br>**I: Eaahee [/Yes/].**<br>It goes even up to k500.<br><br>I wear masks on our mouth.<br><br>Yes, I have it, I move with it, because what happens is those from the council when they come, if they find us as we are in a group.<br><br>If you haven't won it for Corona, then it is a crime.<br><br>Like me on my own, hand sanitizer, I move with sanitizer in my hands, in my handbag, the thing for Corona [/Mask/], I move with it, but when I am alone, I take it off when I am with people, I wear it. Even the places we move about selling, I wear it, they don't allow moving without it. We helping each other from infecting, since when someone coughs, you can get that disease, so we protect ourselves, through the, the, mask. (Baby Crying), eaee [/Yes/] with hand sanitizer in the hands.<br><br>**I: Oh, Okay. So you can tell me your living at home, you said you live 13?**<br>R:

	Uhmm.<br>**I: So how do you live at home, the way you are 13, with the laws that are there?**<br>R:<br>How we live at home?<br>We get a bucket, like issues of the toilet we get the bucket, all children I told them whenever you come from the toilet you wash, because these children I cant manage their playing. When eating, wash your hands, (Baby Crying), don't go to peoples places, you will bring us the disease. Playing even just within here at home.<br>Yes, they are taps for the governments, they are written Kiyoski, so that's why.<br>Well it runs starting from 04 [/04:00/], when it is 7 hours the, again at 15 [/15:00/]<br>**I: That is when it comes?**<br>Yes.<br>**I: How many rooms does your house have?**<br>8<br>We come, arrive at the main market where we order from, when we get there, we wash our hands, that is when you begun to touch things to order.<br>**I: You move by foot?**<br>Eheaa [/Yes/].<br>Women are a group, they just rush, they just arrive, others don't even wash their hands, just orders, finishes and goes.<br>Many farmers come with mask.<br>**I: Them they protect themselves?**<br>Uhmmm, but us ordering, no it is a few.<br>We do, when you order things, you will sell, others that sleep over, what gets destroyed you go and throw, you order again, just like that. Just like that the little profit you find, you keep, the other buy food for the children.

For peer review only

	Yes every day, Saturday we don't sell, we stay home, we go to church. Then Sunday up to Friday, then Friday we knock off around 15 [/15:00/] or 16 [/16:00/] we go home, then again Sunday.<br>At church we contributed money, we bought buckets, sanitizer, we put, (Car Beeping), there are buckets for men and buckets for men. We put, the women wash when going to church, before the service, they wash hands. The service ends, you get the dirty water and throw it away and then, again on Saturday. Then for the mouth, also we wear [/Mask/].<br>**I: So when you knock off you greet each other or what?**<br>Yes.<br>**I: So you greet the same way with hands?**<br>Yes the same with hands.<br>**I: What about in church when you get in, how is it like? How do you sit?**<br>We sit 1 meter apart.<br>Ahhhhhh, they told us that we shouldn't reach 100 [/exceed 100/]. So there are two services others will go in the morning the others at 14 [/14:00/].<br>The mourning, we were there, but not so many, the health people [/Ministry of Health/] said people shouldn't be many, they need to reduce.<br>Even at the mortuary, only 6 women went there.<br>The ones that I am found with are the same ones, here, even in the field, we go like this we exchange.<br>**I: In the field?**<br>R:<br>Eaee [/Yes/].<br>**I: What do you go do in the field?**<br>R:<br>We go and sell.

For peer review only

	Me, like me where I go, I go in the offices, when someone calls, we carry and take [/deliver/]. They will select, tomorrow I go and collect the money.<br>In the office where we go, because there, the offices we to for are 4. So on those offices, they put a bucket and hand sanitizer, bucket sanitizer everyone. Then when you go there and you are many, they are going to get their masks and wear them on the mouth.<br>Like now, this time, things are hard, they are hard, back then, before Corona came, we were managing to move in the road freely, the council won't bother you and chase you. But now, should you try to assemble your goods it is a risk, the council will grab everything<br>Uhmmm [/Yes/] things are so that, like even selling, selling, the way we used to sell long ago, the business it not there, it is just for pushing, when you get a customer, when you don't have customers nicely, the business is difficult. Uhmmmm<br>Uhmmm when going to visit, I, when I am coming here in the market, I just pass through my mother. On Saturday if I don't go to church, I just sleep, that's all.<br>When I go to my mother we just live the same way that we have always lived.<br>No, nothing even at our place they usually go like, don't follow these things too much.<br>We just do it at church, but not that in this road, I don't see, this road? Nothing, even the way we sit when selling nothing. In churches because the health people [/Ministry of Health/], the tend to follow up a lot.
IDI 13 MARKETEER	**I: So at home, where you stay, how many of you stay there?**<br>4.<br>**I: You live the four of you?**<br>uhmmm.<br>**I: How old are you?**<br>Hmmm, I am 47.<br>**I: Yes, then how many children do you have?**<br>2.

For peer review only

I: Married?
Uhum.

I: Apostolic church of Zambia. (Long pause) then school you ended in which grade?
12.

I: You reached grade 12?
Uhumm.

I: Okay, Then occupation? What you do daily what you work as.
I sell just like this and also do tailoring.

I: then you like estimate like how much you get paid per month or how much you make per month? Or like from the lowest like you know in a month you can't go beyond this amount or below that amount.
R:

Hmmmmm I think the lowest in a month maybe its k150.

I: K150?
Yes that's the lowest in a month.

Water, we actually have.

I: Where is it?
There is it on the door.

I: You don't run out?
R:

It has 3 bedrooms.

I: 3 bedrooms and how do they sleep
R:

They sleep 1, 1.

I: Then at home where you draw water from, is water difficult or okay?
The water is available.

For peer review only

	**I: Alright so it is always available, it doesn't go?**<br>Well sometimes it goes, then sometimes it is there<br><br>**I: So when it goes how do you manage washing hands and the like from home?**<br>Just the way we made these buckets, because water, we get to know, that this time water will go, this time water will not be available so in the morning you just have to have drawn water, in the bucket. So you spare and know this is for washing hands, so even if someone comes they are going to know that this is for washing hands.<br><br>**I: When it goes, it goes from what time to what time?**<br>It is only 2 hours then it comes back.<br><br>When it finishes we have to refill it again.<br><br>I just come by foot.<br><br>**I: Then when coming, you come everyday?**<br>Everyday.<br><br>**I: Then also on the weekends?**<br>Hmmm except Sunday, Then Saturday Half day<br><br>I make sure, like the way they were teaching that Covid stays in the air for 30 minutes. So many times when I am leaving home I just have to wear a mask because I wont know which person had passed here, can find it has stayed in the air so many times I always have a mindset that I wear a mask. Because I don't know in that air maybe a person left the virus.<br><br>So every time, I leave home I just have to wear a mask, then when I reach there, I have to wash my hands.
IDI 14 MARKETEER	Okay the house we live in is big but each person has a room which they live. We are 5.<br><br>**I: So it has how many rooms?**<br>It has 5 rooms.<br><br>Each person has their own each person it is a bed sitter, each person their own, each person their own.

For peer review only

I: So where you live in the house, you live with your kids?
No, I live alone.

I: Okay.
But I am with my sister although even her she has her own room. But when its time to eat, we cook and eat together.

Just this very job tailoring (Tailoring machine noise).

I: Would you estimate how much money you can make, in a month, how much could it be just a rough estimation. Like it can go below this amount or above that amount.
In a month.

I: Yes.
In a month I manage to make K2.500, if the business is okay.

So, most times, I prefer to be in a mask and one will come stand here and when you ask then, sit here, they don't want.

So me wherever I go even if, someone comes, I always say people here is the bucket of water and soap wash your hands or even just sanitizing.

The rooms, okay the house is very big, each one has a door and other doors are outside.

I: So all the doors, all the rooms, have doors to go to the outside from?
No. Each of us use 1 door to get in, once you get inside there, there is a Corridor. There is a pass each person get into their room, each person their room.

I: Alright so where do you get your water.
Water, is a problem, it is quite far where we have to fetch from. [Cross talk]

I: It's a borehole and a tap?
It's a sometimes a borehole and sometimes a tap. so sometimes you find maybe, water went long ago, so we tend to draw from the borehole.

I: its far? That is where you said it is far?
Yes, it is also a little quite further from home.

I: Okay so you go there with buckets.

For peer review only

	Yes we use buckets.<br>Yeah so when we fetch just like that we store in the buckets and in the containers.<br>**I: So at home do you have hand sanitizing facilities, or even a bucket?**<br>Well we don't have a bucket but sanitizer where when someone gets in water from the cup and washes their hands. With sanitizer.<br>Anyway but mostly water is available for people to wash really when people arrive. We always make sure that water is available.<br>Well I always make sure that I have sanitizer and a face mask, when I just get into the bus then I wear my mask.<br>Uhmmmm, there are those like maybe they are just coming to visit, (Long pause), maybe in a week, they can come for visiting like 2 or 3 times. It sometimes happens like I am (unclear speech).<br>**I: You are found what?**<br>R:<br>I am found I am here at work.<br>**I: Okay, so them they come at your place?**<br>R:<br>At home, but okay sometimes they come, even in the evening when we knock off. (Customer interruption)<br>**I: So these who come are friends and relatives? They are not relatives?**<br>R:<br>No, they are not relatives, they are just friends.<br>**I: So these who come, they come every day?**<br>R:<br>No not every day.<br>**I: For example in the past one month, how many times did they come?**<br>R:<br>In the past one month? Mmmmm maybe I can just say in a week 2 or 3 times.

For peer review only

	**I: Per week?**<br>R:<br>Eheeaaa.<br>**I: So each week they used to come in each week 3 times?**<br>R:<br>Like those that live in Chirundu?<br>Well they come, just like that, they visit and a short time they leave.<br>**I: Did you go like to a wedding in the past one month, anywhere where people gather a lot? Whether it is a wedding, kitchen party or church meeting?**<br>R:<br>Uhmmm Uhmm, Wedding and kitchen party No, maybe only at church.<br>**I: There was a meeting.**<br>R:<br>Just at church, we just went to go and pray.<br>**I: (long pause) just paying?**<br>R:<br>Yes just praying.<br>When you go to church, you wash your hands, or use sanitizer, and when you get inside, each bench maybe only has 3 people. One at this far end the other in the middle and another on the other end. Then also wearing of the mask.
IDI 15 MARKETEER	**I: Then where you stay, how many are you?**<br>We are 3.<br>**I: Then how old are you?**<br>I am 25 years.<br>**I: Owooo.**<br>(Short laugh) I look too old or too young.

	**I: No, it's just that is my age too.**<br>(Sharp laugh).<br><br>**I: Then you have how many children?**<br>I have one.<br><br>**I: Grade 7, okay, then what is your occupation?**<br>Business.<br><br>**I: Business. Then could you estimate like per month you make this amount or that amount. Every month how much do you think you make.**<br>**Maybe k3000.**<br><br>**I: Then how big is the house and how many rooms?**<br>R:<br><br>Uhmmmm ehh 5 rooms. 5 rooms.<br><br>**I: 5 rooms with how many bedrooms.**<br>R:<br><br>Uhmmmmm It has 3 bedrooms.<br><br>**I: You are doubting?**<br>R:<br><br>It has 3 bedrooms. I am not doubting.<br><br>**I: So how do you sleep?**<br>R:<br><br>Uhmmm (Laughs)<br><br>**I: Everyone has a room.**<br>R:<br><br>Yes everyone has a room.<br><br>**I: Then at home, what is there for you to use to follow the guidelines by the government.**

For peer review only

At home the masks are there, sanitizer is there, water you will find it at the corner of the house. Everything is there, so we follow.

I: So you don't need any efforts from Ministry of health since the community manages everything. Water, sanitizer they manage to buy.
Ah, now those things is there even so much money, but anyway others don't manage but on my part there is nothing I can't afford not to buy.

I: Then at work, do you share, this office? Your shop?
Here no, when I leave and go for orders, I leave it for my wife.

I: So there is no time that you share it and find yourselves the two of you?
No, I am just alone.

I: Okay, how do you come for work daily?
Daily I come here at 07:30.

I: What transport do you come with?
My home is near, so I walk here.

I: You own a car?
Just Cars.

I: Taxis and Buses?
Yes even buses as well?

I: Then whom do you usually travel with?
There, sometimes when I am going for orders I go, every week on Monday, I go to Lusaka, you come back as long as you have a mask. Maybe when I am alone like this, I don't wear a mask but when I am in the bus I wear the mask, you go there and return without taking it off.

I: So you go to Lusaka every week? (Music playing)
I go every week.

Ahhh no we just talk on the phone, even maybe when they come here, we just greet each other. He's there, I am also here, we greet each other.

For peer review only

	**I: What about at your home, your neighbor or relatives.**<br>Coming at home they come, but they need that once they arrive, they will need to wash their hands and not sit too near one another.<br>So we tend to follow those.<br>Ahhh no we just talk on the phone, even maybe when they come here, we just greet each other. He's there, I am also here, we greet each other.<br>**I: What about at your home, your neighbor or relatives.**<br>R:<br>Coming at home they come, but they need that once they arrive, they will need to wash their hands and not sit too near one another.<br>So we tend to follow those.
**summary**	Peoples' livelihoods have not changed much because of COVID-19.Those who are public transporters have not stopped traveling, including those who are involved in business. Most homes attempted to implement preventive measures like hand washing although social distancing is still a challenge in homes. Availability of water in most homes was a challenge as most of them do not have access to personal water facilities. Water supply was not adequate in most homes. Attending to social responsibilities like church, funerals and visiting family and friends continued to a a large extent

**3. SOCIAL NETWORKS**		
**Resource**		
IDI1 IMMIGRATION OFFICER	At the village, we had a funeral.	
IDI2 TRUCK DRIVER	Home, home myself I don't go anywhere, I am always away at home, so that is the time for me to be at home. I am always away, like 1 month I am away from home, when I reach home, I have to be at home. So that I get familiar of my children and whatever is happening at my home, even beer, like I go buy my beer I drink at home.<br>That's what I do, I don't go to drink beer like to public places, I drink from home, I am an old man.<br>Home, if my weekend find me home I am drinking beer with my wife.<br>It won't be a party if we invite other people, so it's only for my family, so that they know that daddy is around, I buy them meats, we braai and even beer we are chatting there, that's all I spend 2 days and off I go.	

For peer review only

	The people I talk to social media?<br>**I: No, relate, the people that you relate with, chat with.**<br>R Uhmm most of the time I chat with my friends, the drivers, and ehh uhmm, most of the time, (Unclear Speech) we will be together with my friend, because those are the ones that we chat with. The drivers mostly, and query agents that's all<br>I go to my truck, but people, but people sometimes I talk to my fellow drivers, those are the ones that I (Unclear speech) don't do that.<br>I am always in the truck the only funeral that happened, I assist with money since I wont be there, or maybe, my wife sometimes also goes there, that's all. Me I won't be there because sometimes a funeral happens maybe when I am in Congo, for me to go fast to attend that funeral (Unclear Speech) I will find that person buried. Then I will only maybe go to say my condolences, what what, you call it, that's all.<br>Ahhhmost time, my truck is my church I worship, lord help me and he is always with me
IDI 3 TRUCK DRIVER	The church is closed, churches are closed, they don't allow, gathering at church.<br>**I: what if it so happens that you visit your relatives?**<br>We visit but not so often.<br><br>We visit but not so often.
IDI 4 TRUCK DRIVER	The only friends I have are these same workmates, the truck drivers am in contact with. Then sometimes I can go with them but maybe their papers are not ready so I leave them and proceed. Even them if my papers are not ready they will leave me behind just like that life goes on. When I am in Zambia on Sunday, I go to church but honestly I haven't been to church in 3 months so far.
IDI5 TRUCK DRIVER	Alcohol I drink a lot if you buy for me, you will see how I will treat it.<br>**I: So whom do you drink with?**<br>At home, from a long time ago if I feel like drinking, its at home.

IDI 6 COMMUNITY MEMBER	Uhmmmm, there are those like maybe they are just coming to visit, (Long pause), maybe in a week, they can come for visiting like 2 or 3 times. It sometimes happens like I am (unclear speech).<br>**I: You are found what?**<br>I am found I am here at work.<br>**I: Okay, so them they come at your place?**<br>At home, but okay sometimes they come, even in the evening when we knock off. (Customer interruption)<br>No not every day.<br>**I: For example in the past one month, how many times did they come?**<br>In the past one month? Mmmmm maybe I can just say in a week 2 or 3 times<br>Uhmmm Uhmm, Wedding and kitchen party No, maybe only at church.<br>**I: There was a meeting.**<br>Just at church, we just went to go and pray.
IDI 7 COMMUNITY MEMBER	Due to issues of Covid we go in 3 services, following the number, only 50 enter with a mask, starts at 7hrs and 9:30 you come out, then when they come out, the next set of 50 enter and then after the last set enters. That is how we pray.<br>There is nowhere I have gone, where I have been invited to a wedding kitchen party.<br>**I: What about a funeral?**<br>Ahhh, isn't it the same place where they say if you hear about the funeral don't go they don't want number [/Crowds/].
IDI 8 MARKETEER	The neighbor it is this same one here, she has come from the door that straight door there. Oh if I leave my place I go to her place and if you are to find me, mainly I am there, but even the way we have sat. Isn't this one meter apart?<br>**I: Yes (short laugh).**<br>But moving if I am not at the market then it's just rare.<br>**I: So you meet daily?**<br>This one its daily, if I don't go to her place, herself she will come here, you chat you chat.

For peer review only

	**I: So at the wedding, where you said you went, did you only go to the wedding or maybe there was a funeral like that also. Anywhere where groups are found, you said that you went to a Wedding and Kitchen Parties?**<br>Yes they were the same day for the same person.<br>**I: Oh it was the same day?**<br>Yes it was the same day.<br>At church there was a church service in the morning, then in the afternoon [Cross Talk] there was a kitchen party then at night, there was a reception.<br>No, they were different here and there, like the kitchen party they were different because a kitchen party there are no men there. (Baby Crying).
IDI 9 MARKETEER	Uhmm [/Yes/] I had stopped in between, because they had closed the churches, so when they opened we started wearing the masks and social distance, we observe.
IDI 10 MARKETEER	**I: How often do you often do you visit your friends?**<br>R:<br>These days, once they come just here, chat that's all, it isn't a lot of times.<br>**I: Okay, So even moving you don't move about with your friend?**<br>No they just usually come to the shop.<br>**I: Okay. Then on the weekends where do you spend your time? Since during the week.**<br>Weekends Saturday maybe you come and sell, Sunday, morning you at church afternoon you home, Monday come here just like that.<br>**I: Then at church from the time Corona begun till today, have you been going?**<br>Uhm uhm [/No/] because those days the government stopped us from going to church isn't it?<br>**I: Uhmmm.**<br>We had all stopped church, this is when it just begun again, but still more there are laws for social distance just like that, they check, they come to check those from health.

	To a funeral I went.
IDI 11 MARKETEER	Sometimes you visit people, mostly what I do I avoid touching things, like I explained earlier on I avoid touching things. I will go specific for that business, two I will avoid social, I will avoid meeting with different people, social distance also. Because I know Corona Virus it is really there and I have never felt how it feels, I have never gotten it, I do not want to have it because I know it will kill me okay. I have nowhere to go for me to be treated, so to avoid that better I maintain ka social distance. Avoid touching you if you are closer to me your hands will become my enemy.<br>**I: Okay.**<br>Uhmmm no, with that one I have never done, from the time with the issues of corona I have never attended them or any other, wedding, wedding ceremony or funeral issues I have never attended.<br>Serious, so to avoid that from that time I stopped going to church, because I knew that if I continue going to church if someone has it the church, I will get it and I will spread it also. So that in order to avoid, not to become, one of them who spreads Corona Virus then stay away just this period.
IDI 12 MARKETEER	Yes every day, Saturday we don't sell, we stay home, we go to church. Then Sunday up to Friday, then Friday we knock off around 15 [/15:00/] or 16 [/16:00/] we go home, then again Sunday.<br>**I: You pray on Saturday?**<br>When I went to visit, I didn't go visiting I had gone to a funeral.<br>Uhmmm when going to visit, I, when I am coming here in the market, I just pass through my mother.
IDI 13 MARKETEER	Many times when I am in my yard, then they are in their yard and we are talking. So most times social distance is observed.<br>Just like I told you in the beginning that at church there is no problem, when it comes to following those rules. Each time when going to church, each person tends to adhere to those rules.<br>Even last Sunday we had one.<br>**I: Was it a wedding?**<br>R:<br>Just a meeting.

	**I: Yes, oh within Chirundu maybe you had gone at the neighbors.**<br>Ohooo, yes I did go.
IDI 14	When you go to church, you wash your hands, or use sanitizer, and when you get inside, each bench maybe only has 3 people.<br>Uhmmm Uhmm, Wedding and kitchen party No, maybe only at church.<br>**I: There was a meeting.**<br>Just at church, we just went to go and pray.<br>**I: (long pause) just paying?**<br>Yes just praying.<br>**I: Or even outside Chirundu, everyone really, even if it is just the neighbor.**<br>Well they come, just like that, they visit and a short time they leave.<br>No, they are not relatives, they are just friends.<br>Uhmmmm, there are those like maybe they are just coming to visit, (Long pause), maybe in a week, they can come for visiting like 2 or 3 times.<br>**I: So these who come, they come every day?**<br>No not every day.<br>**I: For example in the past one month, how many times did they come?**<br>In the past one month? Mmmmm maybe I can just say in a week 2 or 3 times.<br>Mmmmmmm unless there is a problem, I don't usually move out.
IDI 15 MARKETEER	**I: So I will proceed to ask you about a group probably a wedding, a place with a group of people, or a kitchen party or meeting in the market that you attended.**<br>R:<br>No, I didn't attend on any, I didn't attend anything.<br>**I: What about your friends, the ones that you interact with, your relatives, your neighbors, how do you maintain interaction? Do you just talk on the phone or they come home to visit here and there.**<br>R:

For peer review only

	Ahhh no we just talk on the phone, even maybe when they come here, we just greet each other. He's there, I am also here, we greet each other.<br><br>**I: What about at your home, your neighbor or relatives.**<br>R:<br>Coming at home they come, but they need that once they arrive, they will need to wash their hands and not sit too near one another. So we tend to follow those.<br><br>**I: Then what about this month can you estimate how many came at home?**<br>R:<br>Hmmmmmm ehhh, I don't think they are a lot, maybe just 5 of them.<br>Sometimes they can just come and greet, and go, just come and go after greeting.
**summary**	Most participants indicated that they have maintained their social networks, though visitations have reduced. Most stopped attending church meetings but still attended to social responsibilities like attending funerals and weddings once invited. Visitations by friends and families continued to a large extent.

**4. TRAVEL PATTERNS**		
**RESOURCE**		
IDI 1 IMMIGRATION OFFICER	**I: So it is above (Long pause) have you ever travelled of the town this month?**<br>This month no but July yes.<br><br>**I: Okay, in July where did you go?**<br>At the village, we had a funeral.<br><br>**I: Where?**<br>Pemba.<br><br>**I: What mood of transport do you use to come everyday?**<br>At times I walk, at times I drive	

For peer review only

	**I: What mood of transport do you use to come everyday?**<br>At times I walk, at times I drive<br>Hmmm in Pemba I was there for 3 days.<br>**I: Okay.**<br>Then Kariba, Just one day. Lusaka, I can just say Kariba Few hours and Lusaka few hours then I came back.<br>**I: Okay, what transport did you use?**<br>Hmmm Personal.<br>Uhmmm when going to the village, I used public transport. But when going to Kariba, we were two of us in the vehicle.
IDI2 TRUCK DRIVER	I stay in Harare, Zimbabwe.<br>Yes I have gone to South Africa.<br>Yes, South Africa, since I am a traveler I am always moving.<br>I repeat Zambia Congo, South Africa, South Africa just like that.<br>July I was in Congo and then I (Unclear Speech).<br>**I: Connected to South Africa.**<br>R:<br>Connected to South Africa that same month, then I came<br>I went uhmm I spent somewhere like 2 weeks in South Africa, I think around 18 there. Yah, around 18 there, I went out, (Unclear speech) time I was coming.<br>There is nothing we can do because, found me working already as a traveler as a truck driver (Unclear Speech) so there is no way I can't, because Covid came, I can't quit my job, how am I going to feed my family?
IDI 3 TRUCK DRIVER	We left, started off going to South Africa, I passed through Zimbabwe.<br>That company is in Kabwe then me I stay in Ndola, when I leave the vehicle there, I get to board a bus taking me to Ndola then following day again, or that other day, that is when I start off back to come to Kabwe.

For peer review only

	**I: Hmmm. You get on the public bus?**<br>R:<br>The same big public buses the big ones.<br>**I: So in the bus, how is it?**<br>R:<br>In the bus they don't allow the normal number that it was long ago, the number gets less, so that there should be spaces not sitting near one another.
IDI4 TRUCK DRIVER	I have been to Congo, South Africa, Zambia.<br>I can't remember Departure but I was there for 2 weeks, actually this is me arriving back from South Africa I still need to get to Lusaka and proceed to Congo.<br>Every month I travel out I take good there mainly like I said Congo Zambia, South Africa.<br>I am working for 2 weeks am out I just go sleep maybe for one night and then proceed.
IDI 5 TRUCK DRIVER	No me at home I just sleep, 2 nights.<br>**I: Owooo.**<br>**I: Okay leaving town within Zambia where did you go?**<br>South Africa.<br>This one that finished Congo, Zimbabwe, South Africa.<br>To South Africa, Passing through Zimbabwe.<br>**I: How long did you stay in South Africa?**<br>R:<br>South Africa the days can be up to 10.
IDI 6 COMMUNITY MEMBER	Recently I can say, I arrived yesterday from Mongu, past 21<br>I just stayed there only 2 weeks.<br>**: Uhmmm (Baby Crying) so when going to order what transport do you use?**<br>R:

For peer review only

	The big buses<br>R: We go by foot going to the market, unless we get something that is heavy that is when we book a taxi.
IDI 7 COMMUNITY MEMBER	Like me I have a car at home, so I go with the car, the big man, leaves me drops me off as he is going to his office.<br>And especially this week, I have really been going to the market, this week I think starting from Sunday, Saturday I was at the wedding, there was a kitchen party wedding, they were children for the church. Sunday I didn't manage, I was feeling tired, Monday I was at Shoprite, Tuesday I was by Shoprite, Its just today when I have stayed home Wednesday. (Short Laugh).<br>Awe no I don't move, move, except this same Saturday when we had a wedding<br>Yeah just the market if you want to buy something, you go and come back.
IDI 8 MARKETEER	**I: Where did you go?**<br>Lusaka.<br>**I: What about at home where you are, when you leave home coming to work what transport do you use?**<br>There is no transport that I use, it is near.<br>**I: Even by foot it is transport.**<br>Even by feet it is transport (Laughs).<br>**I: It is also transport, so you come by foot.**<br>Yes I come by foot.<br>Monday up to Friday, every morning I go to the bank.<br>**I: What transport do you use when going to the bank?**<br>My foot it is near.
IDI 9 MARKETER	**I: Where did you go?**<br>I went to Lusaka, Chisamba.<br>Uhmmm my movements are difficult, I go from home I sit, when I leave this places, I go in places, sometimes I go to the boarder, sometimes I go to the mall just like that.

For peer review only

	**I: Owooo.**<br>My movements are not very restricted.
IDI 10 MARKETEER	(Laughs) I had gone to Livingstone.<br><br>**I: The transport that you use, to come to work.**<br>I come by foot [/walks/].<br><br>**I: So that one for going to Livingstone, what transport did you go with?**<br>Here I got a small bus, I got to 10 park, got on a big one.
IDI 11 MARKETEER	**I: Where did you go?**<br>Lusaka, the day before yesterday.<br><br>**I: How do you come for work every day?**<br>We just come by foot.<br><br>**I: So you never get to use a Taxi? Sometimes.**<br>Well sometimes, like today I came with the taxi because the machine was at home.
IDI 12 MARKETEER	**I: In the last month, this month that has just past?**<br>Yes I did.<br><br>**I: Where did you go?**<br>Lusaka.<br><br>When I went to visit, I didn't go visiting I had gone to a funeral.<br><br>**I: When did you go?**<br>Last month, I am 3 weeks old here.<br><br>**I: One month has not yet met.**<br>No.<br><br>**I: Where was the funeral?**<br>Kabanana.

For peer review only

	**I: Kabanana just here in Chirundu?**<br>No Kabanana in Lusaka.<br>**I: So when you went, you boarded a bus or what?**<br>**I: The IC bus?**<br>R:<br>Yes from the Station.<br>When we were leaving the conductor said, anyone who doesn't have a mask, shouldn't get into the bus. When we got in the bus we wore cloths for Corona [/Mask/] until we reached Lusaka. At the funeral, it finished and we all had those cloth, the health people came to check if people have clothes for Corona. It was found that we all have, anyone who didn't have was chased, even at the mortuary, when we went, anyone without the cloth for Corona was being chased.<br>**I: How did you sit, in the bus?**<br>In the bus we had sat the same way, we sit all the other days.<br>**I: Owooo, nothing changed?**<br>Eaee [/Yes/], four, four.
IDI 13 MARKETEER	**I: Did you leave the town of Chirundu this past month?**<br>Hmmm Last month?<br>**I: Yes.**<br>No Hmmm Hmmm.<br>No, last month no.<br>I just come by foot.<br>**I: Then when coming, you come everyday?**<br>Everyday.<br>**I: Then also on the weekends?**<br>Hmmm except Sunday, Then Saturday Half day

For peer review only

IDI 14 MARKETEER	**I: Where did you go?**<br>Lusaka, the day before yesterday.<br><br>**I: How do you come for work every day?**<br><br>We just come by foot.<br><br>Mmmmmmm unless there is a problem, I don't usually move out.<br><br>Where I go? I go alone.<br><br>**I: Wherever you go? You don't go with sister, where you go?**<br>No she remains.<br><br>**I: Uhmmmm, so in your movements what do you do to protect yourself from Corona? (Tailoring machine noise)**<br>Well I always make sure that I have sanitizer and a face mask, when I just get into the bus then I wear my mask.
IDI 15 MARKETEER	**I: Then in the month of July did you leave Chirundu town?**<br>No at all.<br><br>**I: You didn't leave Chirundu?**<br>No.<br><br>**I: What about the country? Did you leave the country?**<br>No I didn't.<br><br>**I: Okay. (Small Laugh) You didn't go to Zimbabwe?**<br>They are difficult in Zimbabwe we don't go at all.<br><br>**I: Okay, they are difficult?**<br>They are difficult.<br><br>**I: Okay, how do you come for work daily?**<br>Daily I come here at 07:30.

For peer review only

	**I: What transport do you come with?**<br>My home is near, so I walk here.<br>**I: Every day you come by foot? Or even the weekends.**<br>Ahhhh, its not far from here, so I walk.<br>Owoooo, As in the vehicle I use when I travelling. I use a car sometimes. (Music playing).<br>**I: You own a car?**<br>Just Cars.<br>**I: Taxis and Buses?**<br>Yes even buses as well?<br>There, sometimes when I am going for orders I go, every week on Monday, I go to Lusaka, you come back as long as you have a mask. Maybe when I am alone like this, I don't wear a mask but when I am in the bus I wear the mask, you go there and return without taking it off.<br>**I: So you go to Lusaka every week? (Music playing)**<br>I go every week.<br>Transport it is these same ones for the buses.<br>**I: Whom do you move with?**<br>I move alone.
**summary**	Travel patterns remained the same .Most participants still travel for various reasons, to attend to family matters, for business and work. Most used public transport where social distance was difficult to maintain.

For peer review only

Standards for Reporting Qualitative Research (SRQR)

Topic	Item
**Title and abstract**	
Title	✓
Abstract	✓
**Introduction**	
Problem formulation	✓
Purpose or research question	✓
**Methods**	
Qualitative approach and research paradigm	✓
Researcher characteristics and reflexivity	✓
Context	✓
Sampling strategy	✓
Ethical issues pertaining to human subjects	✓
Data collection methods	✓
Data collection instruments and technologies	✓
Units of study	✓
Data processing	✓
Data analysis	✓
Techniques for enhancing trustworthiness	✓
**Results/findings**	
Synthesis and interpretation	✓
Links to empirical data	✓
**Discussion**	
Integration with prior work, implications, transferability and contributions to the field	✓
Limitations	✓
**Other**	
Conclusion	✓
Author contribution	✓
Conflict of interest	✓
Funding	✓

For peer review only

BMJ Open

Perspectives on Factors Influencing Transmission of Coronavirus disease 19 (COVID-19) in Zambia: A qualitative study of Health Workers and Community Members

Journal:	*BMJ Open*
Manuscript ID	bmjopen-2021-057589.R2
Article Type:	Original research
Date Submitted by the Author:	07-Mar-2022
Complete List of Authors:	Sialubanje, Cephas; Levy Mwanawasa Medical University, School of Public Health<br>Sitali, Doreen; University of Zambia, School of Public Health<br>Mukumbuta, Nawa; Levy Mwanawasa Medical University, School of Public Health<br>Liyali, Libonda; Eden umiversity, School of medicine<br>Sumbwa, Phyllis; University of Zambia<br>Kamboyi, Harvey; Eden University, Disease Control and Prevention<br>Ng'andu, Mary; Levy Mwanawasa Medical University, Health Sciences<br>Goma, Fastone; Eden university, School of Medicine
**Primary Subject Heading**:	Infectious diseases
Secondary Subject Heading:	Public health
Keywords:	COVID-19, INFECTIOUS DISEASES, Public health < INFECTIOUS DISEASES, QUALITATIVE RESEARCH

SCHOLARONE™
Manuscripts

I, the Submitting Author has the right to grant and does grant on behalf of all authors of the Work (as defined in the below author licence), an exclusive licence and/or a non-exclusive licence for contributions from authors who are: i) UK Crown employees; ii) where BMJ has agreed a CC-BY licence shall apply, and/or iii) in accordance with the terms applicable for US Federal Government officers or employees acting as part of their official duties; on a worldwide, perpetual, irrevocable, royalty-free basis to BMJ Publishing Group Ltd ("BMJ") its licensees and where the relevant Journal is co-owned by BMJ to the co-owners of the Journal, to publish the Work in this journal and any other BMJ products and to exploit all rights, as set out in our licence.

The Submitting Author accepts and understands that any supply made under these terms is made by BMJ to the Submitting Author unless you are acting as an employee on behalf of your employer or a postgraduate student of an affiliated institution which is paying any applicable article publishing charge ("APC") for Open Access articles. Where the Submitting Author wishes to make the Work available on an Open Access basis (and intends to pay the relevant APC), the terms of reuse of such Open Access shall be governed by a Creative Commons licence – details of these licences and which Creative Commons licence will apply to this Work are set out in our licence referred to above.

Other than as permitted in any relevant BMJ Author's Self Archiving Policies, I confirm this Work has not been accepted for publication elsewhere, is not being considered for publication elsewhere and does not duplicate material already published. I confirm all authors consent to publication of this Work and authorise the granting of this licence.

For peer review only

Perspectives on Factors Influencing Transmission of Coronavirus disease 19 (COVID-19) in Zambia: A qualitative study of Health Workers and Community Members

Cephas Sialubanje[1*], Doreen C Sitali[2], Nawa Mukumbuta[3], Libonda Liyali[4],Phyllis Ing'utu Sumbwa[5], Harvey Kakoma Kamboyi[4], Mary Ngandu[1], Fastone M. Goma[4]

[1]Department of Disease Prevention, Levy Mwanawasa Medical University, School of Public Health, P.O. Box 33991, Lusaka, Zambia

[2]Department of Health Promotion, University of Zambia, School of Public Health, P.O. Box 32379, Lusaka, Zambia

[3]Department of Biostatistics and Epidemiology. School of Public Health and Environmental Studies. Levy Mwanawasa Medical University. P. O. Box 33991, Lusaka. Zambia 10101.

[4]Department of Disease Control and Prevention, School of Medicine and Health Sciences, Eden University, P.O. Box 37727, Lusaka, Zambia

[5]Department of Disease Control and Prevention, School of Education, University of Zambia, P.O. Box 32379, Lusaka, Zambia

*Corresponding Author: Cephas Sialubanje; csialubanje@yahoo.com

Mobile: +260 977 441 273

For peer review only

Abstract

Objective: To explore the health professionals' and community members' perspectives on the factors influencing transmission of the novel coronavirus disease 2019 (COVID-19) in Zambia.

Design: An exploratory qualitative study using in-depth interviews as data collection technique.

Setting: Four primary health care facilities and local communities of Lusaka city and Chirundu international border town under Lusaka province, Zambia.

Participants: Purposive sampling of sixty study participants comprising health professionals (n=15) and community members (n=45). Health staff were health inspectors and surveillance officers. Community members included public market traders, civic and religious leaders, immigration officers, bus and international truck drivers.

Results: Both health professionals and community members were aware of the COVID-19 pandemic, the preventive and control measures. Nevertheless, stark differences were observed on the two groups' perspectives on COVID-19 and the factors influencing its transmission. Most health staff expressed high personal risk and susceptibility to the disease and a positive attitude towards the prevention and control measures. Conversely, myths and misconceptions influenced most community members' perspectives on the disease and their attitude towards the COVID-19 guidelines. Participants were unanimous on the low levels of adherence to the COVID-19 preventive and control measures in the community. Reasons for non-adherence included limited information on COVID-19, negative attitude towards COVID-19 guidelines, social movement and travel patterns, networks and interactions, living and work conditions, water and sanitation facilities, and observation of behaviours of important role models such as politicians and other community leaders. These factors were perceived to increase the risk of COVID-19 transmission.

Conclusion: These findings highlight important factors influencing transmission of COVID-19 in Zambia. Future interventions should focus on providing information to mitigate myths and misconceptions, increasing people's risk perception to the disease, and improving attitude towards the prevention and control interventions and mitigating structural and socioeconomic barriers.

Word count: 294

Key words: COVID-19, transmission, factors, qualitative research, Zambia

For peer review only

Strengths and Limitations of the study

- The qualitative study design allowed for in-depth exploration and analysis of the subject under investigation
- Our purposive sampling of study sample comprising health workers and community members from both urban and rural sites, with varied socioeconomic backgrounds and experiences with regard to COVID-19 allowed for triangulation and increased the validity of the study
- Selection of research assistants experienced in qualitative methods, training them in theoretical and practical aspects of our study as well as supervision of data collection process by an experienced research team minimised bias and assured internal validity of the study
- Conducting the study during the first wave of the COVID-19, when the disease was still new and the number of cases and deaths was still low in the country may have limited would have introduced bias into the study as the community members' perspectives may have changed over the three successive waves of COVID-19 experienced in the country

For peer review only

For peer review only - http://bmjopen.bmj.com/site/about/guidelines.xhtml

Background

Severe Acute Respiratory Syndrome Coronavirus 2 (SARS-CoV-2), a novel coronavirus disease which emerged in Wuhan, Hubei, China in early December 2019 has now spread around the world [1]. The outbreak was reported to the World Health Organisation (WHO) country office on 31st December, 2019 [2-4] which named the new coronavirus disease as Coronavirus Disease of 2019 (COVID-19) [5-7]. Following reports of increased spread and number of confirmed cases and deaths, on 30th January, WHO declared the outbreak a Public Health Emergency of International Concern [7]. With more countries affected, on 11th March, the WHO Director General described COVID-19 as a pandemic [7]. With the disease being reported among health workers and patients with no history of contact with sea food, the mode of transmission of the virus was established as person-to-person through surfaces, droplets and aerosols produced during coughing [8,9]. As of 23rd January, 2022, more than 349 million cases and 5.59 million deaths have been reported globally [10].

The first confirmed cases of COVID-19 in Zambia were reported on 18th March, 2020 and involved a Zambian couple that had travelled and stayed in France for 10 days [11]. Since then, clusters of confirmed cases were reported from Lusaka city and Kafue town. Before long, cases were reported from the Copperbelt province, Nakonde and Chirundu borders with Tanzania and Zimbabwe, respectively, and eventually from all the ten provinces in the country. To date, more than 300 000 cases and 3,900 deaths have been reported in the country [11]. Following reports of the initial cases, the Zambian government enacted statutory instrument number 22 [12] and instituted prevention control measures including social distancing, restriction of social gatherings by closing schools, bars and casinos, restaurants and other businesses; wearing of face masks, washing hands and sanitising. Messages on COVID-19 transmission, prevention and control were disseminated through mass media. Other measures included restriction of international travel as well as closing of international airports and restricting flights to one (Kenneth Kaunda) international airport; screening and testing; contact tracing, quarantine and isolation, and case management. The restrictive measures were intended to reduce the spread of the virus through social mixing and person-to-person contact which promote spread of infectious diseases, including COVID-19 [12].

Social distancing and restriction of social gathering measures have often been used in epidemics [13] because they encourage the general public to avoid crowded places and drastically shift social mixing patterns [13-16]. For example, in their study conducted in China, Qui and colleagues [16] showed that social mixing patterns affect the trajectory of the outbreak within the local community. Social mixing and networks, frequency of contacts in different age groups and locations−such as schools, churches,

For peer review only - http://bmjopen.bmj.com/site/about/guidelines.xhtml

workplaces, households, bars, and other social gatherings−have a direct effect on the time-dependent and basic reproduction numbers facilitating SARS-CoV-2 transmission in the community [17].

Currently, there is limited evidence on the extent to which these interventions are adhered to in the community. Moreover, community members' perspectives and attitude towards these measures are not known. The aim of this study was to explore the health workers' and community members' perspectives on the factors influencing transmission of COVID-19 in Zambia. Information is needed to inform design and implementation of interventions focusing on reducing transmission of the virus in the country.

Methods

Study design

An exploratory phenomenological qualitative study was conducted using in-depth interviews (IDIs) as a data collection technique over a period of twelve weeks, from 3rd August to 30th October, 2020. Use of IDIs allowed for a detailed exploration of the subject under investigation [18, 19].

Study setting

The study was conducted in 4 primary health care facilities and their local communities: 3 from Lusaka city and 1 from Chirundu district under Lusaka province. Lusaka is the capital city of Zambia and has an estimated population of 2.9 million [20]. Chirundu is an international border town between Zambia and Zimbabwe. It is located 115km south-east of Lusaka city and has an estimated population of 127, 600 [20]. Selection of the two sites was done purposively; Lusaka was included in the study because it was an epicenter of the COVID-19 during the first wave. Chirundu town was selected because it was one of the COVID-19 hot spots in the country and it is a town of entry from the bordering countries to the southern African countries of Zimbabwe, Mozambique, Swaziland, Lesotho and South Africa−at the time, South Africa had the highest prevalence of COVID-19 in the region [21]. With regard to health services, Lusaka has 2 tertiary, 5 second level and several primary level health care facilities. In addition, there are private and faith-based health care providers. Chirundu has one faith-based second level hospital and several primary health care level facilities. The Ministry of Health headquarters and the Zambia National Public Health Institute (ZNPHI)−responsible for the COVID-19 policies, guidelines and disease intelligence are also located in Lusaka. Services provided include specialized and primary health care, including health promotion on COVID-19, screening, testing and contact racing, case management and vaccination.

Participants and sampling technique

For peer review only - http://bmjopen.bmj.com/site/about/guidelines.xhtml

A purposive sampling technique was used to select 60 study participants. Purposive sampling allows for selection of participants with similar experiences regarding the health problem under investigation (that is, COVID-19), while, at the same time, allowing for recruitment of participants with different demographic and socioeconomic characteristics−such as place of residence, occupation and income levels. This, in turn, helps provide insight into the similarities and differences in the participants' experiences with regard to the health problem under investigation [18, 19].

Study participants comprised 15 health workers (7 nurses and 8 public health officers) and 45 community members (18 public market traders, 6 bus drivers and 6 international truck drivers, 3 civic leaders, 3 religious leaders, 3 immigration officers, and 6 community members who had recovered from COVID-19). Health workers were sampled from 4 primary health facilities (3 from Lusaka and 1 from Chirundu border town). Eight health professionals worked in the primary health facilities, four worked as port health staff at the Lusaka International Airport; and the remainder worked as surveillance officers at the Zambia National Public Health Institute (ZNPHI). Community members were recruited from the local communities under the four primary health facilities. International truck drivers were selected from Chirundu border; bus drivers were selected from Lusaka intercity bus station. In order to provide insight into the similarities and differences in the IDI participants' perspectives and lived experiences with COVID-19, we sought to balance the number of participants by place of residence and occupation [18, 19].

To be included into the study, participants needed to be:

- Aged above 18 years
- Residing in the selected area for more than 3 months (except for the international truck drivers sampled from Chirundu border)
- Health workers involved in the COVID-19 program from the selected primary health facilities, international airport and ZNPHI
- Bus drivers from Lusaka

Those who were aged less than 18 years and new in the area were not eligible to participate in the study.

Data collection procedures

Four research assistants were trained in interviewing techniques over a period of 5 days. The training comprised two phases: 3 days of theory and 2 days of practical fieldwork. Efforts were made to select research assistants who were skilled and experienced in qualitative research and spoke both English and the local languages, Nyanja, Bemba or Tonga.

For peer review only - http://bmjopen.bmj.com/site/about/guidelines.xhtml

Interviews were conducted at the participant's preferred place, including the place of residence or office. On average, each interview lasted between 1 hour and 1.5hrours. To ensure quality in data collection, a digital voice recorder was used. Each interview was conducted by a pair of research assistants: one facilitated the interview and took notes, the other one was in charge of the digital voice recorder. Before each interview, written informed consent was obtained from each participant; those who could not read or write were asked to mark with an 'X'. To make it easy for the study participants to understand, the consent form (**Supplementary material *1***) was translated into the local language (Tonga and Nyanja). Before the actual interview, each respondent was asked to complete a short demographic questionnaire. Research assistants read the consent form and the questionnaire and filled it in for those who could not read.

Data collection instruments

Data was collected using a paper- based, unstructured interviewed guide (***supplementary material 2***) that was developed by the research team. The interview guide had two pre-determined themes including 1) perspectives on COVID-19, 2) factors affecting transmission of COVID-19. In addition, a short questionnaire (**Supplementary material 3**) was prepared to collect participant demographic and socioeconomic data. To ensure internal validity, both the interview guide and the short questionnaire went through a rigorous development process. First, the principal investigator with vast experience in qualitative research and familiar with the subject, drafted the initial versions. The two themes in the interview guide were adapted from various sources, including the funding opportunity announcement (FOA) from the National Science Technology Council (NSTC) website, the organisation that funded the research, review of the available literature on COVID-19 and researchers' experience in qualitative research methods. Next, the documents were shared with the research team members for their comments and feedback. The documents were revised based on the research team's comments. Two independent bilingual experts translated the documents into the local languages, Tonga and Nyanga. The translated documents were pre-tested during research assistant training and revised accordingly.

Biosafety

To ensure their safety, research team members and study participants were provided with disposable facemasks and hand sanitisers, which they were encouraged to use consistently during training, travel and data collection process. Moreover, research team members were encouraged to observe physical distancing during training and data collection.

Ethical approval

For peer review only - http://bmjopen.bmj.com/site/about/guidelines.xhtml

Ethical approval was obtained from the University of Zambia Biomedical Research Ethics Committee (UNZA BREC, ref 1132-2020); permission to conduct the study was obtained from the National Health Research Authority.

Data processing and analysis

Voice recordings from the IDIs were transcribed and translated into English by two research assistants who were proficient both in English and the local language. To check for accuracy, members of the research team back−translated 10% of the transcripts into the local language and back into English. These versions were then compared for differences and similarities while listening to the original voice recording. After verification of accuracy in transcription and translation, each transcript was then thoroughly read by one research assistant while the other one listened to the corresponding voice recording. Each translated transcript was then compared with the hand-written field notes that the research assistants prepared during the IDIs. After proof-reading and making corrections, the transcripts were saved on a password-protected computer file kept by the PI for safety. The Word documents were then imported into Nvivo 11 MAC for coding and analysis, and the categories and key sub-themes were identified (supplementary material 2). Coding was done independently by two research team members experienced in qualitative research analysis. The derived codes and categories were later compiled and compared for differences and similarities. In order to make it easy to compare differences and similarities in the respondent perspectives by group (health worker versus community members) and residential area (urban versus rural), separate analyse were done. An inductive approach was used to analyse the qualitative data in N-Vivo QSR 12, and sub-themes were derived by content-analysis. The inductive approach ensured that sub-themes were derived from the pre-determined themes by content-analysis and grouping all similar statements made with respect to particular themes. Quantitative sociodemographic data was analysed using descriptive statistics.

Patient and public involvement

The study design was determined by the call for research proposals on COVID-19 research under the strategic research fund. Thus, the participants and the public were not directly involved in the conceptualisation and design of the study. However, selection of the primary health facilities and study participants was done in collaboration with the provincial and district health managers. First, an inception meeting was held with the ZNPHI and Ministry of Health staff from the Lusaka Provincial Health Office and the two districts to present and discuss the design and objectives of the study. Next, pre-field meetings were held with the provincial and district managers to select primary health care facilities and local communities to be included in the study. Recruitment of participants (health workers and community

For peer review only - http://bmjopen.bmj.com/site/about/guidelines.xhtml

For peer review only

members) was done by the by the primary health facility managers and local community leaders. Finally, a report was written and shared with key stakeholders, including the funding organisation and Ministry of Health. In addition, a dissemination meeting was held in Lusaka to share the results with the Ministry of Health and community leaders in the districts where the study was conducted.

Results

In this section, we present our study findings in three parts: summary of the participants' demographic characteristics (part one); health workers' and community members' perspectives on COVID-19 (part two); and the factors which influence transmission of COVID-19 (part three).

Demographic characteristics

The majority (60%) of the participants were female, the mean age was just above 38.12 years, and they had, on average, between two and three children. Over half (65%) of the participants were married. One third of the participants (33.3%) had secondary school education, one-fifth (21.7%) had tertiary level education, and 1.67% had never attended school at all. Concerning level of monthly income, 20% had an average income of less than K500 per month. Majority of the sample (51.7%) mentioned having had travelled out of town and 5% out of the country. The countries travelled to were South Africa, Zimbabwe and Demographic Republic of Congo. The detailed demographic and socioeconomic profile of the study participants is shown in table 1 below.

Table 1: Sociodemographic characteristics (n=60)

**Variable**	**Mean (SD) /n (%)**
Age	38.12 (12.08)
Children	2.9 (2.0)
Number at home	5.9 (3.35)
Sex	
Female	36 (60%)
Male	24 (40%)
Marital status	
Single	12 (20%)
Separated	4 (6.67%)
Married	39 (65%)
Widow	5 (8.33%)
District	
Lusaka	36 (60%)
Chirundu	24 (40%)
Group	
Health Worker	15 (25%)
Community member	45 (75%)

For peer review only - http://bmjopen.bmj.com/site/about/guidelines.xhtml

Level of education	
Never attended	1 (1.67%)
Lower primary (1-4)	4 (6.67%
Upper primary (5-7)	12 (20%)
Junior Secondary (8-9)	10 (16.67%)
Senior Secondary (10-12)	20 (33.33%)
College	8 (13.33%)
University	5 (8.33%)
Occupation	
Health worker	15 (25%)
Community member	45 (75.0%)
Level of income	
<K500	12 (20.0%)
K500-999	15 (25.0%)
K1000-1499	5 (8.33%)
K1, 500-1, 999	3 (5.0%)
>K2000	25 (41.67%)
Travelled out of town	
Yes	31 (51.67%)
No	29 (48.33%)
Travelled out of the Country	
Yes	3 (5.0%)
No	57 (95.0%)
Countries travelled to	
South Africa, Zimbabwe, DRC	

Theme 1: Perspectives on COVID-19

In this theme, we present the findings on the health workers' and community members' perspectives on COVID-19 including awareness about the COVID-19, sources of information, myths and misconceptions about the disease and knowledge regarding preventive measures.

Awareness about COVID-19

All the 15 health workers and most (60%) community members (except some bus drivers and public market traders) from both study sites were aware of COVID-19 and explained that they had seen on TV how it had spread to many countries and that people in other countries had died from the disease.

"When you watch on TV you can see the massive deaths which have happened in other countries, it shows that there is COVID" (Community member, Immigration Officer, Chirundu district)

In contrast, some community members, especially public market traders and bus drivers from Lusaka mentioned that, although they had heard about people dying, they did not know whether the deaths were due to COVID-19 or not. They also explained that some people did not believe the disease existed

For peer review only - http://bmjopen.bmj.com/site/about/guidelines.xhtml

because they had not seen it as they did with other diseases like Ebola, and that they would only believe if they saw someone dying from COVID-19. Asked whether they had heard the reports from the Ministry of Health on the number of people who had died in the country, these participants explained that, in Africa COVID-19 did not kill as many people as it did in other continents, and that if COVID-19 really existed in Zambia, most people would have died already.

There is nothing conclusive. I need to see the corpses. Not until I see a dead body of someone and they say this one has died from Covid-19" [Bus driver, Lusaka district].

Sources of information about COVID-19

Both the health workers and community members who had heard about COVID-19 cited the Ministry of Health and the media (radio and TV) as the main sources of information on COVID-19. Especially the health workers, civic leaders and people who had recovered from COVID-19 explained that the information included the mode of transmission of the virus, the preventive and control measures and the need to adhere to the guidelines. By contrast, some participants, especially community members interviewed in Chirundu, explained that it was difficult to access information on COVID-19. They also expressed ignorance with regard to the source of the COVID-19 and the symptoms it causes.

The doctor [Minister of Health] said it on TV. He said that the disease is real and that the number of people with COVID-19 in the country has increased [Community member, Lusaka district]

Myths and misconceptions about COVID-19

Study participants expressed different views on beliefs about the existence, origin and mode of transmission of the disease. Most health workers believed that the disease was real and that it was caused by the virus spread through the air and contact with other people and objects. In contrast, community members, including public market traders and bus drivers expressed myths and misconceptions about the COVID-19. They believed that someone had created the disease in order to eliminate the African population, and that foreigners [Chinese] who were interested in opening mines in African countries, like Congo, brought the disease so that local people could die, and that, eventually, the foreigners would take over the mines. Further, some community members including religious leaders believed that the disease was a mark of the beast recorded in the Bible (Revelation 13). They explained that most people did not believe COVID-19 was real, and that government was playing politics and that politicians were working with foreigners to gain their support and make money for themselves. Some attributed COVID-19 to radiation from cell phones and industries, and that most people died because of radiation from 5G Networks. Consequently, they wondered why they were being asked to test for the disease and to wear facemasks.

For peer review only - http://bmjopen.bmj.com/site/about/guidelines.xhtml

For peer review only

"I am completely confused, is it really a natural disease? I think it is a fabricated disease or something like that. It all came from the Chinse people, and that other people are just inheriting it" (Public market trader, Chirundu district).

Knowledge about COVID-19 Preventive Measures

Health workers and community members expressed contrasting perspectives on their knowledge about preventive and control measures. Most health workers and community members from Lusaka expressed knowledge about the COVID-19 prevention and control guidelines. They explained that they helped disseminate the information in the communities where they worked from and encouraged people to adhere to the guidelines including wearing facemasks, social distancing and avoiding overcrowded places, ensuring hand washing and sanitising. Conversely, most community members including those from Chirundu border, expressed ignorance about the preventive and control measures. They explained that it was difficult to get information about COVID-19 because of the limited access to TV, radio, internet and mobile phone network. Especially bus drivers and public market traders mentioned that they only hear from people in the community and that most information was incorrect.

"They tell us to wear face masks and wash our hands with soap, but most of us do not have anyone to explain to us" (Public Market Trader, Lusaka city)

Theme 2: Factors influencing transmission of COVID-19

In this section, we present the findings on the factors that influence transmission of COVID-19 in the community. They include living and working conditions, travel and social movement patterns, and social networks.

Living and working conditions

Both health workers and community members (i.e., those who believed in the existence of the disease) were unanimous on living and working conditions being important factors driving the COVID-19 transmission. Overcrowding in some homes and work places made it difficult to maintain social distance. Public health officers explained that staff shared the office space and majority did not have enough hand-washing or sanitising facilities; they only used one shared sanitizer and the hand washing facility. They added that some offices and homes did not have running water; where it was present, the supply was erratic and made it difficult for people to follow the guidelines on hand-washing and other hygienic purposes.

"You know our tradition, parents use their own bedroom, then the kids, maybe there are 5 girls, and they use one bedroom. If there are 6 boys they will use one bedroom" [Immigration officer, Chirundu district].

For peer review only - http://bmjopen.bmj.com/site/about/guidelines.xhtml

Attitude towards preventive measures

Participants expressed different attitudes towards the preventive measures. Health workers and some community members (international truck drivers and those who had recovered from or seen someone with COVID-19 expressed) a positive attitude towards preventive measures. They explained that the measures were beneficial to the population. The main benefits were protection against the virus, helping people maintain hygienic standards in the community. They explained that, in the beginning, community members never believed in the reality of the disease until they noticed that even in Zambia, people started dying; some were being quarantined. Health workers explained that there was a need for people to follow the prevention and control guidelines from the Ministry of Health, especially during the cold season, when the risk of transmission of the virus was high.

"What I think is that COVID is there, so the most important thing is to just follow what they say we should do" ***[Public Health Officer, Lusaka district]***

In contrast, most community members expressed a negative attitude towards the COVID-19 preventive measures. They wondered why they were being asked to wear facemasks or avoid mixing with their friends or attending important social functions like church services, weddings or drinking parties. Some participants also explained that it was not clear why even children were told to wash their hands or wear facemasks.

"To be honest people do not like these things, they do not follow because they say, it is a lie, Corona is not there. They are saying because there is no Corona so they do not wear the mask" [Community member, Lusaka district].

Adherence to preventive measures

There was consensus among the participants concerning non-adherence to preventive measures in the community−which was seen as one of the important factors contributing to the spread of COVID-19 in the population. Health workers observed that, although some people followed the guidelines−wore facemasks and used hand-sanitisers− majority did not. They had continued mixing and interacting as before, especially in the markets and buses. Asked why they did not adhere to the guidelines, most community members explained that people in their communities believed that preventive measures came from other countries and local people, especially Africans, were just being told to follow. They also explained that most people were discouraged by what they saw among political leaders who held meetings and political rallies without following the guidelines. They explained that politicians "played politics" as they did not maintain social distance or wear facemasks when holding pre-election political

For peer review only - http://bmjopen.bmj.com/site/about/guidelines.xhtml

rallies. Similarly, most bus drivers confirmed that most passengers did not observe the guidelines when travelling on public transport.

Participants had consensus on the importance of social gatherings such as weddings, kitchen parties, church services, taverns and funerals as virus super spreader events. Nevertheless, they observed that nothing had changed with regard to social mixing patterns; people were used to their old way of life of greeting and hugging their friends when they met. They argued that people did not feel good to stand a meter or an arm apart because of the new rules. Similarly, participants, especially health workers, civic and religious leaders, confirmed that most families had continued visiting and inviting friends or extended family members to their homes for family and social functions such as parties and beer drinking. Although some people did not attend funerals and only provided monetary and other material support, many community members did. Further, shops and churches remained open and people continued going to the market, shops for their businesses, shopping and attending church services. .

"The only time we can say social distance, is if we come to the market, in the shops, or church. There is no social distance when you go to the political rallies" [Religious leader, Lusaka district]

Travel & movement patterns

Both health workers and most community members (except some bus drivers) confirmed that travel within and outside the country was a major factor influencing transmission of the virus. They observed that, despite the COVID-19 situation, local and international travel remained the same. Public transport on buses and minibuses was the commonest mode of transport. Those who owned cars explained that they travelled in their personal cars. Reasons cited for travel were work, business, shopping, family matters (such as funerals), and other purposes.

Bus drivers explained that although they allowed fewer passengers than before, most minibuses still loaded to full capacity−this made it difficult to maintain social distance on buses. Moreover, most passengers did not use facemasks. By contrast, some participants, especially community members and bus drivers, argued that travel did not put them at risk of infection because, before boarding on buses, passengers were made to wear facemasks and that those who did not have were not allowed to board.

"I have seen that there is no social distance on those buses. But when driving yourself yeah, there is protection because you know you are alone or maybe just the two of you, you mask up" [Immigration Officer, Chirundu border].

For peer review only - http://bmjopen.bmj.com/site/about/guidelines.xhtml

International travel was perceived as an important driver of the COVID-19 transmission. Although some countries had closed their borders, participants observed that travel between countries, especially by road, continued. They explained that, international truck drivers spent many days away from home, in foreign countries and on transit. . . One driver explained that, within a space of two weeks, he had been to many countries including South Africa, Zimba, Zambia and DR Congo. Although they sanitise during travel, interaction with a lot of people and handling papers during transit and at the borders, put drivers and other travellers at an increased risk of getting infected or spreading the infection to others

"I am always moving to Zambia, Congo, South Africa, just like that. I was in Congo and then I connected to South Africa, that same month. I spent somewhere like 2 weeks in South Africa, then I came back" [International truck driver, Chirundu border]

Social network

Social networks were also cited as an important factor contributing to the spread of COVID-19 infection in the community. Although the frequency had reduced, people still visited their friends, workmates and relatives, and their friends. They explained that, especially, workmates visited in the evening when they knocked off and that in some instances they visited twice or thrice in a week.

"*If I leave my place I go to her place and if you are to find me, mainly I am there. If I don't go to her place, she will come here, we chat and chat**" [Public market trader, Lusaka district].***

Conversely, some participants explained that they no longer went out to interact with their friends as much as they used to before the COVID pandemic. They mentioned that they had stopped attending church services and stayed at home to avoid contracting and spreading the virus. In addition, some participants explained that, although they visited family members and went to specific places for their businesses, they avoided physical meetings and gatherings; they only communicated on the phone, and that if people went home, they would only greet and not spend time together.

***"From the time the issues of corona started I have never attended a wedding ceremony or funeral issues, I have never attended"* [Public market trader*, Lusaka district*].**

Discussion

The aim of this study was to explore the health workers' and community members' perspectives on the factors influencing transmission of COVID-19 in Zambia. Overall, our findings suggest that most people were aware of the COVID-19 pandemic and the preventive measures. Nevertheless, several factors including myths and misconceptions and a low risk perception about COVID-19, a negative attitude

For peer review only - http://bmjopen.bmj.com/site/about/guidelines.xhtml

towards the preventive and control measures, movement and travel patterns, working and living conditions, social gatherings, networks and interactions affected adherence to the preventive and control measures and increased the risk of COVID-19 transmission in the community.

Our findings suggest that, people were generally aware of the COVID-19 disease and the associated preventive and control measures.− The Ministry of Health, media (TV, radio, internet and social media) and personal experience seem to have been the important sources of information about COVID-19 and preventive measures. This finding corroborate a previous study conducted in Egypt and Nigeria [22] which showed that, despite not adhering to preventive measures, most people have satisfactory knowledge about COVID-19 and the preventive and control measures. Nevertheless, our findings show that limited access to information on COVID-19 appear to be a major challenge, especially in rural communities where TV, radio and mobile phone reception and internet signal are poor. Consequently, a lack of information on COVID-19 seems to have led to widespread myths and misconceptions about the new disease−its existence, origin and severity−and ultimately influenced people's attitude towards prevention and control measures. Further, people's attitude towards the COVID-19 preventive measures seem to affect adherence to preventive and control measures. Participants expressing such strongly held beliefs doubted the existence of the virus and the COVID-19 disease; they expressed a negative attitude towards the preventive measures. They also expressed a low risk perception towards the severity of and their personal susceptibility to the disease. These findings highlight the importance of attitude and risk perception in influencing people's decision to adopt healthy behaviours− such as COVID-19 preventive measures [23, 24]. Our findings suggest that, non-adherence to preventive measures was a major contributing factor to the spread of the virus in the community. Interventions should focusing on preventing and mitigating myths and misconceptions about COVID-19 by providing accessible correct, consistent and timely information on COVID-19 in rural areas− through improved TV, radio reception, and internet and mobile phone connectivity. Interventions should also focus on provision of information about the benefits of adherence to prevention and control measures in order to increase community members' attitude and personalised risk perception. Interventions focusing on improving personalised risk perception, behavioural beliefs and attitude have been shown to increase adoption of and adherence to a target health behaviour [24].

Life style, living and work conditions were identified as important drivers of the COVID-19 transmission. For example, the family size and living conditions in most homes−especially in high density urban neighbourhoods and rural areas where a number of family members shared sleeping spaces −made it difficult to practice social distancing in the home. Similarly, our findings suggest that sharing and

For peer review only - http://bmjopen.bmj.com/site/about/guidelines.xhtml

overcrowded offices, poor water supply and inadequate sanitisers and washing facilities in places of work posed an important risk for the transmission of the virus in the work setup. Moreover, poor water supply, and overcrowding, made it difficult to observe social distance and adherence to hand washing and hygienic practices, especially in public markets. These findings corroborate previous studies [25-27]. For example, a systematic review of 68 studies conducted by Zar and colleagues [26] found that poor living conditions in low and middle income countries [LMICs], including lack of adequate sanitary facilities, running water and overcrowding pose challenges to adherence to prevention and control guidelines among urban slum residents and facilitate transmission of SARS-CoV-2. Further, Ahmad and others [27] reported a higher risk of infection and death from COVID-19 in counties with poor housing compared to those with modern housing facilities. Policies and intervention on COVID-19 prevention would benefit from improved housing, work condition and sanitation and water supply.

Moreover, local and international travel patterns were found to be a major risk factor for community transmission and importation of cases into the country, respectively. Although a number of people used private transport for local travel when going for business, work, family matters and other purposes, majority travelled on public transport such as buses and minibuses where adherence to social distance is difficult. Although international travel by the general public had reduced at the time of the study, travel by road was continued as before. Interaction with their fellow drivers and other travellers as well as customs and immigration officers all put international truck drivers at an increased risk of contracting and transmitting the disease. These findings corroborate previous studies [17, 28] which highlighted the importance of international and local travel in influencing spread of the virus. For example, in their study [28] conducted in the USA, Davis and colleagues reported international travel as the key driver of the introduction of SARS-CoV-2 in the West and East Coast metropolitan areas that could have been seeded as early as late-December, 2019. The study also showed that for most of the continental states the largest contribution of imported infections arrived through domestic travel flows. Similarly, a study conducted in China [17] showed that although travel to foreign countries was responsible for the importation of initial infections into the country, social mixing patterns affected the trajectory of the outbreak within the local community. Our findings highlight the benefits to be gained from interventions focusing on limiting social movement through restriction of local and international travel.

Finally, our findings suggest that social networks, frequency and duration of contacts are an important risk factor for the spread of the virus. Despite the government guidelines on restricting social gatherings, most people maintained their social networks through social functions such as wedding ceremonies, church services, kitchen parties, funerals, casinos and bars, and home visitations. This finding

For peer review only - http://bmjopen.bmj.com/site/about/guidelines.xhtml

corroborate previous studies [17, 29] which reported the importance of social gatherings in facilitating spread of the virus and increasing the basic reproductive number. For example, a study by Alimohamdi and colleagues conducted during the early phase of the pandemic highlighted the effect of social interaction and networks in different age groups and locations on the time-dependent and basic reproduction numbers governing SARS-CoV-2 transmission in the community [29]. The study concluded that reducing the number of contacts within the population is a necessary step to control the epidemic. Thus, policies regulating social interactions could help reduce transmission of the virus.

Limitations of our study should be noted. First, like all qualitative studies, our findings are based on the few IDI participants' views. We could not conduct focus group discussions to compare and confirm the findings due to safety considerations and logistical challenges. Further, the study was conducted during the first wave of the COVID-19 pandemic when the disease was still new and the number of cases and deaths was still low in the country. Zambia has experienced four successive waves. It is not clear whether community members' and health workers' perspectives have changed over these successive waves of COVID-19 in the country. However, the selected participants' perspectives may not have been representative of the views of the other members of the community, especially those who had tested positive to the virus and experienced it. Although Chirundu and Lusaka districts had one of the highest number of COVID-19 cases, most study participants had not experienced the disease. The reason for this selection was to have participants who could provide insightful information on the disease, and not necessarily having experienced it.

Despite these limitations, our study has several strengths. First, the study provides the first in-depth assessment and analysis of the health workers' and community members' perspectives on the factors influencing transmission of the virus in the two districts that were hotspots for COVID-19 during the first wave in Zambia. Second, triangulation through our purposive selection of the study participants comprising health workers and community members from both urban and rural sites, with varied socioeconomic backgrounds and experiences with regard to COVID-19, as well as the use of trained research assistants and rigours data analysis conducted by a trained and experienced team minimised bias and increased the internal validity of the study findings. Finally, our findings provide important insights into the factors influencing transmission of COVID-19 in Zambia can serve as important targets for policy and intervention design for prevention of COVID-19 transmission in Zambia and other countries with similar demographic and epidemiological contexts. As far as we know, this is the first qualitative study conducted on this subject in Zambia.

For peer review only - http://bmjopen.bmj.com/site/about/guidelines.xhtml

Conclusion

These findings highlight important factors influencing transmission of the coronavirus in Zambia including limited information, about COVID-19, myths and misconceptions about the disease, negative attitude towards and non-adherence to prevention and control measures, negative behaviour of the role models such as politicians and other important community leaders, living and working conditions, travel and movement patterns, social gatherings, networks and interactions. Future prevention interventions should focus on: a) increasing access to information about COVID-19; b) increasing people's risk perception and personal susceptibility to the disease, c) improving attitude towards the benefits of preventive and control interventions, d) limiting local and international travel; e) restricting social gatherings, and f) mitigating structural and socioeconomic barriers−by improving housing and work conditions, provision of safe water and sanitary facilities− in order to increase adoption of and adherence to preventive measures.

Finally, further research, in form of survey with a quantitative design, is needed to measure people's attitude towards prevention measures and determine the levels of adherence to the life-saving interventions. Research with a longitudinal design is also required to test whether adherence to the preventive and control measures actually leads to reduced transmission of the Corona virus and improved health outcomes. Findings from such studies can serve as basis for design of public health policy on preventive and control interventions as well as advocacy for adherence to these measures. Currently, this evidence is non-existent.

Competing Interest

We declare that as authors we have no conflicting interest in this work.

Availability of data and materials

Data are available upon reasonable request from the corresponding author and with permission of the UNZABREC Institutional review board.

Acknowledgements

We thank Eden University administration, Dr Flavien N. Bumbangi and Mr Kennedy Chishimba for the logistical support they provided during the training of data collectors and data collection process. Our gratitude also goes to the study participants for their valuable time and input into the study.

For peer review only - http://bmjopen.bmj.com/site/about/guidelines.xhtml

Authors' Contributions

All authors designed the study. Under the oversight of CS, NM and LL, HKK, MN, supervised the data collection process. CS and DCS conducted data analysis. CS wrote the first draft of the manuscript. PIS, NM and DCS revised the manuscript. FMG advised on the final manuscript. All authors read, commented on and approved the final manuscript.

Funding

This work was supported by the National Science and Technology Council (grant number NSTC 101/6/8), as part of the Emergency COVID-19 research grant under the strategic research fund.

References

1. Huang C, Wang Y, Li X, Ren L, Zhao J, Hu y et al. 2020. Clinical features of patients infected with 2019 novel coronavirus in Wuhan, China. The Lancet 395 (10223): 497-506

2. Prem K, Liu Y, Russell TW, Kucharski A, Eggo RM, Davis et 2020. The effect of control strategies to reduce social mixing on outcomes of the COVID-19 epidemic in Wuhan, China: a modelling study.
3. World Health Organisation.2020. Coronavirus Disease (COVID-19 Pandemic. Available at: https://www.who.int/emergencies/diseases/novel-coronavirus-2019, accessed on 5th September, 2021.
4. Zhang J, Litvinova M, Wang W, Wang Y, Deng X, Chen X et al. 2020. Evolving epidemiology and transmission dynamics of coronavirus disease 2019 outside Hubei province, China: a descriptive and modelling study. The Lancet Infectious Diseases. Available at: :https://doi.org/10.1016/S1473-3099(20)30230-9, accessed on 6th September, 2021

5. Ksiazek TG, Erdman D, Goldsmith CS, et al. A novel coronavirus associated with severe acute respiratory syndrome. N Engl J Med2003; 348: 1953–66.3

6. Kuiken T, Fouchier RAM, Schutten M, et al. Newly discovered coronavirus as the primary cause of severe acute respiratory syndrome. Lancet 2003; 362: 263–70.4

7. Drosten C, Günther S, Preiser W, et al. Identification of a novel coronavirus in patients with severe acute respiratory syndrome. N Engl J Med 2003; 348: 1967–76

For peer review only - http://bmjopen.bmj.com/site/about/guidelines.xhtml

8. World Health Organisation.2020. WHO timeline-COVID-19. Available at https://www.who.int/news-room/detail/27-04-2020-who-timeline---covid-19, accessed on 6th September, 2021

9. Li Q, Guan X, Wu, P, Wang, X., Zhou L, Tong Y et al. 2020. Early transmission dynamics in Wuhan, China, of novel coronavirus-infected pneumonia. *New England Journal of Medicine*. 382:1199-1207

10. Wiley online library.2020. COVID-19 Novel Coronavirus Outbreak. Available at: https://novel-coronavirus.onlinelibrary.wiley.com/, accessed on 6th September, 2021

11. National Institute for Communicable Diseases. 2020. Available at: https://www.nicd.ac.za/first-case-of-covid-19-coronavirus-reported-in-sa/, accessed on 6th September, 2021

12. Ministry of Health. 2020. Available at: https://web.facebook.com/pg/mohzambia/posts/

13. Ministry of Health. Public Health (Infected Areas)(Coronavirus Disease 2019) Regulation, 2020. Statutory Instrument no.22 of 2020. Available at: https://www.enotices.co.zm/download/statutory-instrument-no-22-of-2020-the-public-health-infected-areas-coronavirus-disease-2019-regulations-pdf/

14. Kucharsk AJ, Russell TW, Diamond C, Liu Y, Edmunds J, Funk S et al. 2020. Early dynamics of transmission and control of COVID-19: a mathematical modelling study. The Lancet Infectious Diseases. 20 (5):553-558

15. Qui Y, Chen X and Shi W. 2020. Impacts of Social and Economic Factors on the Transmission of Coronavirus Disease 2019 (COVID-19) in China. British Medical Journal. doi: https://doi.org/10.1101/2020.03.13.20035238

16. Day M.2020. Covid-19: four fifths of cases are asymptomatic, China figures indicate. British Medical Journal. 369:m1375 doi: 10.1136/bmj.m1375

17. Al-Raeei M. The basic reproduction number of the new coronavirus pandemic with mortality for India, the Syrian Arab Republic, the United States, Yemen, China, France, Nigeria and Russia with different rate of cases. Clinical Epidemiology and Global Health, 2020; 9: 147-149

18. King N and Horrocks C. 2010. Interviews in Qualitative Research. 1st Edition. SAGE Publications ltd. 25-41.

19. Ary D, Jacobs L, Sorensen C and Walker D. Introduction to research in education. 9th edition. Wadsworth, CA: Cengage Learning, 2013

For peer review only - http://bmjopen.bmj.com/site/about/guidelines.xhtml

20. Central Statistical Office Zambia. 2010 census of population and housing - Southern Province analytical report. Lusaka: Central Statistical Office Nationalist; 2014. Available at: http://web0.psa.gov.ph/statistics/census/population-and-housing.

21. World Health Organisation, Africa, 2020. Coronavirus (COVID-19). Available, https://www.afro.who.int/health-topics/coronavirus-covid-19, accessed on 20th September, 2021

22. Hager E, Odetokun IA, Bolarinwa O, Zainab A, Okechukwu O, Al-Mustapha AI. Knowledge, attitude, and perceptions towards the 2019 Coronavirus Pandemic: A bi-national survey in Africa. PLoS ONE 15(7), 2020; e0236918. https://doi. org/10.1371/journal.pone.0236918

23. Cooke R and French DP. How well do the theory of reasoned action and the theory of planned behaviour predict intentions and attendance at screening programmes? A meta-analysis. Psychology & Health, 2008; 23: 745-765.

24. Glanz K, Rimer BK, Viswanath K. Health Behaviour and Health Education: Theory, Research and Practice (4th ed.). San Francisco: Jossey-Bass, 2008; 69-95.

25. Zar HJ, Dawa J, Fischer GB & Castro-Rodriguez JA (2020). Challenges of COVID-19 in children in low- and middle-income countries. Paediatric Respiratory Reviews 35: 70-74 https://doi.org/10.1016/j.prrv.2020.06.016.

26. Wasdani KP & Prasad A (2020) The impossibility of social distancing among the urban poor: the case of an Indian slum in the times of COVID-19, Local Environment, 25:5, 414-418, DOI: 10.1080/13549839.2020.1754375

27. Ahmad K, Erqou S, Shah N, Nazir U, Morrison AR, et al. (2020) Association of poor housing conditions with COVID-19 incidence and mortality across US counties. PLOS ONE 15(11): e0241327. https://doi.org/10.1371/journal.pone.0241327

28. Davis JT, Chinazzi M, Perra N, Mu K, Piontti APY, Ajelli M. Estimating the establishment of local transmission and the cryptic phase of the COVID-19 pandemic in the USA, 2020; 28:2020.07.06.20140285. doi: 10.1101/2020.07.06.20140285. PMID: 32676609; PMCID: PMC7359534

29. Alimohamadi Y, Taghdir M, Sepandi M. Estimate of the Basic Reproduction Number for COVID-19: A Systematic Review and Meta-analysis. J Prev Med

For peer review only - http://bmjopen.bmj.com/site/about/guidelines.xhtml

Public Health. 2020; 53(3):151-157. doi: 10.3961/jpmph.20.076. Epub 2020 Mar 20. PMID: 32498136; PMCID: PMC7280807.

For peer review only

For peer review only - http://bmjopen.bmj.com/site/about/guidelines.xhtml

INFORMED CONSENT FORM/INFORMATION SHEET

Background

Hello, my name is ___________________________ I am working with the Levy Mwanawasa Medical University and Eden University. This study is funded by the National Science Technology Council.

Purpose

We would like to speak with you because you are ……….. We would like to understand your opinion and perspectives on the ***sociocultural dynamics influencing transmission of coronavirus in Zambia*.** This research is taking place in Lusaka and Chirundu districts. What you tell us may help the Ministry of Health and government as they try to reduce transmission of ***coronavirus*** among the people who live in this community.

Process

You will be one of approximately 65 respondents asked to participate in this discussion. We would like to ask you some questions in order to understand your opinions and perspectives on the ***sociocultural dynamics influencing transmission of coronavirus.*** The discussion should take no longer than 1.5 hours.

To ensure that I don't miss or forget anything during the discussion, your responses to the various questions will be recorded using a digital recorder. We will not write your name, instead we ask you to sign on this form, but we will keep your signature private. Responses from the various questions will be summarised, analysed and a report written. When we write reports from this discussion, we will not show what you said nor are we going to use your name or signature at any time.

Potential Benefits

You will receive no direct benefit from your participation in this discussion. However, your participation may help the Ministry of Health and the government as they try to reduce transmission of ***coronavirus*** in the country

Risks and Discomforts

The risks of taking part in this study are that other people will hear your responses. It is important that you do not share anything that you are not comfortable with. If you or someone in your family had a bad experience, it may be difficult or uncomfortable to remember or share it. You do not have to respond to any question unless you feel comfortable doing so. You are free to stop the discussion at any time if you need to.
.

Alternatives

You can choose not to take part in this discussion. If you decide not to take part or withdraw from this discussion, you will not suffer any penalty or lose any benefits to which you are entitled.

Participant Costs and Payments

You will not be paid to participate in this discussion. There are no costs to you for participating.

Confidentiality

For peer review only - http://bmjopen.bmj.com/site/about/guidelines.xhtml

INFORMED CONSENT FORM/INFORMATION SHEET

Your information will be saved and locked in a computer file and destroyed once it is copied from it. Only study team members will be able to listen to your response. When we write reports from this data, we will not link your signature or identity to your responses.

Participant's Rights

By agreeing to participate in this discussion, you do not waive any of your legal rights. Giving consent means that you have heard or read the information about this discussion and that you agree to participate. You will be given a copy of this form to keep. If you have questions or concerns at any time, you can contact the Principal Investigator, Dr. Sialubanje Cephas on +260-977-441273, the Co-Investigator, Prof Fastone Goma on +260 977 772 301, or any of the staff from the University of Zambia Biomedical Research Ethics Committee on +260-21-1-256067

Right to Refuse or Withdraw

Taking part in this discussion is voluntary. You have the right to refuse to take part. If you decide to be in this discussion, and then change your mind, you can withdraw from it at any time and to skip questions you may deem personal or otherwise without any repercussions. Your participation is voluntary. If you choose to take part, you have the right to stop the discussion at any time.

By signing below you are agreeing to participate in the discussion which indicates that you have read this consent form (or have had it read to you), that your questions have been answered to your satisfaction, and that you voluntarily agree to participate in this research study. You may keep a copy of this for your records.

Signature or thumb print: ____

Date: ____

Signature of Impartial Witness: ____

Date: ____

For peer review only

For peer review only - http://bmjopen.bmj.com/site/about/guidelines.xhtml

INTERVIEW GUIDE

……………………………………………………………………………………………….

Theme 1: Perspectives on COVID-19 preventive measures (social distancing, wearing masks and handwashing)

Q1: Please tell me what you think about the measures government has put in place to prevent transmission of COVID-19

Probe:

- Awareness about COVID-19
- Knowledge about the measures (social distancing, wearing face masks, hand washing)
- Participant's attitude towards these measures (advantages and disadvantages)
- Perspectives on these measures (are the community members adhering to them)

Theme 2: Livelihood and way of life

Q2: Please tell me about your way of life

Probe:

- Family and living conditions (place of residence, number of people at home, number of rooms in the house, condition of the house)
- Source of drinking water (borehole, river, presence of running water)
- Hand sanitising facilities at home
- Occupation/main source of family income
- Working conditions (office space; office shared or not; number of people in the office; use of lifts; toilet facilities, hand sanitising facilities at work)
- Mode of transport to place of work

Theme 3: Travel & movement patterns

Q3: Would you walk me through your recent travel history (past one month)

For peer review only

Probe:

- Movement from home on a typical week day
- Movement from home on a typical weekend day
- Places the respondent visited in the past one month within and outside Zambia
- Duration of stay away from home
- Mode of transport commonly uses/used
- How often the respondent travels/traveled outside home
- The purpose (s) of travel
- People who travel with respondent
- Mode, if any, of protecting oneself against COVID-19 during travel

Theme 4: Social networks

Q4: Walk me through the most important people (family members, friends, relatives, workmates, church mates,) that you have interacted with in the past one month

Probe:

- Most important people frequently in contacts with outside home
- How do you maintain contact with these people (family, friends, workmates, church mates)
- Most recent social activities, meetings, church meetings, weddings, funerals attended in the past one month
- Physical contact with friends & family during social gatherings (i.e. shaking hands, sharing cigarettes/drinks, sharing eating utensils, sharing accommodation/ hotel room)
- Adjustments in physical interaction with family members, friends, workmates (physical/social distancing, working from home, worshipping from home, visits)
- Perspectives on social distancing

THE END

For peer review only

Demographic questionnaire

Questionnaire ID number [ ]

Name of District…………………………………………….

---

1. Place of residence (indicate name)………………………………..
2. Number at home (indicate actual number)………………………..
3. Age (Indicate the actual age in years)………………………….
4. Number of children (indicate the actual number)…………………..
5. Marital status (Circle one appropriate answer)…………
   1) Single
   2) Separated
   3) Married
   4) Widow
   5) Cohabiting
6. Religious denomination (indicate appropriate answer)……………..
   1) Catholic
   2) SDA
   3) Pentecostal
   4) Jehovah's witness
   5) Other (specify)…………..
7. Level of education (highest grade reached) (Circle appropriate answer)
   1) Never attended school
   2) 1-4
   3) 5-7
   4) 8-9
   5) 10-12
   6) College
   7) University
8. Occupation (indicate)……………………
9. Level of income per month (in Zambian Kwacha) (Circle appropriate box)………..
   1) <K500
   2) K500-999
   3) 1000-1499
   4) K1,500-1999
   5) >2000
10. Travel out of town in the past one month……..
    1) Yes
    2) No

For peer review only

11. If yes to Q19, mention the place you travelled to (indicate name)…………………..

12. Have you travelled out of the country in the past one month? ……………………

1) Yes
2) No

13. If yes to Q12, mention the place you travelled to (indicate name of place)……………….

14. Mention the date you travelled (indicate the actual date ………………..

15. Mention the date you returned (indicate the actual date)………………..

16. How long did you stay there (indicate actual duration in days)………..

For peer review only

Standards for Reporting Qualitative Research (SRQR)

**Topic**	**Page number**
**Title and abstract**	
Title	1
Abstract	2
**Introduction**	
Background/Problem formulation	4
Objectives or research question	5
**Methods**	
Study design/qualitative approach	5
Study setting/Context	5
Sampling strategy and participants	6
Data collection methods	6
Data collection instruments and technologies	7
Ethical issues pertaining to human subjects	8
Data processing and analysis	8
Techniques for enhancing trustworthiness	8
**Results/findings**	9
Synthesis and interpretation	
Links to empirical data	
**Discussion**	
Integration with prior work, implications, transferability and contributions to the field	16
Limitations	18
**Other**	
Conclusion	19
Conflict of interest	19
Author contribution	20
Funding	20

For peer review only
